# Supplementary material for: Work time allocation at primary health care level in two regions of Albania
Source: PLoS One. 2022 Oct 26;17(10):e0276184. doi: 10.1371/journal.pone.0276184 (PMC9605026; doi:10.1371/journal.pone.0276184)
Supplement: S1 Table — (DOCX) [file pone.0276184.s001.docx]

**S1 Table. Categories of activities to observe.**

| **Activity category** | **Activity sub- category** | **Activity subordinate sub-category** | **Comments** |
| --- | --- | --- | --- |
| Service provision to users | Child Care | Consultation with Children 0-18 years old | Following up growth and development, anthropometry, examination, treatment and discussion with the patient or their parent/legal guardians. |
| Service provision to users | Child Care | Vaccination | Vaccination of Children according to the National Vaccination Program and advising before and after the vaccine. Informing about the vaccines (only if done at the Consultancy room it goes under this category) |
| Service provision to users | Woman and Reproductive Health Care | Antenatal Care | Consultation with pregnant women and follow up before, during and after pregnancy (up to 40 days), vaccination, education, monitoring the well-being of the mother and the fetus (running tests and the interpretations of the tests) and treatment. |
| Service provision to users | Woman and Reproductive Health Care | Consultation with Woman | Discussion about family planning, examination and treatment for Woman Health related problems |
| Service provision to users | Adult and Adolescent Care | Consultation with patients of age 14-70 | Diagnose, treatment, prevention, referring of patients with acute and chronic problems. (Patients from 14-18 years old are considered adolescents) |
| Service provision to users | Check Up | Check up with patients of age 35-70 | Examination according to “How are you” guideline, informing the population for the check-up, filling up the patient form |
| Service provision to users | Clinical Consultation | 1. Emergency care 2. Elderly care 3. Mental Health care 4. Follow up visit | All direct examination, treatment and discussion with patient that include everything related to elderly people, emergency situations and mental health care problems, follow up visit |
| Service provision to users | Assistance with services |  | Assisting another medical professional with the provision of care; mainly a category for nurses |
| Service provision to users | Medical Procedures |  | Medical manipulation such as injections, intravenous infusion, micro-surgery, measurement of blood pressure or glycemia etc. |
| Service provision to users | Other Patient Consultation | 1. Telephone consultation 2. Consultation on schools and kindergarten 3. Home visits 4. Door to door | 1. Telephone consultations refers to informing, giving advices, answering questions to patients through the telephone. 2. Consultation on schools and kindergarten refers to informing, educating, answering questions to patients in these places 3. Home visits is the clinical consultation held in the patient’s house. It includes examination, diagnose, treatment and informing the patient 4. Door to door refers to informing and advising the patients in a door to door approach i.e. informing for the vaccination, informing for the check up |
| Service provision to users | Health promotion |  | Special events for health promotion or prevention activities, group or individual consoling about promotion and preventing activities at health center, community and during clinical consultation |
| Service provision to users | Prescription | 1. e-prescription 2. Hand-written prescription | 1. Reads or fills out the e-prescription for the reimbursement drugs even if in the same time is talking to the patient 2. Reads or writes down in the hand-written prescription for the non-reimbursement drugs even if in the same time is talking to the patient |
| Service provision to users | Clinical orientation in reception |  | Discussion during the reception and the follow-up of the patient related to the provision of information or answering questions patients might have about their health condition. It includes also the questions the nurse in the reception asks regarding to the patient’s health condition for the performance of a quick diagnosis and the patient’s orientation towards the doctor or the nurse. |
| Service provision to users | Preparation for Patient Care |  | Preparing for the next patient, e.g. personal hygiene and cleaning at the facility for the next patient, equipment’s sterilization |
| Service provision to users | Patient administration | 1. Consultation form 2. Recommendation 3. Patient Form   Administrative work in Reception | 1. Reading or writing in one of the journals/forms that are used to report on the patient consult and medical procedures like consult form and medical procedures form. 2. Filling the e-recommendation or reading the results from the narrow specialist. 3. Reading or writing in the patient form; even if the same time talking to the patient   Includes administrative work of the receptionist like: recording patient’s information, giving bills, hanging in the walls the list of services, prices and promoting materials, saving and archiving documents, attention to the emergency room monthly graphic of doctors and nurses, noting the movement of the health staff |
| Administration | Health Information System | 1. Electronic HIS 2. Paper based HIS 3. Electronic register 4. Hand-written register | Reading or writing in one of the registers that are used to report the patient consult or drug administration, even if the same time talking to the patient   1. Electronic HIS refers to filling the “How are You” form 2. Paper based HIS: includes all the paper-based registers (excluding the consult register) fundamental register for different services, vaccination’s registers (for children, for pregnant women, for population during sessional flue), chronic diseases’ register, medication discharge register, entry-exit register of vaccine supply, women’s screening register over 35 years for Ca MAME, register for the report of child birth, family planning register 3. E-register contains demographic data of the population 4. Hand-written register is referred to consult’s register on which all the daily data of the patients are recorded |
| Administration | Other administration |  | Work related to other administrative things; e.g. reimbursement expense plan, weekly projection of check-up, monthly planning of vaccination, supply, archive and registration of the list of medicines obtained. Also, different documentation proofs that are given to patients like proof of vaccination, medical certification for driving license etc are considered part of other administration work. |
| Continuous Medical Education | Training |  | Receiving or giving trainings from CME cycle to fulfill the mandatory credits to renew his/her license |
| Continuous Medical Education | Professional Reading |  | Medical reading in textbooks or family medicine guidelines |
| Unproductive | Waiting |  | Waiting for patient or something else |
| Unproductive | Personal time |  | Break to eat, private discussions or other personal time; including being invited to eat or drink during home visits |
| Miscellaneous | Walking or Driving |  | Walking or driving for home visits or other |
| Miscellaneous | Rotation |  | Offering service in other health post-ambulatories. Includes the time of going and coming back, as well as all the activities that happen there. |
| Other |  |  | Certain surveillance activities for communicable diseases (case investigation, outbreak response), school vaccinations, immunization activities, hygiene and sanitation |
| Meetings |  | Professional meetings or with Industry representatives | Work related meetings, e.g. group meeting at the facility, or meeting with representative from industry |
| Outreach activities |  |  | Activities done away from the facility: include all other kinds of activities mentioned above |
