# Supplementary material for: Work time allocation at primary health care level in two regions of Albania
Source: PLoS One. 2022 Oct 26;17(10):e0276184. doi: 10.1371/journal.pone.0276184 (PMC9605026; doi:10.1371/journal.pone.0276184)
Supplement: S2 Table — (DOCX) [file pone.0276184.s002.docx]

**S4 Table. Overall time allocation and 95% confidence intervals on categories of activities by health workers.**

| **Category** | **Doctor** | | | **Nurses** | | | **Combined** | | |
| --- | --- | --- | --- | --- | --- | --- | --- | --- | --- |
|  | **%** | **95% CI lower (%)** | **95% CI upper (%)** | **%** | **95% CI lower (%)** | **95% CI upper (%)** | **%** | **95% CI lower (%)** | **95% CI upper (%)** |
| Service provision to users | 45.1 | 22.1 | 68.1 | 14.4 | 1.8 | 26.9 | 25.3 | 0.13 | 37.6 |
| Administration | 14.2 | -1.9 | 30.5 | 21.1 | 6.5 | 35.7 | 18.7 | 7.7 | 29.7 |
| Continuous medical education | 2.2 | -4.6 | 8.9 | 1.3 | -2.8 | 5.4 | 1.6 | -1.9 | 5.1 |
| Unproductive | 29.4 | 8.4 | 50.4 | 46.9 | 29 | 64.8 | 40.7 | 26.8 | 54.6 |
| Meetings | 1.8 | -4.3 | 7.9 | 0.5 | -2 | 0.03 | 1 | -1.8 | 3.8 |
| Outreach | 7.2 | -4.7 | 19.1 | 15.8 | 2.7 | 28.9 | 12.7 | 3.3 | 22.1 |
