# Supplementary material for: Work time allocation at primary health care level in two regions of Albania
Source: PLoS One. 2022 Oct 26;17(10):e0276184. doi: 10.1371/journal.pone.0276184 (PMC9605026; doi:10.1371/journal.pone.0276184)
Supplement: S3 Table — (DOCX) [file pone.0276184.s003.docx]

**S5 Table. Percentage of overall time allocation (including outreach time) and 95% confidence intervals by doctors at rural and urban settings.**

| **Health worker category** | **Doctors** | | | | | |
| --- | --- | --- | --- | --- | --- | --- |
| **Category** | **Rural health center** | | | **Urban health center** | | |
|  | **%** | **95% CI lower (%)** | **95% CI upper (%)** | **%** | **95% CI lower (%)** | **95% CI upper (%)** |
| Service provision to users | 41.2 | 13.4 | 69 | 54 | 14.1 | 93.9 |
| Administration | 17.3 | -4.1 | 38.7 | 9.7 | -13.9 | 33.4 |
| Continuous Medical Education | 3.5 | -6.9 | 12.9 | 2.8 | -10.4 | 16 |
| Unproductive | 29.3 | 3.5 | 55.1 | 30 | -6.7 | 66.7 |
| Miscellaneous | 5.3 | -7.4 | 17.9 | 0.2 | -3.4 | 3.8 |
| Other | 0 | 0 | 0 | 0 | 0 | 0 |
| Meetings | 3.4 | -6.9 | 13.7 | 3.3 | -10.9 | 17.6 |
