# Supplementary material for: Work time allocation at primary health care level in two regions of Albania
Source: PLoS One. 2022 Oct 26;17(10):e0276184. doi: 10.1371/journal.pone.0276184 (PMC9605026; doi:10.1371/journal.pone.0276184)
Supplement: S4 Table — (DOCX) [file pone.0276184.s004.docx]

**S6 Table. Percentage of overall time allocation (including outreach time) and 95% confidence intervals by nurses at rural and urban settings.**

| **Categories** | **Nurses** | | | | | |
| --- | --- | --- | --- | --- | --- | --- |
|  | **Rural health center** | | | **Rural health post ambulatories** | | |
|  | **%** | **95% CI lower (%)** | **95% CI upper (%)** | **%** | **95% CI lower (%)** | **95% CI upper (%)** |
| Service provision to users | 19.5 | -2.9 | 41.9 | 18.7 | -8.3 | 45.7 |
| Administration | 20.6 | -2.3 | 43.5 | 15.5 | -9.6 | 40.6 |
| Continuous Medical Education | 2.1 | -6 | 10.2 | 0.6 | -4.6 | 5.9 |
| Unproductive | 49 | 20.7 | 77.3 | 50.2 | 15.6 | 84.8 |
| Miscellaneous | 6.6 | -7.4 | 20.6 | 12.9 | -10.3 | 36.1 |
| Other | 1.6 | -5.5 | 8.7 | 1.2 | -6.3 | 8.7 |
| Meetings | 0.6 | -3.8 | 4.9 | 0.9 | -5.6 | 7.4 |
| **Categories** | **Urban health center** | | | **Urban health post ambulatories** | | |
|  | **%** | **95% CI lower (%)** | **95% CI upper (%)** | **%** | **95% CI lower (%)** | **95% CI upper (%)** |
| Service provision to users | 18.8 | -12.5 | 50.1 | 18 | -19.7 | 55.7 |
| Administration | 26.1 | -9 | 61.2 | 28 | -16 | 72 |
| Continuous Medical Education | 1.2 | -7.5 | 9.9 | 0.5 | -6.4 | 7.4 |
| Unproductive | 48.1 | 8.1 | 88.1 | 40.7 | -7.4 | 88.8 |
| Miscellaneous | 4.4 | -12 | 20.8 | 12.5 | -19.9 | 44.9 |
| Other | 0.1 | -2.4 | 2.6 | 0 | 0 | 0 |
| Meetings | 1.2 | -7.5 | 9.9 | 0.3 | -5.1 | 5.7 |
