# Supplementary material for: Work time allocation at primary health care level in two regions of Albania
Source: PLoS One. 2022 Oct 26;17(10):e0276184. doi: 10.1371/journal.pone.0276184 (PMC9605026; doi:10.1371/journal.pone.0276184)
Supplement: S1 Text — (DOCX) [file pone.0276184.s005.docx]

**ANALYSIS OF THE CONTEXT IN VIEW OF THE DEVELOPMENT AND IMPLEMENTATION OF NEW JOB PROFILES AND ROLES FOR FAMILY NURSES IN ALBANIA**

**REPORT**

**February 2020**

Contents

[**I. BACKGROUND 4**](#_Toc31687440)

[**II. OBJECTIVE 5**](#_Toc31687441)

[**III. METHODOLOGY 5**](#_Toc31687442)

[**IV. REPORT 9**](#_Toc31687443)

[**1. POLICY AND LEGAL FRAMEWORK OF THE NURSE PRACTICE IN ALBANIA 9**](#_Toc31687444)

[**2. ANALYSIS OF THE CURRENT PRIMARY HEALTH CARE SECTOR (STRENGTHS, WEAKNESSES, OPPORTUNITIES AND THREATS) 22**](#_Toc31687445)

[**3. HEALTH AND SOCIO-ECONOMIC NEEDS OF THE POPULATION THAT ARE NOT MET BY THE PRESENT PHC AND SOCIAL SERVICES 44**](#_Toc31687446)

[**4. FACTORS, OPPORTUNITIES AND BARRIERS THAT CAN IMPACT THE DEVELOPMENT OF NEW JOB PROFILES AND PROVISION OF A MORE COMPREHENSIVE SET OF PHC SERVICES *(ad-hoc assessments)* 53**](#_Toc31687447)

[**5. CONCLUSIONS 72**](#_Toc31687448)

ABBREVIATIONS

BPPHCS: Basic Package of Primary Health Care Services

BPS: Basic Package of Services

CE: Continuous Education

CME: Continuous Medical Education

CPD: Continuous Professional Development

CPG: Clinical Protocols and Guidelines

CHCIF: Compulsory Health Care Insurance Fund

CTG: Core Technical Group

DCM: Decision of the Council of Ministers

FD: Family Doctor

FHN: Family Health Nurse

FMTS: Faculty of Medical Technical Sciences

GP: General physician

HAP: Health for All Project

HC: Health Center

HP: Health Post

ICN: International Council of Nurses

IMIAS: International Mobility in Aging Study International Mobility in Aging Study

MoHSP: Ministry of Health and Social Protection

MoESY: Ministry of Education, Sports and Youth

MOSHA: Movement of Organization Supporting Healthy Ageing

NCD: Non-Communicable Diseases

NCQSA: National Center for Quality, Safety and Accreditation

NCCME: National Center for Continuous Medical Education

NGO: Non-Governmental Organization

NOHS: National Operator of Health Services

ON:Order of Nurse

PHC: Primary Health Care

PHD:Public Health Directorate

RHA: Regional Health Authorities

RHD:Regional Health Directorate

TAIEX:Technical Assistance and Information Exchange

SWOT: Strengths, Weaknesses, Opportunities, Threats

UNFPA:United Nations Fund for Population Activities

UNICEF: United Nations International Children's Emergency Fund

WHO: World Health Organization

# BACKGROUND

All people, everywhere, deserve the right care, right in their community. This is the fundamental premise of primary health care.

For the first time in history, the population of people aged 60 years and older outnumber the population of children under 5 years. The implications of this shift, in terms of the demands and costs of health care are immense. Economic growth, modernization and urbanization have opened wide the entry point for the spread of unhealthy lifestyles. Instead of diseases vanishing as living conditions improve, socioeconomic progress is actually creating the conditions that favour the rise of non-communicable diseases (NCDs).

Nurses respond to the health needs of people in all settings and throughout the lifespan. Their roles are critical in achieving global mandates such as universal health coverage and the Sustainable Development Goals. The World Health Organization (WHO) continues to act on its commitment to strengthening nursing and the health workforce in general.

“HEALTH21”, the health policy framework for the European Region of WHO introduces a new type of nurse, the Family Health Nurse (FHN), who will make a key contribution within a multi-disciplinary team of health care professionals to attainment of the 21 targets for the twenty-first century set out in that policy.

In many Primary Health Care (PHC) settings, having nurses as full members of the PHC team is essential to meet the complex health and social needs of the populations. PHC service delivery by nurses has been convincingly linked to improved quality of care, efficiency and decreased cost.

The implementation of the family and community nursing services appears to be easier in countries where the national health care system ensures universal coverage and a comprehensive range of health services than where health care systems are predominantly based on health insurance. In countries with experience of public health nurses’ services, all social groups accept them and about 95% of families use them. These positive results would suggest that the role of family health nursing is worth further exploration.

PHC is considered a fundamental service in the Albanian Health System efforts to control diseases and protect population’s health. Based on principles of universal health coverage and in line with the WHO European Framework for Action on Integrated Health Service Delivery, the Government of Albania has prioritized investments in PHC. Investments are planned to be aligned with the design of new PHC model that takes into account peculiarities of urban and rural populations.

The roles of nurses in PHC in Albania remain varied, with a clear distinction between the role in HCs and in HPs. There is also a clear underperformance and under-use of nurses with regard to many of the services outlined in the BPS (Basic Package of Services).

The needs to “*Redefine (strengthen) the role of nurses in the PHC professional teams”* and “*Create a stimulating and motivating environment for professional and responsible teamwork in PHC*”have been formulated in the upcoming national draft “Strategy on the development of primary health care services in Albania, 2020-2025”.

There are planned several actions which aim to fulfil the following expected results: Nurses will have more skills and opportunities to act; Nurses will be more engaged in the multidisciplinary teams (e.g., social workers, psychologists, etc.); Nurse work in communities will be more proactive; PHC professionals will feel supported to provide more quality services; Increased self-esteem and motivation of PHC professionals.

A motivated and task-oriented staff is essential to providing the highest quality of primary health care services. PHC in Albania needs staff distributed according to the community health needs, capable of adapting to the increasingly complex and growing demand for health care services driven by rapid demographic, epidemiological and social change. No reform could achieve its goals without addressing the gap and needs in human resources, in particular of doctors and nurses working in the sector.

# OBJECTIVE

The main objective of this consultancy was to provide context analysis in view of the development and implementation of new job profiles and roles for family nurses in Albania, following these steps:

- Describing and analyzing of the policy and legal framework that regulates the exercise of the nurse practice in Albania.
- Providing a description of current situation of PHC services with all its strengths, weaknesses, opportunities and threats using the existing documents and reports.
- Identifying and describing of the health and to some extent socio-economic needs of population that are not met by present PHC and social services, including epidemiological trends that may impact the structure of the care model and the scope of the nursing role.
- Identifying and describing of the factors, opportunities and barriers that can impact the development of new job profiles and organization and provision of a more comprehensive set of PHC services.

# METHODOLOGY

**The timelogy and Approaches of the consultancy consisted of:**

- Conducting a desk review of all the documents and reports with specific focus on the contextual factors that could affect the development and implementation of the new role of family nurse in Albania.
- Carrying out interviews with key informants from all relevant institutions and stakeholders involved.
- Carrying out focus group discussions with family doctors and nurses from HAP regions (Fier or Dibër) and Tirana, as well as patients attending PHC services.
- Performing an ongoing/continuing consultation and exchanges of the findings with the Core Technical Group (CTG) and HAP team.

***Research protocol***

This project encompassed the following three methodological components:

- *Literature review:* consisting of the current knowledge including substantive findings, as well as theoretical and methodological contributions to a particular topic. Literature reviews are very important secondary sources of information. In the current project, an integrative literature review was conducted with the purpose of generating useful knowledge on the topic or interest through the process of review, critique, and eventually synthesis of the literature under investigation.
- *Key informant interviews:*this consisted of unstructured or semi-structured in-depth interviews with professionals with good knowledge on the topic at hand. The purpose of key informant interviews was the collection of information from a wide range of experts including authorities in the field, top experts and other relevant professionals who have first-hand knowledge about the topic of interest. Key informant interviewing is an important data collection instrument with several strengths, including the following: i) open interviews allow participants to use their own words; ii) open interviews can also focus on several aspects and issues which are deemed rather important by participants, and not only those aspects and issues driven by the researchers; iii) open interviews allow for clarification and further explanations; iv) open interviews provide a unique opportunity for in-depth exploration of different characteristics and phenomena; v) at the same time, an open interview allows for recording of different behaviours and reactions of participants.
- *Focus groups:* which currently constitute a frequent research method in qualitative studies. Nowadays, the use of focus groups method is widespread in the health sector because they provide useful clues that complement individual (in-depth) interviews, or the direct observation of participants.

***Instruments:***

1. a set of key-words (search terms) for the online (electronic) literature review;
2. a semi-structured questionnaire for key informants;
3. a detailed interview guide for focus groups.

***Desk review***

The desk review component consisted of identification of all the existing documents and reports which provide data regarding the policy and legal framework that regulates the nurse practice in Albania, the current situation of PHC services, health care and some socio-economic needs of the population that are not met by the present PHC and social services in the country.

An on line/electronic research was conducted with focus on government published data and other reports and policy documents.

Selected relevant key-words were employed in both Albanian and English languages including (at least) the following search terms: “family nurse”, “family health nurse” “nursing services”, “nursing workforce”, “nurse competencies”, “PHC systems”, or “PHC structure”, etc. All potential databases and relevant materials were carefully searched.

***Selection of key informants in Tirana***

Ten representatives with experience and integrity were selected from different stakeholders. (see the table below)

The key informant interviews included representatives from the MoHSP, CHCIF, NOHS, NCQSA, NCCME, Order of Nurse, NGOs operating in the health field, and the like.

Selected institutions where key informants have been recruited are listed below:

| **Institutions where key informants have been selected** |
| --- |
| 1. Primary Health Care Department, MoHSP |
| 1. Compulsory Health Care Insurance Fund (CHCIF) |
| 1. National Centre for Quality, Safety and Accreditation of Health Institutions (NCQSA) |
| 1. National Operator for Health Services (NOHS) |
| 1. Faculty of Medical Technical Science |
| 1. National Centre for Continuous Medical Education (NCCME) |
| 1. Order of Nurse |
| 1. Health Centers (Policlinics) |
| 1. NGOs operating in the health sector (Together for Life, |
| 1. NGOs operating in the health sector (HAP) |

***Selection of participants for focus group discussions***

This activity consisted of the following focus group discussions:

1. with family doctors from HAP region (Fier or Dibër),
2. with family nurses from HAP region (Fier or Dibër),
3. with family doctors from a Health Care Center in Tirana.
4. with family nurses from a Health Care Center in Tirana
5. with patients from the same Health Care Center in Tirana selected for the points iii. and iv

- For the focus groupdiscussions with doctors and nurses from HAP region, around 8-10 (eight to ten) people were invited to participate in each focus group. They were purposively selected in order to be geographically representative (urban/rural, mountains, hills, plains, etc.), as well as age and gender balanced. The selection process consisted of the following two phases:

- In the first phase, 10 (ten) heath centersof Fierwere identified from the database of all health centres in Albania. The HAP local coordinator were involved during the selection process.
- In the second phase, directors of the health centres selected in the first phase were contacted and consulted about recruitment of the most appropriate participants. The HAP local coordinator was involved also during the second phase of the selection process.

Focus groups lasted about 1.5-2.0 hours.

- For the focus group discussions with doctors and nurses, we proposed to include the Health Center No. 1 in Tirana, which is a very suitable facility for meeting the purpose and objectives of the current consultancy work.
- For the focus group discussions with patients from the above Health Center in Tirana, with the support of health personnel (Director of the health center and the chief-nurse), there were invited at random some primary health care users attending the services in the respective day of the focus-group discussion.

# REPORT

## **POLICY AND LEGAL FRAMEWORK OF THE NURSE PRACTICE IN ALBANIA**

***From legislation to regulation***

Health is a basic human need and a social right, not simply a market commodity. Thiscommitment places significant obligations and responsibilities on governments, which aroundthe world, through their ministries of health and related agencies, have an important role toplay in health care. Their role is in strengthening the health systems as well as thegeneration of human, financial, physical, technical and other resources. These effortssupport health systems to achieve their goals of improving health, addressing access tohealth care, securing adequate financing and responding to their population needs.

Government’s primary role in professional nursing regulation is in establishing appropriatelegislation. Statutory regulation should be designed such that it promotes nursing’s ability torespond to societal needs and supports nursing’s role in health care services and in meetingnational and international health-related objectives^[[1]](#footnote-1)^.

Legislation may be used as a means to empower or constrain nursing practice. Anunderstanding of the processes involved in the preparation of legislation is vital in order tohave real influence over its outcome. Although it may be the lawyers representing thegovernment health department who will be responsible for turning the policy objective intothe necessary legislation, it is essential, however, that identified representatives from thenursing profession work with the legal drafters to ensure that the end result actually meets itsoriginal policy objectives (ICN 2007).

The nursing profession may be regulated through a number of different mechanisms.

Statutes, laws, decrees or ordinances constitute the highest level (for consistency the word

‘legislation’ will be used). Having established the legislation, secondary legislation in theform of rules and/or regulations can be issued. This is followed by the interpretation andimplementation of both the law and the rules and regulations.

- 1. **Legal Framework**

The legal framework that regulates the nursing practice in the Republic of Albania is summarized below.

- **Law No. 10 107, date 30.03.2009 for “Health care in the Republic of Albania”, changed and amended by the Law No. 27/2019, date 08.05.2019^[[2]](#footnote-2)^**

Amendments of the law on health care in the Republic of Albania endorsed in 2019 introduce the *clinical audit*among health care professionals including nursing care, based on clinical guidelines and protocols on health service delivery, as a core prerequisite for improvement of the quality of care and monitoring of the performance of health care services. According to the new law,clinicalguidelines consist of documents which aim at guiding health care professionals for decision-making in their routine clinical practice.

Furthermore, an important amendment of the health care law in 2019 includes the establishment for the first time ever of the portal “Nursing for Albania”, which is an online platform for all potential candidates who wish to pursue a career path and official employment in nursing.

Also, the amended law defines the process of *accreditation* which is an independent process of external evaluation of the quality of health care services delivered in primary health care sector, as well as hospital services.

In addition, the amended law establishes the National Health Council which is a counselling body for development of strategies, policies and reforms in the health care sector. Nevertheless, the current composition of this counselling group does not explicitly specify participation of nurses. The role and mandate of this Council is yet to be assessed in the future.

Along with this, the new law foresees the establishment,for the first time ever, of the National Council of Patients, which constitutes another important counselling body in the context of current health care reforms in Albania.

- **Law No. 10 171, date 22.10.2009 for “Regulated professions in the Republic of Albania”, changed by the Law No. 10 470, date 13.10.2011^[[3]](#footnote-3)^**

All teaching programs delivered by nursing schools in Albania are based on the Law No. 10 171, date 22.10.2009 for “Regulated professions in the Republic of Albania”, changed by the Law No. 10 470, date 13.10.2011.

This law endorses the necessary criteria for the application of several important professions which are concerned with the protection of public interest and health, respecting the principle of independent scrutiny and professional autonomy. In addition, this law aims at warranting and ensuring the necessary standards for several key professions, in order for these professions to be applied by qualified individuals who meet the conditions and criteria foreseen in this law and other laws.

The field of application of this law includes every individual who seeks to apply a certain regulated profession in the Republic of Albania. The nursing profession is one of the regulated professions in the Republic of Albania.

- **The Law on “Nursing Order in the Republic of Albania”^[[4]](#footnote-4)^**

The Law on “Nursing Order in the Republic of Albania”, No. 9718, date 19.04.2007, amended by the Law No. 10462, date 13.09.2011 aims also at regulating the juridical status of several professions including nursing, physiotherapy, midwifery, as well as the organization of the Nursing Order.

In its mission, the Nursing Order collaborates with the Ministry of Health and its related institutions, as well as other public and private organizations. The Nursing Order during its activities interacts closely with the line ministry and other relevant ministries, institutions and organizations, public and private, established according to the current legislation in Albania. The Nursing Order informs every six months the responsible Ministry for Health with regard to its membership including nurses, midwives, physiotherapists, lab technicians, imaging technicians and logopedists. Also, it informs about the disciplinary trials and the related progress in all steps of professional judgments. This type of information is published in the webpage of the Nursing Order.

- **The Statute of the Nursing Order in the Republic of Albania^[[5]](#footnote-5)^**

The mission of the Nursing Order is to safeguard the standards approved by the Minister of Health concerning the professions of nursing, midwifery, physiotherapy, lab technicians, imaging technicians and logopedists through supervision of the norms endorsed in the ethical-deontological code, as well as protection of the patients and public from the malpractice.

In the fulfillment of its mission, the Nursing Order, among other things: i) Collaborates for development of professional standards for planning, design and implementation of professional programs; ii) Implements different training courses for ensuring continuous professional development of nurses, midwives, physiotherapists, lab technicians, imaging technicians and logopedists.

In this framework, the nursing Order contributes in the design of curricula at different levels of education as close as possible with the real needs of family nurses, as well as providing different training courses for nurses nationally.

- **Code of Labor of the Republic of Albania^[[6]](#footnote-6)^**

The Code of Labor of the Republic of Albania is a high profile document based on the Constitution of the Republic of Albania that regulates the relations between employers and employees in Albania. It respects the international conventions ratified by the Republic of Albania and it is based on the generally recognized norms of the International Law. The Code of Labor applies to all the professions, including nursing. It regulates contract of employment, employee’sobligations, employer’sobligations, safety and health protections, workplace, working conditions and loads, payment, termination of work relations, and other aspects.

- **Code of Ethics and Deontology^[[7]](#footnote-7)^**

The Code of Ethics and Deontology contains the values and principles of fair conduct related to the obligations and rights of the professions: nurse, midwife, physiotherapist, laboratory technician, imaging technician, speech therapist (logopedist) in their daily care of human life, health, freedom and dignity.

- **Strategy on the development of Primary Health Care Services in Albania, 2020-2025**

In the “Strategy on the development of primary health care services in Albania, 2020-2025” (draft 4), under the:

Overall objective II: Support the development of sustainable and quality human resources in the field of PHC, the following specific objectives are foreseen:

Specific objective 2.4. Redefine (strengthen) the role of nurses in the PHC professional teamsis expected to be met through interventions related to:

- Review the job descriptions in terms of services provided by nurses at the primary health care level. Nurses will be trained to take on new roles, provide new services and proactively engage at the community level (e.g. management and provision of family care services, prevention of NCDs, etc.).
- Review nurses' job descriptions to include new responsibilities; empower them to have a higher professional profile, including the case of nurses working in health posts in remote areas where no family doctors are working.

*Following these interventions it is expected that:* Nurses will have more skills and opportunities to act;Nurses will be more engaged in the multidisciplinary teams (e.g. social workers, psychologists, etc.);Nurses work in communities will be more proactive.

Another Specific objective (2.5. Create a stimulating and motivating environment for professional and responsible teamwork in PHC**)** is expected to be met through interventions related to**:**

- - Reassess, reformat, and improve PHC professionals' job descriptions (for both family doctors and nurses). Define the appropriate professional skills for various PHC positions and strengthen the nurses' role to perform multiple tasks, with the aim of facilitating the work of physicians and keeping the community cost of services under control. Support teamwork and interdisciplinary work at health centers (delegation of team tasks, mixed groups of doctors and nurses, management teams: physicians, financiers, nurses, etc.).
  - Set up a working environment where stressful factors in the professional routine are avoided or reduced through good management practices, and effective human resources policies. Raise managers' and workers' awareness of the causes and effects of stress. Engage with the staff to build constructive and effective relationships.
  - Set up rules in the institution that help maintain proper work-time balance and provide protection from arbitrariness.
  - Care for the workers' well-being and health through effective health promotion programs and initiatives to reduce personal and peer stress. Develop an open and supportive culture for professionals experiencing stress or other forms of mental health abuse
  - Provide incentives to motivate the staff working in remote and disadvantaged areas. The Ministry of Health and Social Protection will develop a series of policies that will support the equitable distribution of staff and their retention in vulnerable communities across different catchment areas.

***With expected results:***

- PHC professionals will feel supported to provide more quality services
- Increased self-esteem and motivation of PHC professionals

Under the Overall objective III. Provide access to a PHC with adequate and sustainable infrastructure and technology,it is foreseen: Specific objective 3.1. Sustainable strengthening of HC construction infrastructure

*Interventions/actions*

- Draft and approve norms on the construction infrastructure of PHC facilities with the aim of ensuring universal health coverage and equal access to services.
- Rehabilitate health centers according to the above norms, through public investment and donor support. Ministry of Health will continue to implement the rehabilitation program of 300 health centers

According to the MoHSP representative, the rehabilitation of about 300 health centers is expected to be a driving and supporting factor for the staff of these centers in providing more quality care to their community.

- **Decision of the Council of Ministers No. 101, date 4.2.2015 for “Endorsement of the health services’ package in the public primary health care financed from Compulsory Health Care Insurance Fund”**

This DCM (Decision of the Council of Ministers) sets out the characteristics of primary care health services in Health Centers offered in the respective communities:

- Services are located in areas with the highest accessibility for the population of the coverage areaand organized as to be the first point of contact with the healthcare system.
- The services provided are in line with the basic needs of the population (disease diagnosis, treatment, management and prevention as well as health promotion).
- Patients and families are followed by the same healthcare team.
- The services provided are integrated and coordinated with the highest levels of service, providing specialized care as needed.

Among other things, it also determines on average the number of population that covers one HC along with the specific physician and nurse load, where: *on average, each HC serves a population of about 8,000–10,000 (this figure fluctuates significantly in urban and rural areas); with a doctor/patient ratio of about 1 to 2,500 and a nurse/patient ratio of about 1 to 400 population*.

- **Basic Package of Primary Health Care Services**

In Albania there is defined a clear set of services referred to as the “Health benefit package”, which has to be provided close to the place of residence of the patients through the network of PHC centers.

Basic Package of Primary Health Care Services, (BPPHCS) the revised version, is based on the updates covering mainly the chapters on child health care and women’s health care and reproductive health.

This document defines the basic services of PHC, to be provided in every health center (HC) of Albania. It is used by the MoHSP and CHCIF to determine and plan human resources needs, the general budget for PHC, HC equipment and instrument needs, the referral system (from the family physician to the specialist), and the necessary skills and training needs of the PHC staff; it offers a basis for negotiating contracts between CHCIF and HCs and introduce basic PHCs services that are provided by HCs throughout Albania. The basic package of PHC services determines the tasks (skills) of PHC health professionals (separately for GPs and nurses) and what they should be able to do *(on emergency, child care, women’s and reproductive health, elderly health care, mental health care and health promotion/education).*

The implementation ofthe Strategy on the Development of Primary Health Care Services in Albania, 2020-2025, according to the MoHSP representative, is expected to bring new interventions and reflections also to the Basic Package (see above) regarding FHN job descriptions in terms of services provided by them at the primary health care level (including new responsibilities) in response to identified shortcomings regarding the scope of FHN practice and factors affecting the fulfillment of its role in primary health care delivery.

The strategy also foresees that through the fulfillment ofSpecific objective 2: Increase access to PHC diagnostic and treatment services under a revised basic package**,** ensure that:

- Services will be provided closer to patients;
- 75% of cases can be managed at the PHC level. Patients will have more trust PHC services;
- Services will be more cost-effective, unnecessary travel/over-referral to secondary care will be reduced.

While new nursing profiles may include a substantial de facto role expansion for most nurses (and accordingly require substantial capacity building and change management), the BPS seems to provide a flexible framework for developing the preferred nursing role(s), thus allowing for a pragmatic approach to adapting actual profiles of health professionals in PHC without a great need for potentially lengthy adaptations to the legislation and professional regulation^[[8]](#footnote-8)^. Such role expansion also seems to be in line with the new PHC strategy for Albania.

- **Decision of the Council of Ministers no.737, date 5.11.2014 on “Financing of primary health care services from the compulsory health insurance fund”**

This decree defines the financing mechanism of primary health care services stemming from the compulsory health insurance fund. According to this decision, the CHCIF is in charge of contracting all PHC centres based on a specific package of basic health care services delivered in all PHC centres. According to this contract, the CHCIF does not finance services other than those specified and predefined in the contract with PHC centres. The modalities of payment of the package of health services and additional payments for the personnel of PHC centres are approved by the Administrative Council of CHCIF.

- **Decision of the Council of Ministers no. 419, date 4.7.2018 on “Establishment, way of organizing and functioning of the operator of health care services”**

Based on this DCM, the Operator is currently an established structure and its task, inter alia, is to operate throughout the country, in the area of ​​primary health care at regional and municipal level; organize the work for the provision of primary health care services through the operation of health centers/clinics; coordinate the management of human resources in the primary health care system under the legislation in force, and ensure that the functions related to planning, implementation, accounting and financial reporting are carried out in accordance with the legislation in force. The Operator extends its operations throughout the country, at central level through the Central Directorate, at regional level through Regional Directorates and direct service delivery units including local health care units and health centers.

Overall, the establishment of the Operator is expected to strengthen the autonomy, decentralization and decision-making at a local and regional level, as a basic prerequisite for ensuring a prompt response to community needs and better quality of health care services.

- **Decision of the Council of Ministers No. 865, date 24.12.2019 on “Manner of conducting the accreditation process of health care institutions d setting the tariffs and deadlines”**.

This DCM foresees the steps to be taken until full accreditation of the health care institution.

Prior to the accreditation process, public health care institutions must meet the basic standards for accreditation and then proceed with the optimal ones.

The health care institution that has met the basic accreditation standards, following the invitation received from the institution responsible for accreditation, may further pursue the accreditation process.

The institution responsible for accreditation gives the healthcare institution the optimal standards for accreditation, the forms to be completed, and the accompanying documentation for the self-assessment assistance.

- **Quality standards for accreditation of primary health care institutions**

Accreditation standards serve as the key to the accreditation program in Albania. These standards have been reviewed and approved by the working group established by Order of the Ministry of Health and Social Protection No. 630, date 12.29.2017. The system set up for this purpose provides an organized and structured mechanism to identify the best example and weaknesses of health care institutions. The process of external evaluation of institutions in the form of accreditation consists of visits to institutions (health centers) undertaken by a group of authorized accreditation experts. External evaluators' visitis conducted to determine to what extent the institution in question is meeting the standards. The results of the visit are used to determine the status of accreditation.

The Standards for Primary Health Care Institutions are organized into three categories:

1. 33 Basic Standards (15 standards and 18 sub-standards);

2. 35 Optimal Standards (13 standardsand 22 sub-standards);

3. 23 Future Standards (7 standards and 15 sub-standards).

There are 91 standards in total. To be accredited, the primary care institution must meet the following criteria:

1. Must fully meet, i.e. 100%, all **basic standards**.

2. Must meet at least 70% of the **Optimal Standards** score.

3. Must meet at least 40% of **Future Standards** score.

According to the NCQSA representative out of about 420 primary health care centers in the country, only 6 of them have been accredited, while others are in the process.Referring to the NCQSA official website the accredited centers are^[[9]](#footnote-9)^:

1. Health Center “Dispanceria”, Tiranë (Full 5 year accreditation)
2. Health Center “Nr. 4”, Tiranë (Full 5 year accreditation)
3. Health Center “Nr. 10”, Tiranë (Full 5 year accreditation)
4. Health Center “Shijak”, Durrës (Full 5 year accreditation)

Accreditation is considered important by the health center managers as the accreditation confirms a quality and safe care in accordance with the quality standards approved by the Ministry of Health. But, often elements of even basic standards fail to meet for various reasons. As a consequence this non-compliance with the standard regarding the infrastructure of the center requires the support of the MoHSP as well as local authorities such as the Municipality concerned. Failure to meet other standards is also a consequence of the deficiencies of the institution's work, such as having a high level of cleanliness of the premises, having illustrative and informative patient education materials in institution's premises, a program and mechanisms for measuring staff satisfaction, etc.

In the context of measuring staff satisfaction, with regard to the nursing staff, from interviews conducted in the context of this consultancy, no such assessment has ever been carried out either at the health centers or at the Regional Primary Health Care Directorate, or at the level of Ministry. Neither have the evaluations of various NGOs ever taken this into consideration. There are such studies for doctors (but not for nurses, or FHNs)^[[10]](#footnote-10)^.

The set of model documents for accreditation of health centers (see below) provides a model of the organization and tool for this evaluation.

The small number of accredited centers emphasizes the need to foster and support this process not only by procedures, as NCQSA already offers full assistance in this regard, but also by addressing the specific needs of PHC institutions in Infrastructure, Human Resources, Information Management, Security, Patient Rights, Support Services and Quality Improvement.

- **Model document set for accreditation of health centers**

The Ministry of Health and Social Protection declared 2018 as the year of accreditation of health institutions nationally. In this context, the National Center for Quality, Safety and Accreditation encouraged all health institutions to start this process with self-assessment regarding the accreditation standards^[[11]](#footnote-11)^.

The Health for All project, in cooperation with the National Center for Quality, Safety and Accreditation, trained quality teams (self-assessors) from all Health Centers (HC) in Dibër and Fier. The training was followed by a self-assessment which identified in each HC the need for intervention with the aim of meeting quality standards for HC accreditation.

In support of the HC efforts to meet the standards, the Health for All Project mobilized a pool of specialists and experts who were familiar with PHC and the HCs accreditation process to develop this set of model documents. These documents are models that should be adapted by HCs in accordance with their context and as such guide them to implement some standards in the following areas: Infrastructure, Human Resources, Information Management, Security, Patient Rights, Services Support and Quality Improvement.

Adaptation of the contents of this publication should be done with reference to the “Quality Standards for Accreditation of Primary Health Care Institutions” - reviewed and approved by the Ministry of Health and Social Protection Working Group No. 630, Date 12.29.2017. This set can be downloaded online at the official Health for All Project website: www.hap.org.al. All documents in this set can be downloaded in editable format.

- **Decision of the Council of Ministers No. 789, date 22.9.2015 on “Definition of criteria,**

**standards and procedures of the certification program for health professionals** and

**DCM No. 418, date 4.7.2018 on “Some amendments and additions to Decision of the Council of Ministers No. 788, date 22.9.2015 on “Determination of criteria, standards and procedures for the process of accreditation of on-line education activities for health professionals", amended.** and

**Decision of the Council of Ministers No. 66, date 13.2.2019 on “Some amendments to Decision of the Council of Ministers No. 789, date 22.9.2015, on “Definition of criteria, standards and certification procedures for health professionals”.**

There are no specific national accreditation standards for family nurses, but they are general and applying to all health professionals and included in the above DCMs (also confirmed by the representative of the MoHSP, the Order of Nursing and the NCCME). However the regulatory framework (according to the NCCME representative) for continuing education is OK even in response to the FHN figure.

**DCM No. 789, date 22.9.2015** defines the Certification Program for health professionals. Within a 4-year period the professionals involved in the program are required to collect a certain number of continuing education credits, which is 40 credits for nurses. Credits obtained from activities related directly to their specialty must constitute at least 50% of the total number of credits, credits obtained from distance education activities shall constitute no more than 50% of the total number of reported credits and credits obtained from self-reported activities can account for up to 20% of the total number of credits taken by a professional. The rest of the credits needed are determined by the free choice of the professional, based on individual needs.

The professional is encouraged to plan his/her own participation in continuing education activities, based on the purpose of respective professional practice, the identified needs and the desired competencies.

Professionals should be provided with appropriate opportunities and conditions by health structures and institutions to pursue continuing education activities.

All health institutions bear the responsibility to:

- Create conditions and opportunities for professionals to be involved in continuing education activities in accordance with the needs of their job position;
- Promote the inclusion of professionals in continuing education activities and include the requirements of the certification program for professionals in individual employment contracts;
- Develop one-year or multi-year institutional plans for the professional development of their employees, in accordance with individual and institutional needs. Institutions are responsible for providing continuing education activities to their professionals so that they can collect up to 30 percent of the total number of credits required through these activities.

Specific training activities for the family nurse have not been organized by any public or private provider, although part of the credits should be provided by training activities related to their field of practice.

These activities are usually offered on a fee and very rarely paid basis, unlike doctor training that often has outside financial support from different donors. This often makes the participation of nurses in training activities not affordable.

Even health care institutions often fail to fulfil their responsibilities, particularly with regard to providing continuing education activities to their professionals, so that they can collect up to 30 percent of the total number of credits required through these activities. Although the Fund allocates a separate budget for these activities, the centers do not use it. This year this budget has increased to 3-5%. According to the NCCME representative, the HCs are the ideal place to provide on-the-job training as they can deliver them based on the needs assessment of the center's own staff, providing full assistance with application procedures at NCCME premises, there are no application fees, or additional costs for the staff to participate. With the support of HAP it has been possible for the HCs in the regions of Diber and Fier to get support about the way of assessing the training needs of staff, as well as organizing a series of trainings with the new peer-reviewtechnique. HAP also advises HC leaders to pool the budget allocated for continuing staff education to organize trainings when the total HC budget is limited. But usually this budget is used for other needs of the health center.

Another worrying phenomenon is the poor training of rural nurses. There is a tendency of the Health Operator to promote the organization of trainings at district level (as in Korca, Lezhe and Gjirokastra).

The Nursing Order has also provided activities for nurses but the large number of participants (70-100 or more) and the selected referral/lecture technique often make these trainings ineffective.

**For the period 2015-2018** during the monitoring process of the whole process of planning and implementation of continuing education activities by the NCCME (also referred by the NCCME representative) or external evaluations^[[12]](#footnote-12)^,a number of problems were encountered. Some of the issues identified, of interest in our field of analysis, included the large number of participants in the activities; the inability to monitor attendance especially for conference-type activities; pedagogical techniques where referring lecture was the most commonly used one and interactive methods of CEs or practical sessions within training were the least used ones; less training with e-learning or review groups, etc. Nursing education in PHC is weak without any specialization or special trainings; CME (Continuous Medical Education) accredited courses are tailored more to the profile of the associations than to the real needs of health workers, health care institutions and Albanian health system; CME trainings are being carried out in the frame of events, conferences and have too short duration^[[13]](#footnote-13)^.

**DCM No. 418, date 4.7.2018addressed all these issues and made some changes** to strengthen the accreditation criteria of continuing education activities with the aim of enhancing their quality. The new criteria promote the organization of activities with a low number of participants, which are considered more qualitative, allow for interactivity and active participation and have a higher percentage of knowledge acquisition by the participating health professionals.

Thus: For individualized training, which involves acquiring knowledge and skills in performing new procedures or techniques and using technologies with no more than 5 participants, the credit count is increased by the criterion “1.5 credits for every hour ”.

An increase of 0.3 credits per hour can be made on the fulfilment of each of the following criteria: i) the estimated number of participants is up to 25; ii) use of interactive methodology for at least 50 participants; iii) the topic of the activity is in line with the priority areas adopted by the NCCME, where under the FHN (according to the NCCME representative) trainings addressing the topics according to the services and duties set out in the Basic Package are awarded with more credits.”

Meanwhile activities with over 200 participants are discouraged as they apply the criterion “0.5 credits per hour”.

- **Order of the Minister of Health and Social Protection No. 87, date 17/02/2015 on “Evaluation Criteria for Professional Personnel in Health Centers”**

This order sets out the evaluation criteria for the staff: Nurses at Health Centers.

MoHSP has created employment opportunities for all nurses, midwives, laboratory technicians, etc. who have completed higher education or are further qualified in the field of medicine to contribute to community health and this is made possible through the employment portal “Nursing for Albania ”, which has been open since January 2018 and guarantees transparency for every nurse who seeks employment. This new window offers real employment opportunities under a transparent merit assessment system. To date, over 1600 medical technicians (nurses, midwives, laboratory technicians, physiotherapists, x-ray technologists, etc.) have been employed in the hospital and primary care service. But in the view of the representative of the Order of Nursing, giving nurses a preliminary exam to enable them to register on the portal is inappropriate.

- 1. **Definition of the Family Health Nurse**

According to the World Health Organization (WHO, 2000), the Family Health Nurse (FHN)^[[14]](#footnote-14)^:

- Helps individuals and families to cope with illness and chronic disability, or during times of stress, by spending a large part of their time working in patients’ homes and with their families.
- Gives advice on lifestyle and behavioural risk factors, as well as assisting families with matters concerning health.
- Through prompt detection, ensures that the health problems of families are treated at an early stage.
- With their knowledge of public health and social issues and other social agencies, identifies the effects of socioeconomic factors on a family’s health and refer them to the appropriate agency.
- Facilitates the early discharge of people from hospital by providing nursing care at home
- Can act as the liaison between the family and the family health physician, substituting for the physician when the identified needs are more relevant to nursing expertise.

**Family Health Nurse model (WHO, 2000)**


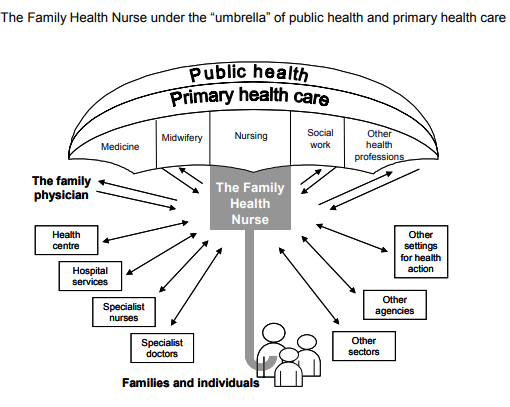


According to WHO, introduction of a new type of nurse that is FHN is vital for the health policy framework of WHO (namely HEALTH21). In this context, FHN is expected to play a key role and unique contribution within a multidisciplinary team of health care professionals (WHO, 2000).

## **ANALYSIS OF THE CURRENT PRIMARY HEALTH CARE SECTOR (STRENGTHS, WEAKNESSES, OPPORTUNITIES AND THREATS)**

PHCin Albania is delivered in health centers, and in the community or at home, and consists of the following types of care:

- Emergency care
- Child care (0-14 years)
- Adult care (15-65 years)
- Women’s health care and reproductive health
- Elderly health care
- Mental health care
- Health Promotion and Education
- Check-up program for the adult population aged 35-70 years (as of 2015 onward)

Based on principles of universal health coverage and in line with the WHO European Framework for Action on Integrated Health Service Delivery^[[15]](#footnote-15)^, the Government of Albania has prioritized investments in primary health care (PHC).

Investments are planned to be aligned with the design of a new PHC model that takes into account peculiarities of urban and rural populations. In order to facilitate the development and implementation of the new PHC model with focus on family medicine, the MoHSPrequested support to WHO in 2018 for a comprehensive assessment of PHC sector in Albania.

An initial assessment exercise was conducted in early 2018^[[16]](#footnote-16)^, which identified several challenges of the current model of care in PHC sector in Albania and proposed areas for improvement and areas which need more comprehensive assessment. As a follow-up to this, a comprehensive PHC assessment was developed and implemented aiming to provide in-depth analysis of primary health care system and identifying the constitutive elements of a new PHC model^[[17]](#footnote-17)^.

Based on this comprehensive assessment, as well as in the other reviewed documents, the main features of the PHC system in Albania are summarized below.

**2.1 THE MAIN FEATURES OF THE PHC SYSTEM IN ALBANIA**

**2.1.1 PHC STRUCTURES**

***PHC governance***

- Until 2019, there was evidence of a high central responsibility of MoHSP for PHC governance.
- As of 2019, there is a unique opportunity to strengthen PHC governance through the newly established National Health Care Operator (NHCO).
- Although national strategies prioritize moving more power and responsibilities to municipalities for PHC administration, construction, rehabilitation and maintenance of the PHC facilities, there are no clear (operational) plans on how municipalities enhance capacities and generate funds for implementation of those strategies.

***PHC financing^[[18]](#footnote-18)^***

- PHC in Albania is financed through annual contracts between PHC centres and CCHIF, which are largely based on historical budgets approved by the MoHSP.
- Salaries of PHC physicians include low capitation segment (0,4-1,1% of total budget) and incentives for geographic areas (5 categories).
- Of note, neither the capitation criterion, nor the geographical distributions are applied to PHC nurses. Therefore, there are no incentives for the nurses operating in the PHC system.
- Until end of 2014, there was in place a “pay for performance” policy. As of 2015, this was abolished. However, theoretically, there is reward in terms of a 13^th^salary at the end of the year for both doctors and nurses, whose application in practice is questionable.
- The average salaries of GPs and PHC paediatricians are only ¾ of the average salaries of narrow professionals and do not support prestige of general practice.
- On a positive note, there is a considerable increase of investments from MoHSP on rehabilitation of buildings since 2018, covering very limited investments during last decade.
- Decreasing co-payments from patients confirm national policy priority on universal health coverage. There are no additional co-payments for PHC services from uninsured population. According to the comprehensive PHC assessment report, co-payments in investigated PHC centres consist of only 0,25% of the total budget in rural areas and 5,8% of the total budget in urban areas.
- As a matter of fact, since January 2017, all PHC services are free-of-charge also for the uninsured population category.

***Quality assurance^[[19]](#footnote-19)^***

- Currently, in Albania, there is well established re-licensing system with compulsory continuous professional development for both doctors and nurses.Professionals must collect a certain number of continuing professional education credits, in accordance with the procedures and criteria approved by the Council of Ministers. Certification is the process that the health professional completes when meeting the criteria of the certification program, which complies with the licensing timeline^[[20]](#footnote-20)^.
- Voluntary accreditation of facilities is encouraged and enforced. However, most of PHC centres are not ready to meet the standards because of poor infrastructure conditions and lack of equipment. As a matter of fact, there are no accredited PHC centres to date, notwithstanding theencouragement of voluntary accreditation of all health centres since 2018.
- National stakeholders report on availability of clinical protocols and guidelines (CPG)for PHC, but only one fourth of health centres apply the CPGs in practice.
- Weak role of Professional Associations, Chambers of Physicians and Nurses and Associations of Patients in the development of CPGs for PHC.
- The clinical performance of PHC nurses is not well supported by CGPs, with exception of guidelines for preventive check-ups.
- The educational counselling package to support nurses in motivational counselling of patients with NCDs and risk factors for NCDs is missing.

***Service coverage and workforce^[[21]](#footnote-21)^***

- There is a well-defined PHC Basic Package of Services (BPS)covered by the CHCIF, which indicates a clear set of services that have to be provided close to the place of residence of patients through the network of PHC institutions.
- There are important differences in capacities to provide the BPS between the main health centres and their affiliated ambulatories (health posts). Some ambulatories (health posts) are serving as branches of main health centres to better reach communities, but provide lower scope of services from BPS, and have GPs visit only once per week, or 1-2 times per month.
- There are no regulatory acts regarding different PHC level (health centres, or their affiliated health ambulatories) in order for everyone to benefit equally and to have the same access to the health benefit package of services.
- The number of doctors per health centre is partly aligned to the size of population served and varies from 1,4 to 6,6 per 10.000 inhabitants, whereas thedistribution of nurses varies to a higher extend (from 7,2 to 34,7), which is difficult to explain by specific health needs.
- There are too many categories of health workers available at health centres, and it leads to fragmentation of PHC services.
- Despite the fact that there is a relatively sufficient number of PHC nurses, their potential to enhance the capacity of people centred PHC and community-based preventive activities is underused.
- The competences of family physicians are established by the MoHSP as a part of the package of basic services.
- No clear job descriptions/profiles for categories of PHC health workers (doctors and nurses).
- There are incentive policies for retention of GPs at remote areas, but they are not applied to PHC nurses.
- Currently, there is no Strategy for Human Resources with a concrete Action Plan and tools for human resource planning.

***Education and CPD***

- No retraining programme for narrow specialists to be retrained into general practice.
- Nursing education in PHC is weak without any specialization or special trainings.
- CME accredited courses are tailored more to the profile of the associations than to the real needs of health workers, health care institutions and Albanian health system.
- CME trainings are being carried out in the framework of specific events, conferences and have too short duration.

***Medicines and information system^[[22]](#footnote-22)^***

- Both formulary and reimbursement list of medicines exist and they are revised annually.
- Only PHC physicians can prescribe reimbursed medicines, but still narrow specialists through compulsory consultations have strong power to induce demand for prescription, which often results in over-prescription especially in case of multi-morbidity.
- High policy priorities to strengthen e-health system through patients registries, e-referrals and e-prescriptions, but weak IT capacities at PHC centres.
- E-patient records are available at specialised out-patient and hospital care, but not available at PHC.
- E-referral system allow to register only to regional narrow specialist, without seeing his name, limits GPs and patients choices of most relevant specialist, in terms of expertise and continuity of specialised care.

***Infrastructure and equipment^[[23]](#footnote-23)^***

- Infrastructure of PHC centers is poor (with exceptions for few urban practices), old and not corresponding to the functional standards of a modern PHC approach (time-hours accessibility, space for consulting activities, working in a team, triage space, etc.).
- The minimal physical infrastructure standards are very general and without clear specifications for premises needed.
- Considerable shortage of diagnostic equipment in the PHC centres, except for check-up program or some procurements that were done in the frame of international projects.
- No imaging technology is present at HCs and ECG is provided only under the National Programme for the Health Check-up.
- Low access of patients to laboratory services especially in rural areas - for simple analyses patients have to go to polyclinics in urban area.
- No attempts to collect blood samples at PHC centres or ambulatories and transport to centralised laboratories with exception for preventive check-ups.

**2.1.2 PHC MODEL**

***Selection of PHC services in alignment with health needs^[[24]](#footnote-24)^***

- Expansive network of PHC centres and ambulatories guarantee good geographic accessibility, and network of facilities was not changed since 2007, when designed.
- PHD/RHA in terms of quantity of public health specialists have potential to better integrate population perspective in PHC and to support PHC teams in population health needs assessment and priority setting, but profile of public health specialists is focused on infectious disease related needs.
- Intensive process in health data collection and reporting, but feedback system and defined priority health needs are more focussed on infectious disease control and vaccination. Data are not used for more comprehensive health needs assessment aiming to inform, select, and adapt PHC services to NCD priority health needs.
- Population stratification is limited to register of patients with NCDs, no stratification by risk for NCDs, no follow up system.
- Counselling services on NCD factors is provided only by preventive check-up nurses, who is well supported by methodological recommendations, but provides only episodic counselling once per year after preventive check-ups.
- Vaccination services are well planned and implemented with support of national programme recommendations.
- Urban PHC centres have rather fragmented paediatric services with PHC paediatricians for preventive check-ups and vaccinations, and other paediatricians, or GPs only for ill children.
- MoHSP reinforce recently implementation at PHC of all three cancer screening programmes (breast, cervical, colorectal) which are not yet well supported with monitoring and re-call system.
- Registered prevalence of main NCDs indicate that early detection of NCDs through opportunistic screening and preventive check-up programmes is still rather low and do not corresponds to what is expected through population based surveys.
- Individual risk assessment, including application of SCORE is provided in check-up rooms, but no follow-up and monitoring of changes takes place.
- PHC physicians have limited prescribing authority for some actual and widely used medicinesby NCD patients, which can be prescribed only with recommendations from narrow specialists.

***PHC design^[[25]](#footnote-25)^***

- Gate-keeping system with compulsory referrals is not respected and self-referrals to narrow specialists areincreasing.
- Referral system limits option of referral only to the nearest hospitals, where often is limited diagnostic capacities. Patients prefer to self-refer to more advanced hospitals, aiming to optimize pathways and to save time.
- Available CGP are limited in clearly defined shared care pathways and referral criteria to narrow specialists. Shared care plans for patients with multi-morbidity and complex needs are not available.

***PHC workforce organization^[[26]](#footnote-26)^***

- Formally population have free choice of PHC professional and PHC centre, but this rule used rarely.
- Every GP has his own patient list and update it regularly, as this is requested by CHCIF.
- All urban and suburban health centres working in two shifts from 7:30 a.m. to 8:00 p.m., but there are some inconsistency in reported working hours from rural health centres and ambulatories.
- Health Centres have to arrange 24/7 services accessible for all population, but they are notequally accessible and even not provided at some rural health centres.

***Service management and quality improvement^[[27]](#footnote-27)^***

- Managers of PHC lack managerial capacity and autonomy in managerial decision.
- Lack of accountability PHC performance related outputs and outcomes to local, regional and/or national authorities.
- Application of internal quality improvement tools was found only in two out of eight health centres, which are pilots of SDC project.

Results of this comprehensive PHC assessment including thesuggested recommendations are meant to inform the design of a feasible PHC model for Albania and development of functional standards of PHC.

Several short-term activities are recommended including the following^[[28]](#footnote-28)^:

- Selection of few districts (regional level) as pilots for revision PHC network and structures and development of PHC model and respective plans.
- Develop criteria and identify best practices and use as:i)pilots for implementation of new PHC model for Albania; ii)training practices, and;iii) demonstration practices for awareness campaigns on PHC vision in Albania targeted to medical society and population.
- Launch population (and medical society) awareness campaigns on benefits of stronger PHC for people health and efficiency of health system (engaging patients, leading narrow specialists and stakeholders from different sectors, as supporters of change).
- Establish incentives for patients to visit PHC (fee for direct visits to narrow specialists).
- Revise scope and frequency of laboratory and diagnostic tests provided during population-based check-ups, leaving only evidence-informed procedures and increase accessibility of the same advanced laboratory and transportation capacities for tests needed for continuous care of patients with NCDs.

Specifically for the nurses, the short-term recommendation is to strengthen the role of nurses, through review of their job descriptions, upgraded competences and provision of more autonomy and responsibility for the following duties/tasks:

1. risk identification, population stratification, follow-up and re-call;
2. counselling on behavioral change and;
3. triage of the patients.
   - 1. **SWOT analysis of the PHC system in Albania**

Based on all the available evidence, the strengths, weakness, opportunities and threats of the Albanian PHC sector are summarized in the following matrix:

**SWOT analysis of PHC services in Albania**

| **STRENGTHS** | **WEAKNESSES** | **OPPORTUNITIES** | **THREATS** |
| --- | --- | --- | --- |
| - Good tradition in PHC sector - Availability of health workforce with basic training - Health centers and posts available all over the country - Basic equipment in place | - OutdatedPHC facilities - Fluctuation in the continuity of care - Poor communication - Insufficient management training - Poor use of healthcare informatics - Lack of sufficient financial resources | - Strong commitment of the government - Upcoming PHC Strategy, Albania 2020-25 - Increased PHC funding - Newly established Health Care Operator - Shift from hospital care to PHC services - Introduction of modern technology | - Political insecurity - Rapid increase of population’s expectations - High turnover of the workforce - Migration of the workforce - Increased demand for (expensive) medical technology - Pressure for cost reductions |

**2.2 PATIENT SATISFACTION WITH PHC SERVICES IN ALBANIA**

Patient satisfaction is important for the quality of services offered in the premises of PHC^[[29]](#footnote-29)^. A recent patient satisfaction survey was carried out in two regions of Albania (Diber and Fier), with a baseline assessment, training and a follow-up (endline) assessment of various aspects^[[30]](#footnote-30)^. The findings about patient satisfaction with various aspects of PHC services and care received is summarised in the following sections:

- Overall high levels of satisfaction were observed. About 70% of patients indicated they were very satisfied and 26% were satisfied with the services received at the day of the survey. About 4% indicated overall that they were very unsatisfied with the services received, whilst the proportion of very unsatisfied patients was substantially higher in Diber (10%) than in Fier (1%). Also, a difference between urban and rural facilities was noticed: patients in urban facilities declare themselves more satisfied that in rural facilities.
- Compared to baseline assessment, endline patient satisfaction on PHC services was higher, especially for services related to antenatal care (although there was a low number of patients participating in both evaluations), and child care. Patient satisfaction at endline varied from 85% to 100%.
- High satisfaction patterns were noticed across various aspects ranging between 80% and 90%. However, only 63% of patients (baseline: 48%) declared that the doctor had asked them whether they were taking any other prescriptions.
- For some items significant differences were found between the regions with markedly lower agreement levels than in the comparative region for three items: patients’ privacy was ensured (Diber 98%; Fier 95%); the medical doctor was polite during consultation (Diber 96%; Fier 99%) and the already mentioned questioning of taking other prescriptions (Diber 70%; Fier 59%). Similarly, there were noticed about three items for which statistically significant differences were observed between rural and urban facilities.
- Differences between rural and urban facilities were often not that big even though some appeared statistically significant (e.g. patient was given the opportunity to explain the health problem:rural 96% vs. urban 91%).

**2.3 CORE FEATURES OF FHN IN THE CONTEXT OF ALBANIA**

Generally, some main characteristics of FHN which has been considered in the current context of Albania include the following:

1. Distribution of nurses
2. Education of nurses
3. Remuneration
4. Participation in decision-making
   - 1. ***Distribution of nurses***

There are different perspectives looking at the distribution of nurses, which are equally important to consider^[[31]](#footnote-31)^: Distribution according to nursing *supply*, to the population *needs*: and to nurses*preferences*.

The table below presents the number of PHC nurses in Albania according to the most recent information provided by the Compulsory Health Care Insurance Fund (CHCIF). The stratification presented in the table represents the 17 Directorates of CHCIF in the country. Overall, there were 6,940 nurses working in PHC services in Albania in November 2019.

**Number of PHC nurses in Albania by district, as of November 2019 *(source: CHCIF)***

| **DISTRICT** | **NUMBER OF PHC NURSES** |
| --- | --- |
| Berat | 475 |
| Diber | 520 |
| Durres | 632 |
| Elbasan | 718 |
| Fier | 373 |
| Lushnje | 269 |
| Gjirokaster | 382 |
| Korce | 432 |
| Pogradec | 163 |
| Kukes | 201 |
| Tropoje | 134 |
| Lezhe | 380 |
| Shkoder | 621 |
| Tirane | 994 |
| Kamez-Vore | 166 |
| Vlore | 350 |
| Sarande | 130 |
| **TOTAL** | ***6,940*** |

There is evidence of an increasing trend in the number of nurses operating at PHC services in Albania, as indicated in the table below displaying the numbers pertinent to the previous year (i.e., 2017). Hence, the overall number of nurses and midwives in 2017 was 5949 (3095 operating in different health centres and 2854 in ambulances/health posts).

**Nurses at PHC services in Albania in 2017 *(source: CHCIF)***

| **INDICATOR** | **Urban + Rural** | **Urban** | **Rural** |
| --- | --- | --- | --- |
| **Nurses/midwifes in Health Centers** | 3095 | 1245 | 1850 |
| **Nurses/midwifes in Ambulances** | 2854 | 317 | 2537 |
| **TOTAL** | ***5949*** | ***1562*** | ***4387*** |

On the other hand, the table below presents the number of PHC nurses by 12 regions (prefectures) of Albania vis-à-vis the population size of each region. According to these figures, the national average was 24 PHC nurses per 10,000 population(or 1 nurse for about 417 population or otherwise stated approximately as the load specified by DCM No. 101, date 4.2.2015 and cited earlier). However, there is evidence of a remarkably low number of PHC nurses in Tirana (13 nurses per 10,000 population or 1 nurse for about 770 population) compared with the other regions (prefectures) of the country. The highest number of PHC nurses is evident in Gjirokaster (62 nurses per 10,000 population), followed by Diber and Kukes (44 nurses per 10,000 population in each region). Besides Tirana which exhibits the lowest number of PHC nurses, most of the other big regions exhibit also low ratios (22 nurses in Durres and Fier; 25 nurses in Vlore; and 26 nurses in Elbasan – all per 10,000 population).

**Number of PHC nurses in Albania by prefectures and their respective populations, as of November 2019 *(source: CHCIF and INSTAT)***

| **PREFECTURE** | **NUMBER OF PHC NURSES** | **POPULATION** | **NURSES / 10,000 POPULATION** |
| --- | --- | --- | --- |
| **Berat** | 475 | 125157 | 38 |
| **Diber** | 520 | 118948 | 44 |
| **Durres** | 632 | 290126 | 22 |
| **Elbasan** | 718 | 274982 | 26 |
| **Fier** | 642 | 294747 | 22 |
| **Gjirokaster** | 382 | 61423 | 62 |
| **Korce** | 595 | 207889 | 29 |
| **Kukes** | 335 | 76594 | 44 |
| **Lezhe** | 380 | 125195 | 30 |
| **Shkoder** | 621 | 202895 | 31 |
| **Tirane** | 1160 | 895160 | 13 |
| **Vlore** | 480 | 189311 | 25 |
| **TOTAL** | ***6,940*** | ***2862427*** | ***24*** |

What is noticeable in the PHC system in Albania is a large number of nurses, but their distribution varies widely by region/prefecture, as well as urban/rural areas.Despite the fact that there is a relatively sufficient number of PHC nurses, their potential to enhance the capacity^[[32]](#footnote-32)^ of people centred PHC and community-based preventive activities is underused.

Another affecting factor is that there are no nursing homes in Albania because the attitude of society toward the ill and sick is that the main caregiver should be the family, and the care is provided at home^[[33]](#footnote-33)^.

The pressure of external and internal migration remains a threat to the future. The situation is rendered even more complex considering the internal and external migration of the workforce in particular of the nurses who may seek more attractive jobs elsewhere (especially in Italy and Germany). Hence, around 3000 nurses according to the representative of the Nursing Order have left the country these last 5 years. But,there is no clear evidence of the distribution between the primary and hospital sectors, or the private and public sectors. Meanwhile, if we look only in recent years^[[34]](#footnote-34)^ the figures are as follows: From June 2017 to December 2018, 932 nurses have left the country for employment abroad. There are 962 nurses for 2019, but there is still no information on how many of them are in primary care.

Of note, an assessment of migration trends among physicians has been already conducted^[[35]](#footnote-35)^, whereas no detailed analysis has been carried out yet for the nurses in order to better understand comprehensively the driving factors for their migration.

Internal migration of nursing staff from rural areas to urban areas, according to the representative of the Nursing Order, has resulted in remote areas still having nursing assistants (staff who have completed a one-year training course in the field of health care) that play the role of nurse but with lower competence. There are about 270 such professionals who renew their license under the Nursing Order each year, because based on their formation and skills the Order has considered the need to renew the license for this annual category.

Whereas if we look at the population loads, the rural areas face a ratio of about 1 FHN/300 population while in the city there is approximately 1 FHN/1300 population (case of Health Center No. 1 with a coverage of 86,000 inhabitants and 65 family nurses available). This load has also been a consequence of the significant increase in the population of the city of Tirana in the last 20 years and the overcrowding of existing health centers. Out of 10 health centers in the 80's, today only one HC has been added to Tirana, so it has 11 HCs (according to Nursing Order).

Another important issue to consider is the fast pace of population aging in Albania which continues unabated. From this perspective, the increase in life expectancy and its resulting aging leading to a considerable epidemiological transition (in line with the demographic transition, i.e., decrease in fertility rate and increase in life expectancy) that Albania is currently undergoing requires the nurses to master their abilities, skills and competencies for a proper and effective control and prevention of NCDs, prompt and effective provision of geriatric care, post-operator care, rehabilitative care, long-term care, as well as all other community needs including also the growing needs for a wide range of social services.

In addition, the substantial decrease in fertility rate is another key factor to consider, leading to a gradual increase in life expectancy but not necessarily in optimal health. As a matter of fact, population aging is associated with an increased presence of NCDs, multimorbidity and comorbidity, a gradual increase in the proportion of older people living alone, older people with activity limitations and inability to access basic health care services. In this framework, there is an urgent need to strengthen community services and provide home-based care for this category, especially in rural areas and remote areas of the country.

Two main models (approaches) exist in the area of nurse practice: *restrictive* approach vs. *permissive* model. The restrictive approach defines and protects professional boundaries, whereas the permissive (or client/patient focused) approach considers client needs as paramount (ICN, 2010).

Furthermore, the actual scope of an individual’s practice is influenced by the context in which they practise; consumers’ health needs; level of competence, education, qualifications and experience of the individual service provider’s policy (ICN, 2010).

In the context of Albania, the scope of work should be substantially expanded for the nurses including new skills, abilities, competencies and responsibilities in line with current progress in health sciences, technological developments and innovations, new approaches in treatment modalities, as well as gradual changes of health needs of the population (such as aging with all its related demands: NCD control, geriatric care, post-operator care, rehabilitative care, long-term care, and palliative care). Today, in the context of community needs (according to the MoHSP representative), there is a growing need for services for adolescents, young people, chronic patients with medical needs in the family, for national minorities such as Roma, Egyptians, etc. Referring to the community covered by its staff, the Director of HC No. 1 highlights the increased needs for community service provision for autistic children and mental health problems encountered particularly in older people (over 65 years), in spite of the significant increase in NCD patients (1 in 7 residents in this center lives with an NCD).

On the other hand, nursing supply is influenced by the scant resources for nursing training, aging of the workforce enrolled in the system, as well as a significant decrease in fertility rates observed in Albania in the past few decades (INSTAT 2019). As a matter of fact, most of these features pertinent to Albania are in line with global trends (ICN, 2010).

On top of this, following the international trends, Albania may experience in the future a drop in the number of aspirant nurses, because of the high competition with other sectors/ professions which are more attractive to the young generation (e.g., IT, business schools, etc.). However, currently, the number of students in the Nursing Schools remains satisfactory.

Of note, the ability of the government to ensure sufficient workforce is limited within an environment which gets continuously more complicated also in Albania, fragmented, as well as technologically oriented, where competition and cost containment are of paramount importance and should be balanced against appropriate access to health services, the accepted standards of care and patients’ safety.

- - 1. ***Nursing education***

Globally, there is an urgent requirement for more skilled nurses. At the same time, there is a need to provide a system to educate teachers. Interventions in nursing education need to be carefully assessed and strategically planned and coordinated.

In most countries, there is a growing concern that the education of nurses is not aligned to the health service delivery needs and that the graduates are not equipped with competencies required to address the rapidly changing health profile of the populations^[[36]](#footnote-36),^^[[37]](#footnote-37),^^[[38]](#footnote-38)^.

Regarding education and training in Albania, the current policy on human resources management, career development and forecasting in the health field lacks effective coordination.

Nursing education is characterized by regular certification (diploma) in Tirana and some other main regions of Albania.

The Albanian Ministry of Health and Social Protection (MoHSP) is currently responsible for the human resource policy development including nurses at large, whereas the National Center for Continuous Professional Education (NCCPE) is in charge of training, needs assessment, and training coordination.

Nursing education programs should respond to the national health agenda, the burden of disease and the entire Albanian society. Although in 2017 there is an average of 2.7 visits per capita per year, the indicator remains lower than in other European countries. PHC in Albania needs staff distributed according to the community health needs, capable of adapting to the increasingly complex and growing demand for health care services driven by rapid demographic, epidemiological and social change. To meet these needs, it is necessary to strength the nursing education by^[[39]](#footnote-39)^:

- 1. harmonization of the teaching programmes among all nursing faculties in basic education (graduate and post-graduate), aligning the nursing curricula to the international/European standards with the focus on practical competencies, abilities and skill-mix approach (TAIEX project), introduction of more PHC subjects in the curriculum to strengthen the capacity of nurses to work autonomously and take larger responsibilities.
  2. harmonization of the content of CME activities to correspond to the training needs.

The Ministry of Health and Social Protection (MoHSP) needs to clearly define nursing competencies (including core abilities that are required for fulfilling the role of family nurse), review and update the job descriptions in terms of services provided by nurses at the PHC level and include also the new responsibilities in the Basic Package of PHC, in order to establish a foundation for new/updated nursing education curriculum at all levels^[[40]](#footnote-40)^.

Undergraduate and especially postgraduate education system in nursing should be reformed. Essentially, the nursing resource policy should become an integral part of the overall public health policy in Albania. Nurses and especially chief-nurses should be offered short training programs, as well as full master programs in family medicine and health management.

Education of nurses at the undergraduate level is already institutionalized in Albania. Universities have also institutionalized different post-graduate training programs such as Master’s in nursing, with an emphasis on specific professional specialties. However, no professional Master in family nursing has been developed and implemented so far. There is a clearly articulated need from the Faculty of Medical Technical Sciences (FMTS) to establish such a program in order to enhance training of this professional category that will be able to respond to the growing needs of an ageing population and NCD-s^[[41]](#footnote-41)^ burden and fulfil the tasks defined by the Basic Package of PHC for the seven areas of service provision.

In general, there is a good and clear legal basis which supports the drafting and implementation of study programs in the Republic of Albania (including the new professional master’s degree programme in Family Nursing, which is in the process of development by the Faculty of Technical Medical Sciences in Tirana with support of HAP.However, the continuous changes in normative acts and other limitations pose difficulties for adaptation of study programs according to the European Framework for Regulated Professionsm(especially in relation to the ratio of teaching hours (theoretical vs. practical hours). In order to meet the legal obligations for adapting bachelor and master programs according to the legal framework and the European directives, the Ministry of Education, Sports and Youth (MoESY) needs to adopt new guidelines for the organization of studies, based on the Directive 2005/36 / EC and Directive 2013/55 / ​​EU of the European Union for study programs in the field of Medical Sciences, which endorse the right to practice the regulated professions^[[42]](#footnote-42)^.

It is very important the harmonization of the teaching programmes among all nursing faculties in basic education (graduate and post-graduate), aligning the nursing curricula to the international/European standards with the focus on practical competencies, abilities and skill-mix approach; introduction of more PHC subjects in the curriculum^[[43]](#footnote-43)^ to enable professional nurses get acquainted with current principles of a comprehensive assessment and monitoring of the health status of the population, as well as identification and implementation of effective programs and measures to protect and promote public’s health, and strengthen their capacity to work autonomously and take larger responsibilities.

There is a need to continuously evaluate and improve the educational environment of the Nursing.

WHO advocates the continuing of nursing and midwifery education as the roles, responsibilities, and scope of practice for nursing and midwifery workforce continues to evolve and expand in line with local, regional and global health challenges (WHO, 2016a,b).

Continuing Professional Development, or the Continuing Education (CE), already represents a norm for healthcare professionals in Albania^[[44]](#footnote-44)^. It is an important prerequisite for successfully fulfilling their mission of providing quality health services in accordance with modern standards.

The inclusion of nurses in the certification program as of January 1, 2016 represents a very important development. For nurses, as for physicians, it is equally important to improve and update their knowledge and skills through continuing education programs. This innovation of the continuing education system will serve the development and strengthening of nursing care, which is of particular importance.In May 2017, the legal framework for the accreditation of providers of continuing education activities for health professionals was adopted, which marks the beginning of a new phase in the accreditation system. By the end of 2018, three experienced providers have been accredited. National Center for Continuing Education (NCCE) plays a periodic oversight, monitoring and advisory role, taking appropriate measures under the applicable legislation.

NCCE has strengthened the accreditation criteria for continuing education activities with the aim of enhancing their quality. The new criteria promote the organization of activities with a low number of participants, which are considered more qualitative, allow for interactivity and active participation and have a higher percentage of knowledge acquisition by the participating health professionals. The requirements for the accreditation of distance education activities have also been strengthened in order to increase their quality.

Nonetheless, the number of well-qualified FHN in Albania is scarce to date. Based on this, there is an urgent need to prepare well-qualified FHN capable of undertaking the new role in line with the WHO recommended role in terms of people-centered PHC provision, with a radical shift from conventional care toward a clear focus on the public/citizens.

Also, another important issue to consider is the standardization of teaching and training programs in nursing with the EU directives.

The overall number of registered nurses at the National Center for Continuous Education is 23,810. Based on the data from 2019, about 8,650 nurses have attended continuous education activities, of whom 7,146 were females and 1,390 were males. Nevertheless, there is no disaggregated information about the job profile or sector of employmentof the trained nurses, which does not allow for an analysis of the training content or training approach meeting the needs of FHNs for continuous professional development.

The Continuing Medical Education (CME) accredited courses are tailored more to the profile of the associations that deliver these courses than to the real needs of health workers, health care institutions and Albanian health system. CME trainings are being carried out in the frame of events, conferences and have too short duration. Nursing education in PHC is weak without any specialization or special training. Hence, to meet these needs, it is necessary for the MoHSP, Compulsory Health Insurance Fund (CHIF) and the National Center for Continuing Education (NCCE) to assess the needs and identify training gaps (education deficits) and strengthen the nursing education by harmonization of the content of CME activities to correspond to the training needs of the practicing PHC nurses, to enrol more national partners and making them interested in planning and providing of CME increasing in this way the quality of CME; to keep 30 percent of professional development within institutions (in alignment with quality improvement, as example peer review groups (in professional development section) and to promote training activities discussing issues considered high priority by PHC professionals.

On a positive note, there is a gradual increase of academically qualified nurses in Albania, albeit not matching the current and especially upcoming demands of the population. Also, some new teaching programs are currently under development, the most remarkable one being the upcoming Master program in FHN.

Overall, a sustainable development of the health system in Albania and its subsequent improvement will be achieved through education and training of highly qualified nurses, as it has been convincingly shown already by the vast international experiences and best practices in many countries. For a successfuleducation and training of programs development and implementation, it is essential to encourage and support the collaboration and partnerships between FMTS and MoES; MoHSP, CHIF, Professional Associations, Order of Doctors and Order of Nurses and CME Center and different international organizations

Albanian experts should be involved in developing and implementing new programs at pre- and especially post-graduate education in family nursing; development of the list of disciplines and specialties; and establishment of a national plan for the continuing professional development of nurses operating at all levels of health care services in Albania.

Also, the upcoming PHC Strategy aims in the next 5 years to:*“Improve basic education programs for PHC professionals”*through the following:

- Adapt the training of PHC professionals by providing them with the opportunity to be trained in conditions similar to the HC daily practice (training of trainers from the selected HC)
- Develop a Masters Program for family and community nurses (within the HAP 2 project)
- Establish a network of University Centers of Excellence in Family Medicine as a support to improve the professional training of physicians and nurses in undergraduate and postgraduate programs in family medicine.

*Implementation of these activities is expected to achieve the following:*

- 70% of general practitioners will be certified as family doctors by 2025 and will have their training at the HC during their basic education program
- Family doctors and nurses will become trainers in basic education for PHC practitioners
- 50 family nurses operating in PHC will have completed the new Master Program in Family and Community Nursing

Another specific objective is to: *“Improve the role of continuing medical education to support critical developments in PHC service delivery”*, through the following means:

- Increase the provision and expand the range of continuing education activities. Adapt the CME curriculum and training activities to real PHC needs (new profiles - e.g. family/palliative care/mental health skills/guidance on risk assessment and control of NCDs and monitoring of NCDs, etc.)
- Support HCs to provide in-service CME training, for example, by informing peer review groups. Encourage and support the creation and operation of mixed peer review groups (doctors and nurses) to provide a further impetus to teamwork and attain more satisfactory results for service users.
- Introduce continuing education norms for PHC managers. The Ministry of Health and Social Protection will develop a combined continuing education system for administrative physicians and managers of PHC Facilities (but not only), aiming at enhancing the management capacity of the health center management teams.
- Mechanisms to generate sustainable funding opportunities for CME training will be elaborated (e.g. lining CME to secondary revenues)
- A minimum number of Health Centers will be promoted in each region for the provision of CME training in each region until the network of University Medical Centers of Family Medicine becomes fully operational.
- Deliver regular training for family doctors on medical emergencies in cooperation with the National Center for Medical Emergency and the new University Center of Family Medicine.

*Expected results:*

- Sustainable and planned Continuing Education programs
- New skills for PHC doctors and nurses
- 50% of centers involved in structured and evidence-based activities of peer review groups.
- A minimum of 2 HCs will be providing CME training in each region by 2025.
  1. ***Remuneration***

Currently, in Albania, salaries of nurses in general are low. However, nurses’ wages resemble the national average. There is no significant difference in the remuneration schemes of FHN with other nurses operating in the hospital sector. Also, there are no gender differences in remuneration schemes. Of note though, FHN salaries are not even lower than those of family physicians, but also incentives for nurses are less attractive compared to physicians.

The particularly low salaries pose a major problem as it discourages the highly qualified nurses, or leads to emigration of the most qualified workforce which is able to find a similar job abroad (in different countries of the European Union).

PHC is funded through contracts with Compulsory CHIF mainly based on historical budget aligned to approve by the MoH PHC structures.

Salaries of PHC physicians include low capitation segment (0,4-1,1% of total budget) and incentives for geographic areas (5 categories). Both elements are not applied to PHC nurses.

No pay for performance component since 2015, while remain reward as 13th salary at the end of year for both doctors and nurses^[[45]](#footnote-45)^.

Normally, each HC has its own budget which is determined by the CHCIF in consultation with the MoHSP. HC also has the secondary revenue for different paid services provided. This revenue is managed by HC itself but with the prior approval of the MoHSP. Conversely, CHCIF monitors/controls the fulfillment of budget lines allocated to the HC.

Until 2015, HC was able to provide financial incentives to the personnel based on certain indicators of performance. These payments were part of the salary budget line, in addition to a part of the secondary revenue generated by the HC. Currently, there is only an extra payment at the end of the year, which is not more than 100% of one salary. It should be emphasized that remuneration is not based on performance of nurses but only on the performance of physicians. According to the CHCIF representative, there no checklists designed for nurses according to the basic package for services which could be translated into performance assessment. However, the CHCIF has developed a supervision form related to all activities of HC and a performance form for physicians (which reflects the number of visits). Exceptions to this include only the nurse in charge of the check-up program and the nurse in charge of injections, who work independently and, therefore, their performances can be assessed. Nonetheless, remuneration of these categories is also similar to the other types of nurses operating in HC.

Even with regard to the provision of 24-hour Emergency Service there are differences in the way the staff are financially treated, as pediatric emergency medical and nursing staff have a payment of 900ALL, while everyone else has the right to receive one day leave for this working day. This is reflected not only with staff dissatisfaction but also with difficulty in managing the work. According to the leaders of the centers, taking a day off during the week brings obstacles to the provision of community service, at least for the chronic patients scheduled for that day. The same compensation is provided for the on-call service near the center on Sunday.

There is a differentiation in the method of payment between family doctors and FHN, as doctors are paid based on the number of residents they cover while the nurses are not. For employment in places outside the country of residence, the doctor is paid with a bonus based on the place of work depending on the geographical area.

There is a difference also in the individual working contract between health centers and nurses, where the article 7, fraction 22, specifies differentiated remunerations from the director of health center: “only for the personnel who has not exceeded unjustifiably the reimbursement plan and has completed the medical check-up workload providing proof on fulfilment of all these tasks and activities”.

There is no refund or it is minimal in relation to the real value for transportation costs to the workplace (often not affordable for the nurses), especially in the rural and remote areas of the country.

Even for HCs that manage to meet and maintain accreditation, there are no financial incentives that will serve as a positive factor to maintain the quality of community health care services, but also for other centers to meet accreditation criteria.

However, the representative of the CHCIF considers it as a strong supporting point the fact that financing and contracting is carried out by the CHCIF and it is always possible to refine the contract elements in order to improve the service. The contract is flexible even for the introduction of service indicators, it is sufficient that they measurable and reliable. In this case, the updating of the nurse contract may be proposed to the CHCIF council, reflecting also the performance indicators of the service.

Hence, it is recommended to^[[46]](#footnote-46)^:

- Revise contracts and payment schemes for PHC centres, changing input based payment to risk adjusted capitation combined with outcome based incentives.
- Revise historical salary based remuneration of PHC doctors and nurses to more flexible options. Regulation from MOH/RHD on composition of multidisciplinary team and ranges of salaries could remain, but should be introduced incentives for achieved performance targets.

Understanding the value system of community nurses and how they respond to economic incentives/disincentives facilitates the development of reward systems likely to be more relevant and strategic^[[47]](#footnote-47)^

- 1. ***Participation in decision-making***

FHN nurses in Albania are not included in the decision-making process. This is partly due to the heritage of the system which is based on a strong physician-oriented tradition, with little voice for the nursing staff. An additional hindering factor concerns the lack of proper education, qualification and training of the nurses operating at different levels of the health care system in Albania.

Hence, current reforms and programs in Albania should consider and enable a significant empowerment of the nurses through their education and training opportunities, their representation in different Boards and Governing Bodies including the MoHSP.

The Ministry has representatives in the National Council of the Nursing Order, whereas nurses are not part of any Boards/Councils near the Ministry (according to the information provided by the representative of the Nursing Order).

MoHSP should also consider the establishment of a specific Unit on Nursing Care(the Nursing Care Unit has operated until 2014 as part of the organization structure of the MoHSP, but subsequently this unit was abolished).

Another important factor is also the wide use of media (for ensuring a large audience) in order to influence current policies and programs in the Albanian health care sector.

Nursing Day (May 12) should be used as an opportunity to communicate with the public about the profession of nursing as a profession that requires great dedication. Nursing is a mission and not just a profession. Publication of an FHN Bulletin would inform the public about the work and activities of the nurse.

The Albanian Nursing Union has just been set up, which aspires to provide better working conditions, higher wages, job security and professional dignity to the nurses both at country level and to the labor force that has emigrated and works frequently without regular employment contracts in response to today's needs and challenges.

There is a need to re-establish the Department of Nursing at the Faculty of Technical and Medical Science near MUT which does not exist since 4 years ago. In this way, the nursing staff will be represented in the decision making regarding the programs that are developed in the field of nursing.

**2.4 CURRENT NURSING ROLE IN ALBANIA**

The tasks/duties of primary health care professionals are established by the basic package of PHC services. The PHC BPS determines the skills of PHC health professionals (separate for GPs and nurses) and what they should be able to do on emergency, child care, women’s health and reproductive health, elderly health care, mental health care and health promotion and education. PHC institutions use the basic package of PHC services as main regulatory act approving health professionals’ competencies, skills and duties. There are no approved job descriptions/profiles of all categories of PHC health workers (doctors and nurses)^[[48]](#footnote-48)^.

The most recent analysis informing an overview of the current roles and functions of nurses working in PHC settings - Health Centers (HC) and Health Posts (HP) concluded that^[[49]](#footnote-49)^:

The roles of nurses in PHC in Albania are varied. Nurses have different roles and scope of practice depending on their area of practice in the HC: in the Family Doctor (FD) consultation room, in special units, at the reception desk and in nurse-led HPs. Overall, nurses reported spending much time on administrative tasks or assisting the FD and less on direct clinical activities. They have a limited health promotion and education role, they have in general no autonomous role and few have a role training other nurses. Nurses working in the HPs reported a greater scope of direct clinical practice with less administrative tasks and a more clinical patient contact role.

Nurses in HC only occasionally perform a limited number of the clinical services outlined in the BPS. Nurses in nurse-led HP may have a broader scope of practice, but given the level of equipment and infrastructure and the local understanding of concepts (“clinical”, “physical assessment”, “palliative care”, etc.) these clinical nursing services are likely to be very basic.

Overall, there is great potential to increase performance of nurses with regard to the following services: health education and health promotion services; preventive, self-management and chronic disease management services; and basic nursing care in the home.Therefore, it is necessary to improve the training of nurses and revise their role so that they can take on new tasks and play a greater role in maintaining and improving the community’s health.

**Role and responsibilities of nurses in the Albanian PHC system**

A context analysis has been recently conducted by a HAP-commissioned consultancy, which involved two international experts (Deschodt& Van Malderen, 2019). According to this work, the following factors should be carefully considered for the new PHC model and the related role of FHN in the context of Albania:

- *Demographic trends:* currently, Albania is characterized by a gradual increase in life expectancy, a sharp decrease in fertility rate, and a continuous aging of the population.
- *Epidemiological transition:* in line with the demographic changes, current epidemiological trends in Albania consist of a consistent decrease of maternal and child mortality and morbidity, decrease of under-nutrition related burden of disease, decrease of infectious diseases, increase of NCDs, increase of multi-morbidity and co-morbidity among the elderly, a considerable increase of mental health problems, increase of cases in need of rehabilitation, long-term care and palliative care.
- *Social trends:* there is convincing evidence of significant lifestyle/behavioural changes in the Albanian population, as well as an increase in patient/citizen expectations in some segments of the society (the most educated and better-off), whereas the disadvantaged and vulnerable groups are quite submissive in this regard which is an obstacle for improvement of the access to quality of health care services. Other social trends are observed with regard to the trust in the health system, system responsiveness, evidence-based practice, transparency, accountability, as well as cultural diversity.
- *Patient safety and quality:* these issues are of paramount importance for an effective and efficient PHC service delivery.
- *Workforce challenges:* shortages, competencies, work-life priorities, mobility/migration, geographical mal-distribution, professional status (prestige).
- *Novel (non-) pharmacological treatments:* limitations of traditional health care delivery.
- *Resource constraints:* limited health care resources.

Some further issues should be taken into consideration when choosing the best model for the implementation of FHN model in Albania^[[50]](#footnote-50)^:

- There is no single correct model of PHC: health systems, and subsystems such as PHC, are shaped by national histories, linked to economics, politics, culture.
- PHC continues to evolve in Western Europe along several different lines and continues to change in the face of old and emerging challenges (e.g.: group practices rather than single practice FMD).
- Skill mix teams: until recently, doctors dominated the PHC health workforce; in recent years more diverse PHC teams have been formed in most European countries, complementing the skills of FDs by those of nurses, pharmacists, auxiliaries.
- New models of Care: the roles of PHC personnel have changed over time. PHC professionals in many European countries are currently undertaking new roles and functions in the delivery of care.

## **HEALTH AND SOCIO-ECONOMIC NEEDS OF THE POPULATION THAT ARE NOT MET BY THE PRESENT PHC AND SOCIAL SERVICES**

During the last decade, there have been an increased number of visits to PHC services driven by increasing health needs, better access to services and changes in health behavior^[[51]](#footnote-51)^. However, although in 2017 there were 7,942,742 visits to PHC facilities with an average of 2.7 visits per capita per year, the indicator remains lower than in other European countries.

PHC in Albania needs staff distributed according to the community health needs, capable of adapting to the increasingly complex and growing demand for health care services driven by rapid demographic, epidemiological and social change. No reform could achieve its goals without addressing the gap and needs in human resources, in particular of doctors and nurses working in the sector.

- It is necessary to improve the training of nurses and revise their role so that they can take on new tasks and play a greater role in maintaining and improving the community’s health.

It is recommended to revise the existing standards and official duties of PHC nurses empowering them to address the basic NCD-related issues and conduct preventive, curative and counselling activities at PHC level.

- There are no nursing homes in Albania because the attitude of society toward the ill and sick is that the main caregiver should be the family, and the care is provided at home^[[52]](#footnote-52)^.
- The roles of nurses in PHC in Albania remain varied, with a clear distinction between the role in HCs and in HPs^[[53]](#footnote-53)^.Nurses in HCsperform a more limited range of services outlined in the BPSand conduct the large majority in the presence of a family doctor in the HC. Home assessments or interventions are rarely done. Nurses in nurse-led HPs may have a broader scope of practice, but given the level of equipment and infrastructure, as well as the local understanding of concepts (“clinical”, “physical assessment”, “palliative care”, etc.), these clinical nursing services are likely to be very basic. HP nurses spend as much time in the HP as in patients’ home. Role descriptions are not always clear to the nurses themselves. There is also a clear underperformance (Activities of nurses and midwives in HCs and HPs Table 1&2^[[54]](#footnote-54)^) and under-use of nurses with regard to many of the services outlined in the BPS, including:
- Clinical services
- Health education and Health Promotion activities
- Preventative and Chronic disease management services
- Basic nursing care in the home

**Challenges related to epidemiologic transition and control of non-communicable diseases**

- Primary health care services in Albania have been traditionally organized to address the mother and child health issues. However, currently, there is a need to reorient these services in order to address the toll of Non-communicable diseases (NCDs)^[[55]](#footnote-55)^.
- Non-communicable diseases constitute the greatest burden for the Albanian health system and the entire Albanian society. They are the most important cause of premature deaths and loss of years of healthy life in our country. This group of diseases has undergone significant growth over the past two decades, a trend that is expected to continue in the future as a result of the increase in the longevity and aging of the Albanian population.
- An important part of these diseases can be managed and controlled at the PHC services in a cost-effective manner without the need for hospitalization or treatment. The largest number of cases registered in the PHC are composed of two groups of diseases: circulatory or cardiovascular diseases (where the overwhelming majority is hypertension) with almost 389,000 cases and endocrine diseases (where the overwhelming majority constitute diabetes) with approximately 135,000 cases.
- It should be noted that some chronic health problems such as hypertension and diabetes, although classified as benign diseases, are often considered as risk factors for other life-threatening circulatory diseases. For this reason, controlling them can prevent both the burden of these diseases in the health system, as well as unhealthy years of life and premature deaths. To be effective, their control must be comprehensive; primary prevention through healthy lifestyle support, early diagnosis and good management through counseling and treatment.
- There is a steady increase in the number of cases treated at the PHC services for hypertension and diabetes in Albania. The basic health screening program for people aged 35-70 has significantly contributed to increasing the identification and follow-up of these two health problems in the PHC. Thus, for 2015 (when this program started) there is an increase of over 30% for diabetes and over 12% for high blood pressure, compared to the previous year (NCD Report 2018). However, the Demographic and Health Survey (ADHS 2018) demonstrates a very high prevalence of uncontrolled hypertension in the population, while a large proportion of individuals with hypertension are still unaware of this condition. Early identification and effective management of hypertension appears to be a primary challenge for PHC in Albania.
- PHC should also be adapted to the specific needs of vulnerable populations (isolated elderly, people with disabilities, Roma community, etc.). They are more exposed to health risks and are less protected from adverse effects of these risks. Currently, PHC services for vulnerable groups are separated from social services. Both services need to be more coordinated and integrated.

Various changes, among which demographic changes (11% of the population was aged 65 years and over in 2011) have naturally led to a clear epidemiological transition in Albania over the past two decades with a significant shift from infectious diseases to non-communicable diseases (NCDs)^[[56]](#footnote-56)^. Albania has thus joined the majority of European countries that face the NCD epidemic as its most important public health challenge.As a matter of fact, there is an enormous increase in the total burden of NCDs in Albania including cardiovascular diseases, cancer, lung and liver diseases, and diabetes^[[57]](#footnote-57)^.

This burden of disease in Albania is caused by a wide range of health determinants, but particularly, due to a high prevalence of high blood pressure, nutritional related risks, smoking, as well as overweight and obesity (IPH, 2019).

Such demographic and epidemiological changes imply that some groups of services offered under the BPSin PHC should be further strengthened and improved and/or updated in order to successfully and effectively address and adapt to these changing demographic and epidemiologic profiles, specifically referring to health service for the elderly, mental health care and most importantly health promotion and education services. With the increasing share of elderly people among the total population the issue of health promotion and education becomes even more relevant in order for it to be sensitive and deliver the target messages appropriately. Aging is often associated with a considerable rate of mental health issues, including depression, anxiety, suicide and serious declining of quality of life due to social, economic and emotional isolation, weakening of family and social support and increased vulnerability and susceptibility to declining mental health^[[58]](#footnote-58)^. Because of the unique needs of elderly population, health promotion for them, as the World Health Organization states, “is not a business as usual”^[[59]](#footnote-59)^. The implications for PHC care and nursing care are:^[[60]](#footnote-60)^

- Use communication processes that rely on heuristics and intuition, instead of focusing on providing large amounts of information processing and thinking.
- Make messages more relevant to older people, addressing their current needs.
- Apply positive messaging about benefits of lifestyle changes for older adults instead of providing information on potential related harms.
- Tailor information messages to specific older people and individual’s characteristics.
- Manage emotional distress in order to encourage behavioural change and maintenance of that change among older people.
- Consider an older person’s social support and how it can facilitate or sustain beneficial behaviour change.

In a recent limited evaluation of nursing activities in Albania among the activities rated as “feel most competent to conduct” no one of them included care, mental health care and/or health promotion and education for the elderly population^[[61]](#footnote-61)^.But, during the focus group discussions of nurses in the two districts of Tirana and Fier, organized in the context of this analysis, FHNs have also highlighted the need to address the ever-increasing cases of mental health problems, especially in people over 65 years of age.

With regard to palliative care, although the coverage with such services is being gradually expanded over time (there are four palliative care services operating in the country and palliative care unitsare established in most regional hospitals with inpatient and home care services for patients), the needs are not met: home care services are offered only for the cities where regional hospitals are situated, the 12 palliative home care services’ providers currently operating cover only one-third of population need for palliative care. Sporadic capacity building activities for general practitioners and nursing staff in palliative care services have been undertaken in several cities in Albania, but these activities are heavily dependent on donor funding. Therefore, only a very limited number of GPs and nurses have been trained in these topics. Palliative care is part of the curricula in the Faculty of Medicine, Faculty of Social Sciences and public nursing high school but the GP specialization course does not include this topic; in addition, there are four books on palliative care available to the students^[[62]](#footnote-62)^.

The FTMS opened last year Professional Master Degree program with a profile in “Palliative Care”, designed and implemented by the academic staff of this faculty (although there are many theoretical disciplines in this master program and no specific subjects on nursing care).

Nonetheless, challenges ahead are as follows^[[63]](#footnote-63)^:

- implement the new law on palliative care,
- establish palliative care units in all regions of Albania,
- expand the provision of palliative care services to other cities throughout Albania,
- advocate for resources to improve the standards for offering the care in the population,
- establish palliative care for children,
- expand palliative care to other non-cancer patients,
- expand the number of qualified professionals through postgraduate palliative care specializationcourses,
- continue palliative care capacity building and training activities, and,
- promote palliative care through public awareness raising activities.

**Unmet needs for mental health services in the Albanian population**

According to the Albanian Demographic and Health Survey 2017-18 (ADHS 2017-18), the prevalence of depression among men and women aged 15-49 years was significantly higher among poor individuals compared to their wealthier counterparts. This finding was evident in both men and women.

On another issue, there is evidence of a discrepancy between utilization of services and population prevalence of mental health problems in Albania. Hence, the rate of hospital admissions for mental health problems in Albania is only 0.9 per 100,000 population, a figure which is much lower than all the neighbouring countries and beyond (e.g., in Romania and Germany these rates are 13 and 12 cases per 100,000 population, respectively) [IMIAS, 2016].

Likewise, the rate of mental health visits to outpatient clinics in Albania is only 12 per 100,000 population, which is also one of the lowest in the European Region (the highest being in Slovakia with 282 visits per 100,000 population) [IMIAS, 2016).

The figure below presents the observed trend in mental health conditions managed at PHC level in Albania from 2006-2016. There has been a gradual increase, but much more evident in 2015-16 which coincides with the introduction of the national medical check-up program pertinent to all Albanian citizens aged 35-70 years. Of note, major depression constitutes about one-third of the overall cases with mental disorders in Albania.

**Trends in mental diseases (ICD9 290-319) at primary health care level**

**(major depression one third of the cases) [source: IPH, 2017]**


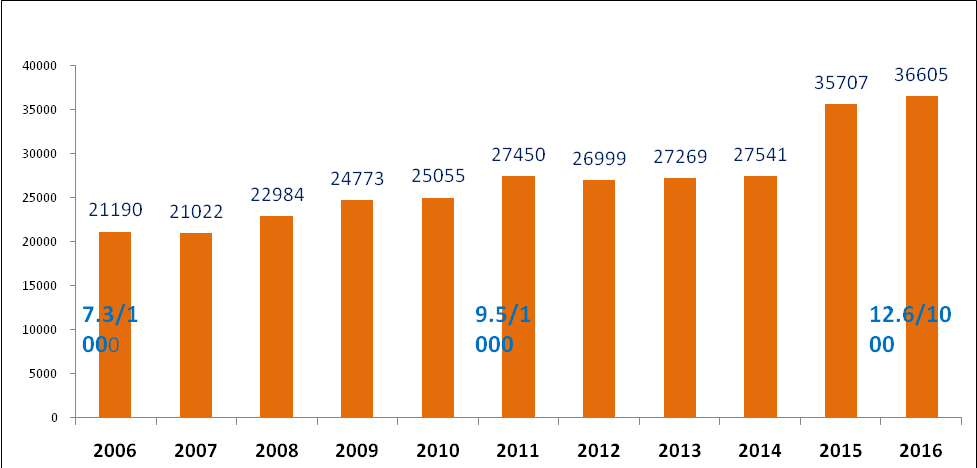


In addition, population-based studies of depression conducted in Albania suggest a relatively high prevalence of depression (IMIAS study). For example, in 2016, IMIAS reported an overall prevalence of depression 24.6%, with a remarkable sex-difference (in women it was twice as high as in men: 32.4% vs. 16.1%, respectively) [IMIAS 2016].

Also, the Youth Risky Behaviour Survey (YRBS) conducted by the IPH in 2009 reported that almost one in three females felt hopeless for two weeks, and about 9% of them seriously considered committing suicide.

The mental health score (using the WHO-5 index) including a representative nationwide sample of people over 65years, according to the 2017 study conducted by MOSHA, exhibited a poor mental health among this population: the overall score was about 42 in a scale from 0 (worst mental health) to 100 (optimal mental health) [MOSHA, 2017].

**Unmet health and social needs of elderly people in Albania**

Cause-specific morbidity among older people in Albania is mainly recorded in the national NCD registers (which is available from the Institute of Public Health).

The table below presents the distribution of the most frequent medical conditions among individuals aged 70 years and above in 2018.

**Most frequent diseases and conditions among individuals aged 70 years and above in 2018 (incidence rate: cases/100,000)**

| **DISEASE** | **Men** | **Women** | **Total** |
| --- | --- | --- | --- |
| **Cancers** | 832.7 | 417.8 | 616.8 |
| **Lung cancer** | 173.1 | 35.1 | 101.3 |
| **Breast cancer** | 0.0 | 50.5 | 0.0 |
| **Colo-rectal cancer** | 48.4 | 21.9 | 34.6 |
| **Cervical cancer** | 0.0 | 9.5 | 0.0 |
| **Heart Attack** | 727.9 | 468.2 | 592.8 |
| **Stroke** | 1066.1 | 867.7 | 924.8 |
| **Diabetes*** | 201.6 | 218.8 | 210.5 |

*Tirana region only.

The elderly people constitute a population category characterised by an excess mortality in both sexes, especially for such chronic conditions as cardiovascular diseases and cancer. Furthermore, chronic respiratory diseases and neurological disorders constitute another significant share of mortality toll among older people in Albania.

Regarding the cause-specific morbidity among the elderly, diseases of the circulatory system constituted the largest share in 2018, followed by diseases of metabolism and the endocrine systemand next by diseases of the respiratory system and the nervous system.

***Health and social surveys among older people in Albania***

Population-based information on elderly people morbidity and general health status is scarce for Albania. Besides IMIAS study, two other nationwide population-based surveys were conducted in this regard by MOSHA (a network of a wide range of NGOs providing services and care for older people in Albania). One of these studies (first round) was conducted in 2008 and the other one was carried out in 2017 (second round). Both surveys employed a similar methodology including data collection instruments.

The cross-sectional study conducted in June-September 2017 included a representative sample of older men and women in urban areas, periurban areas and rural areas of three key districts of Albania (Tirana, Shkodra and Vlora). Overall, 505 (51.8%) women and 469 (48.2%) men were included in the survey. On the whole, 493 (50.6%) participants were aged 60-70 years; 329 (33.8%) individuals were aged 71-80 years; and 152 (15.6%) individuals were aged 81 years and above.

Regarding self-perceived poverty, overall, about 37% of older people reported to be poor compared with 63% of their counterparts who did not perceive themselves as poor.

The prevalence of self-perceived poor general health status was about 36%.

The prevalence of at least one chronic disease or condition was about 47%.

Among older people who reported to have at least one chronic condition, the prevalence of cardiovascular diseases was 65.4%; the prevalence of diabetes was 24.6%; the prevalence of diseases of the stomach and/or the liver was 31.5%; and the prevalence of chronic lung diseases was 36.5%.

Regarding medical care, overall, about 16% of older people reported having not been able to access medical care/services when needed. The inability to access medical care when needed was higher in women than in men (17% vs. 14%, respectively).

The inability for accessing medical care when needed was linearly and positively associated with the age of older people, and it was higher among older people residing in periurban areas (about 25%) compared with their counterparts living in rural areas (16%) and especially those residing in urban areas (around 14%).

Among individuals who reported that they could not access medical services when needed, the following reasons were stated as the main hindering factors: difficulties to afford medical treatment (17.6%), followed by the long distance to medical facilities (12.3%) and the inability to attend medical services due to the presence of severe illnesses/pains (11.4%).

Among participants who were prescribed medications by their respective family physicians, 24.7% of them reported that they could not receive such drugs mainly due the fact that they could not afford them.

Furthermore, the level of inability to pay for drugs prescribed by the medical personnel was higher among older people residing in rural areas and periurban areas compared with their counterparts living in urban areas.

**Long-term care in Albania**

Traditionally, long-term care has been shared between two separate sectors, health and social sectors. As of 2017, both social and health services are coordinated within the same MoHSP. Seemingly, the merging of the two formerly separate ministries (health & social affairs) may provide synergies and better coordination and a more adequate response to elderly care.

However, to date, there is no formal (institutionalized) long-term care in Albania. Instead, long-term care consists merely of some dedicated (“earmarked”) hospital beds, which provide health care mainly for chronically ill older people. Yet, the services provided are not adequate and there are very few physicians who have received specific training on geriatrics and gerontology (MoH, 2016).

Current reform in the health sector aims at allocating more hospitals beds for the elderly. Furthermore, an important achievement relates to the palliative care provided in Tirana and some other major urban areas of the country. Also, home-based palliative services are available in Tirana and some other major urban areas, which do not actually meet the population needs. Hence, close family members and other relatives largely provide palliative care at home.

In the absence of regulated and institutionalised long-term care for older people, the data on its functioning and related cost for these services are scant.

On the other hand, there are no home-based social services for older people in Albania, putting enormous pressure on close family members who should provide support for their older relatives. As a matter of fact, there are several asylums (residential facilities mainly providing shelter and food for the elderly) available, but nevertheless, they provide inadequate services, and more importantly, do not cover the population needs for social care and support of older people.

In this context, long-term care expenditure for health services, especially for social services, is provided by close family members; including remittances from emigrants. However, the traditionally strong family bonds are currently fading away, including the provision of remittances. Therefore, the role of public services becomes even more relevant.

Currently, there is no link between the social insurance systems and social and healthcare provided by the active workforce to their elderly family members who need continuous assistance and daily care. There is no financial assistance provided to the families who provide care and support to their older relatives, except for the poor individuals, who receive an economic aid and electricity bursary. Yet, these small amounts of benefits are likely to have only limited effects on improvement of social conditions and well-being. In addition, there are disability benefits for older people under the recognised (medically certified) disability status. Conversely, regardless of the socioeconomic status and/or disability conditions, basic medicines are reimbursed for all individuals aged 65 years and above. In brief, the pivotal role of both central government and local government for supporting long-term care in Albania is inadequate. Several NGOs try to fill this gap including some religious associations.

## **FACTORS, OPPORTUNITIES AND BARRIERS THAT CAN IMPACT THE DEVELOPMENT OF NEW JOB PROFILES AND PROVISION OF A MORE COMPREHENSIVE SET OF PHC SERVICES *(ad-hoc assessments)***

1. **HR related aspects and specific requirements to exercise new roles; specific areas where nurses feel confident and less confident in their professional role in the community**

**HR related aspects and specific requirements to exercise new roles**

- In primary health care there are needed new nurse skills and responsibilities as:
- There is a change in the health needs of the Albanian population as a result of the increasing life expectancy, and an increase in the number of elderly people, patients with NCDs (especially HBP, diabetes, chronic pulmonary diseases), chronic patients with family medical assistance needs, the elderly living alone at home and patients who are away from the HC, persons with mental health problems particularly encountered beyond the age of 60 years, and number of autistic children. Health professionals also face new health needs as a result of health risk behaviors among youth and adolescents (especially drug use) and the need to provide community service to Roma and Egyptians.
- To meet these health needs of the population today, there must be provided services to the individual and community, not only in health institutions but also at home.
- The PHC nurse working in urban areas has generally turned to a physician’s assistant/secretary for completing administrative documentation while less and less exercising his or her duties of providing direct care (with the exception of some nursing profiles, such as nurses of check up, surgery and obstetrics-gynaecology, according to the representative of HC No. 1). While the role model of the nurse in rural areas is broader due to the lack of a full-time doctor in that particular HC (one doctor covers 2 villages), as he/she has more flexibility (autonomy) and covers more than just one function (vaccination, family visits, women's health, etc.). Therefore, the role of the nurse should be transformed and enriched as to include community engagement as well.
- Recommendations for the new role should be differentiated according to the local context, separately for Tirana and the big cities) and small cities, separately for urban areas and for rural areas.
- To have a realistic and applicable role of the community nurse, the design of this role must rely on available human resources, as big changes take time. Therefore, the aim is to add this commitment to the family nurse, not to create a special role, but to create a more comprehensive profile by applying the nursing skills to the whole environment of the area or community the nurse covers.
- Rural areas have fewer nurses than urban areas, there is a need to substantially strengthen nurses who serve alone in HPs, without a doctor.
- The basic services package has tasks assigned to nurses at PHC, but they are not properly aware of this, and do not know where their responsibilities begin and end. There is an overlap of competencies with doctors for chronic diseases, palliative care, mental health, etc. The duties of the nurse are not clearly defined in the package.
- Until now the nurses do not use all the professional potential their possesses, they are less supported by the system and do not feel motivated at work because the salary is low. It is necessary to change the mindset of the physician towards the role of the nurse and this will make the nurse perform the tasks assigned to him/her in the package. Improving the role of the nurse will lead to not only change the HC (strengthening the physician and nurse team) and better collaboration with multidisciplinary teams such as nurses, physicians, psychologists, social workers, but it will bring changes at system level as well.
- There is a misconception about the figure of the nurse from the perspectives of patient, society, and decision makers, and this must be changed.
- In the context where doctors are having a large workload (registers, forms, cards, health booklets, Medico-Legal Commission, multiple reports required by the various institutions on which the institution depends) this burden is passed on to the nurses, thereby gradually limiting and diminishing their competencies in nursing practice.
- The nurse should cover the home care for the patient in need of such a service, by designing in advance with the physician a patient follow-up plan.
- By providing home/household care the nurse takes care of the basic needs of elderly patients (ensures that the patient maintains acceptable levels of personal hygiene, has a clean bed and receives proper nutrition).
- Exercising the New Role of Nurse makes it possible to reduce the cost of hospital stays (shorter hospitalization), providing more affordable, appropriate and focused care to patients in community.
- Exercising the New Role of Nurse enables him/her to provide a watchful patient care by administering medication (intravenous/muscular, etc.), observing and monitoring the patient's condition, keeping records and communicating with physicians.
- The development of technology, the introduction of many online electronic services, and consequently the reduction of bureaucratic time in function of increasing the time of health care delivery, (in the opinion of physicians) is making the nurse less necessary. If there is a reorganization of the work in the HC later, 3-4 family doctors can meet their needs with a joint nurse, reducing the number of nurses in the outpatient clinic. There are foreign models that engage a family doctor without a nurse, so it is also important for the nurse him/herself to engage with family service in order to become valuable to community health by providing community residents with health care, preventive care, intervention and health education.
- The family nurse should maintain a close relationship with the community school nurse/ physician/psychologist by supporting them with community assessment data and analysis, as well as developing action plans and establishing procedures.
- In the circumstances that nurses are working today, they are less professional. The scope of nursing care in the nursing field of practice should be expanded. The work the nurse does today can be performed by anyone who learns to fulfil some codes or various generalities. Sometimes the doctor does not allow the nurse to perform manipulations such as arterial blood pressure measurement or offering home service alone. Therefore, the focus of the nurses' work should be on nursing care.
- The volume of nurses’ work is undocumented, often doing physician-related work in terms of completing the documentation and facing an overload.
- For some services the doctor is paid while the nurse is not (24-hour emergency service).
- Nurses will be trained to take on new roles, provide new services and proactively engage at the community in concordance with the Definition of the Family Health Nurse (WHO 2000) as well.

**Specific areas where nurses feel confident and less confident in their professional role**

**in the community**

- The most structured service that completely fulfills nursing’ duties child care (including  immunization). This structure has started earlier and it is monitored at all levels. Each immunization center is equipped with the relevant materials. The centers have a refrigerator, the cold chain can be tracked and the child is screened before and after vaccination.
- Check-up nurses are more competent as they are more trained, they offer various services in their day-to-day practice to healthy patients ranging from manipulative procedures to counselling, and they work independently as well.
- In some health centers where the HC manager has separated the physician's room apart from that of the nurse and the nurse has been the first point of contact with the patient, it has been noted that performance is more effective as the nurse succeeds in performing his duties better as a consultant, and performs manipulations with patients such as blood pressure, weight, length, etc.
- Nurses in the village perform their duties better in nursing care since working alone he/she takes on more responsibility, has more autonomy with regard to interventions, he/she provides more home service, patient health education and builds better relationships with them. (*When I was working alone (solo) as a FHN in my village I carried out all the tasks and after a while I felt able and confident in my actions, but after the doctor came he/she did not allow me to offer community services anymore and my skills have begun to fade away”*-refers a nurse).
- Nurses in the city (Tirana) feel more competent in fulfilling secretarial, administrative duties, completion of documentation and chronic illness management (but not in all cases, also due to lack of infrastructure and material basis); in the use and evaluation of growth curves in children; in the management of emergencies of chronic patients such as hypertonic, diabetic or asthmatic attacks, and acute emergencies, such as lipotimia, burns, bites, poisonings. (*While the doctor fills out the prescription, I advise the patient about nutrition or physical activity or provide education about diabetes or hypertension*” - refers a nurse).
- The work performance of the nurses also depend on how collaborative is the patient. (NGO representative)
- Regarding areas where they feel less competent, nurses (Tirana) feel less competent, in performing manipulations such as paediatric intravenous, catheter placement, etc. as they lack experience in applying the practical nursing part. Even the administrative load does not allow for time to deal with manipulation.
- Management difficulties are also encountered in cases of patients who have consumed narcotic drugs. There is a lack of training and experience for such situations, the number of which is increasing. Even relatives are often not collaborative in such cases, especially when patients are minors.
- Some nurses (Tirana) feel able to communicate warmly with patients with mental health problems, and because they feel comfortable in this support, they often visit the health center, but are unsure of properly addressing their needs. They said that they also need cooperation with a psychologist as well as training in this field. Sometimes the communication with their relatives also is very difficult (Fieri).
- In the case of traumas/accidents, they do not feel able to act according to appropriate practices, as practical training on topics such as collar placement or barrel placement and patient transportation has been lacking (especially nurses serving at 24-hour emergency centers).
- Many uncertainties are also encountered in the management of cases of convulsions, especially epileptic seizures.
- The nurses (Fier) report that they fail to properly treat injuries and wounds due to lack of proper training on the nature of the wounds and the way they are treated, but also because of lack of tools for manipulation. While in Tirana this service is not provided by family nurses, as they do not consider it part of their duty with the justification that the center has a special microsurgery service.
- Home care has not been practiced or practiced very little by nurses (with the presence of a physician) not only due to lack of time, but also because the family member does not trust the nurse and usually requires a physician for the service, or the reason of safety (According to a doctor*: The nurse cannot go alone to home visit, as she is not safe in going alone. I can't send an unaccompanied young girl (the nurse) because I don't know who that patient is, number of maniacs is increasing and problems may arise).*
- The follow-up of patients with chronic diseases at home premises is difficult to be done consistently, as there are many tasks to be accomplished within the day. But, in case of need/emergency the nurses have shared their contacts and the patient communicates by phone in case of necessity.
- Nurses face problems in fulfilling their duties also due to the specific demographic characteristics of the service area. So in Fier the nurses also find it difficult to vaccinate Roma children according to the immunization scheme, as they are always on the move (itinerant community).
- Village nurses often find their job difficult to manage, especially in emergency situations, due to lack of training but also due to lack in case of sharing of experiences as he/she works alone. (*Nurses at HPs previously carried out their function without knowing what they were doing. We had a case of a nurse that has been assessed regarding her training need and after training she did more work following up the patients* - refers a doctor who is also the head of the health center).
- They also feel insecure in manipulations such as the placement of the drainage tube (I have had no patients to practice this – refers a nurse), *urinary catheter placement, placement of the adult vygon in young children (since paediatric vygons are not the list of reimbursable medicines*-refers a nurse)*.*
- In Fier, with the support of HAP, nurses are provided with the necessary tools for manipulation but refer training needs, especially for decubitus wounds.
- Nursing home care is not planned but it only done when the patient requires it, and the physician give the permission to the nurse to accomplish this duty (so the nurses cannot go in all cases of need). In rare cases there is planning and this is mainly done by the village nurse (*I do homecare visits once a week* - reports one of them)*.*

1. **Decision-making processes that can impact the structure of the model or the scope of practice of the family nurse**

The following principles may be considered as the basis for making decisions with regard to the scope of practice for an individual family nurse^[[64]](#footnote-64)^:

**• The activity is consistent with the nursing legislation, board policy and guidelines.**

Overall, the development of new nursing profiles seems to be quite feasible. The legal and regulatory framework offers no barriers to a more autonomous role for nurses, and there is some readiness by the stakeholders towards the implementation of new nursing profiles within the existing BPS framework. While new nursing profiles may include a substantial de facto role expansion for most nurses (and accordingly require substantial capacity building and change management), the BPS seems to provide a flexible framework for developing the preferred nursing role(s), thus allowing for a pragmatic approach to adapting actual profiles of health professionals in PHC without a great need for potentially lengthy adaptations to the legislation and professional regulation. Such role expansion also seems to be in line with the new PHC strategy for Albania.

• **The primary motivation for undertaking this activity is to meet patient needs and improve health outcomes.**

In the Albanian context this activity should respond to the country’s national health agenda, the burden of disease, (especially the prevention and control of NCDs) ageing population, most vulnerable population groups and the entire Albanian society.

• **The activity is appropriately authorised by a valid order/protocol and in accordance with established policies and procedures.**

The draft PHC strategy which places the emphasis on nursing, is an important factor in paving the legal path for enhancing the role of FHN.

In the Basic Package of Services which defines the FHN tasks, new tasks are expected to be reflected and updated, clearly defined, in accordance with the expanded role of FHN.

Also, the individual employment contract between the HC and nurse (Figure below) is based on the provisions of the Code of Labor of the Republic of Albania and on the labor norms set by the MoHSP. The fund contracts with the HC and the center itself can tailor individual employee contracts. Article 6 of the Contract on “Job-specific obligations designated by the HC Director allows for a detailed job description, which can be revised and updated in accordance with the new role of the FHN. In the areas where HAP has intervened, individualized job description contract models have been developed.

Some interventions in the contract may enable the evaluation of nurse’s performance. Service indicators to be reviewed and defined (according to the tasks defined in the BPSPH) and patient confidence in the nurse to be measured. The contract is regulated by the Fund's Administrative Council. This intervention will facilitate the performance evaluation by the HC manager, and may serve as a starting point and incentive for implementing quality management systems for nursing services that do not yet exist.


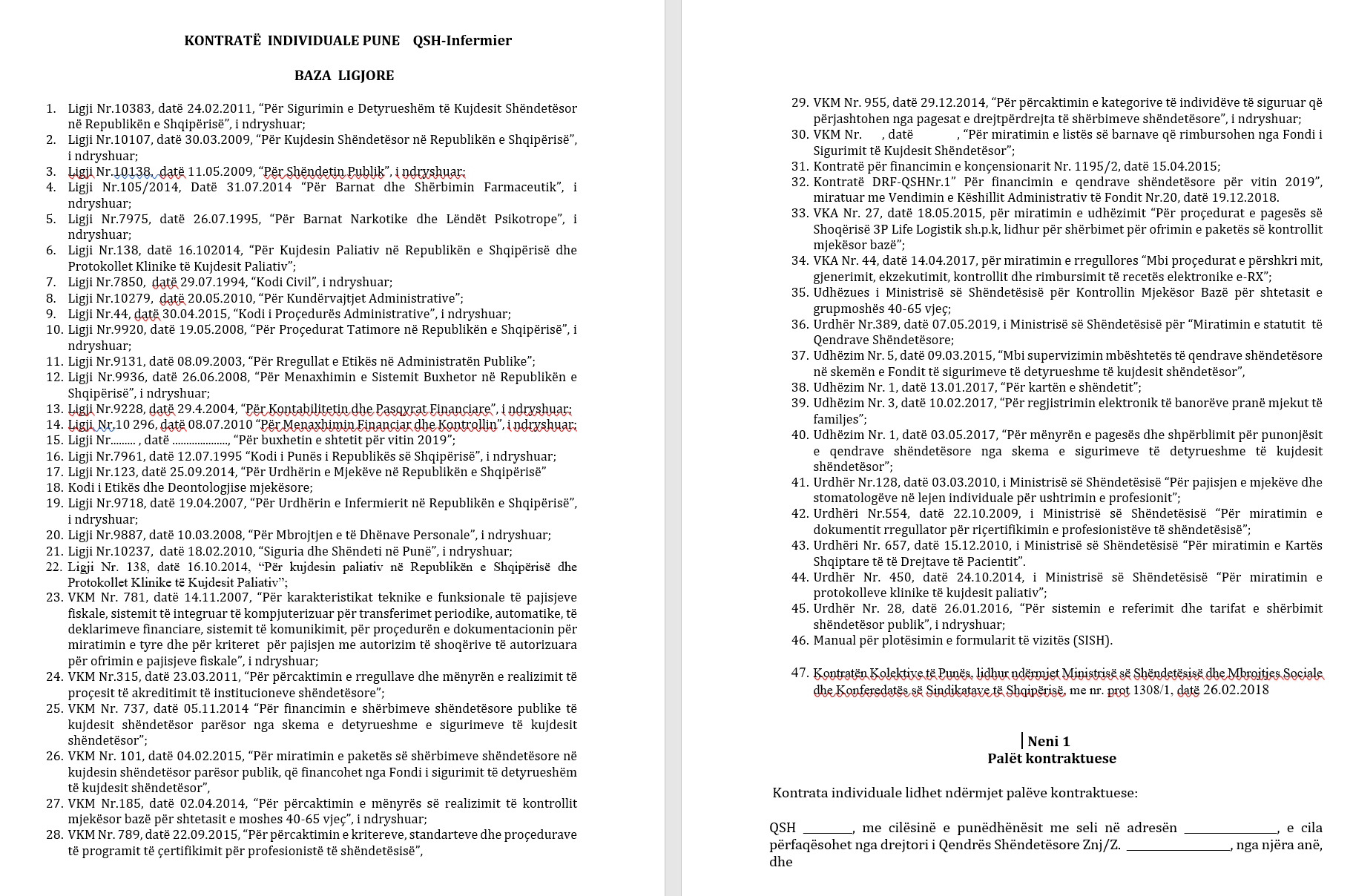


• **The nurse has the appropriate education and makes a judgement that they are competent to perform the activity.**

The majority of nursing staff (over 70% as reported by the Nursing Order representative) is highly educated, thus having sufficient knowledge of nursing care.

However there are activities where FHN nurses feel less competent to conduct (assessed by the various studies and analyses presented earlier in this report).

Design and implementation of education and training programs based on these needs will enable competent professionals to perform the activity. Thus, the opening of the new Professional Master in FN, implementation of the training in workplace, trainings provided by the HC itself in accordance with the real needs of their FHN staff, etc. provide guarantees for better service in response to the needs of the local community.

**• The activity is consistent with accepted standards**

Standards of nursing practice in Albania provide guidelines for nursing performance. They are the rules or definition of what it means to provide competent care. The registered professional nurse is required by the Albanian legislation to carry out care in accordance with what other reasonably prudent nurses would do in the same or similar circumstances. Thus, provision of high quality care consistent with established standards is critical.

However, in the context of FHN nurse in Albania clear-cut definitions related to standards of nursing practice should explicitly embrace the following three components^[[65]](#footnote-65)^:

- Professional standards of FHN care should explicitly define diagnostic, intervention, and evaluation competencies.
- FHN performance standards should identify role functions in direct care, consultation, and quality assurance.
- FHN specific standards of care for specific populations should be developed.

• **The activity to be undertaken by the nurse is appropriate for the context**

Nurses today work in a dynamic health care environment^[[66]](#footnote-66)^. Their roles and functions are constantly evolving and changing to meet patient needs as well as incorporating service needs such as workforce shortages, skill mix issues and budget constraints. If nurses are not supported in making scope of practice decisions, this has the potential to impact negatively on both the quality of patient care and the profession of nursing. The fact that there is a greater need for health care makes the strengthening of the role of the nurse indispensable. This new profile should also be expanded in the context of new services in response to the increased needs of the population, the advancement of technology and the introduction of new services offered electronically in PHC (prescriptions, referrals to specialist doctors and new services provided by the MoHSP) as well as developments in treatment modalities^[[67]](#footnote-67)^.

1. **Factors, opportunities and barriers that can impact the organization and provision of a more comprehensive set of PHC services**
2. **Understanding of PHC and common aims^[[68]](#footnote-68)^**

- PHC services are considered basic health services in the Albanian Health System efforts to control disease and to protect the health of all citizens. In fulfillment of this aim, the Basic Package defines the Health Centers as the main unit that provides PHC services, not through a single facility but through a network of centers and posts reaching all communities for the provision of essential health care services. In this context, the Package has been redefined including also the characteristics of the services offered by the HCs together with the duties and abilities of the physician and the nurse. Specifically, the individual work contract between the HC-Nurse defines the duties of each nurse employed in a HC. The Health Center receives the contract from the Regional Directorate of the CHCIF, where the Nursing Work Package is an annex to the contract with the Regional Directorate of the CHCIF.
- In practice, except the medical check-up program nurse, no other types of nurses have clearly defined duties according to the contracts (except few HC such as Patos, which has started this process, supported by HAP).
- Nurses recognize their general obligations, but they know very little about their specific tasks defined in the package of services (*“I started working through the Portal. The lawyer issued me the working documents. Duties include: compliance with the working hours, coordination with the physicians,* *not to cooperate with the pharmacist”* – report a new nurse in HC 1”).
- Physicians also do not know the specific duties of the nurses stating that: *“We really do not know the tasks of nurses, because traditionally we are not attentive to recognize the contract r s”*, but some physicians believe that: *“Head-nurse should inform us about the specific duties of the nurses in order to avoid the overlap of each-other duties ”*. However, the predominant opinion is that: *“FHN should prepare the working place including the patient, should keep the register, should fill in the register of health visits, should take care of the documents, and should help the physician during the health visits”* (*specifically, the nurse should simply hep with dressing and undressing of the patients)*.
- Lack of contracts with clearly defined tasks affect not only the quality of health care provided to the population , but it also imposes a secretarial role to the nurses (*“The role of FHN depend on his/her work contract, but if their competencies do not have a clear legal basis they will continue to serve as secretaries of the physicians”* – representative of the Health Care Operator in Tirana).

1. **Clear understanding of the role from all team members regarding the responsibilities of each member of the team**

- There are good practices regarding the orientation of the new nurse with the job profiles of the FHN before he/she starts work in his/her place. In the first week of work, he/she can spend every day in new services. When the HC director finds that his or her nursing staff lacks experience or inability to perform specific services properly, especially manipulations (such as vaccination or blood transfusions), then the nurse is trained on the job training. The choices are in services that enable them to practise many duties and in healthy populations such as basic medical control services (*“For Nurses coming unprepared from schools to improve their competences, we have a training work plan in the HC that begins with the check-up service to teach venous access (so that it can be applied to infants) to patients who are healthy”* - doctor, Head of Patos HC). *“At our center, the new nurse goes to the Public Health Directorate and is trained near the nurse responsible for the vaccination procedures and undergoes a test there, for the expertise he/she hasacquired”* (doctor, Fier).
- Regarding the regular formalized inter-professional communications they are almost absent, but there is no lack of sharing of difficult case management experiences among small social groups (*“We do not share experiences with each-other”* – representative of Tirana FHN, adding that: *“We do small group discussions when faced with certain situations, such as the case of drug addicts we did not know how to manage”*).
- Another form of communication is sharing information with colleagues of each FHN participating in a particular training (*“We took an initiative that we discuss together with all the materials and experience from our college received during the training, and therefore we all work to implement the new practice. In this way we create our own rules of procedure that are known and accepted by all”* - doctor in Patos).

1. **Recognition and acceptance of competencies and scope of practice of different team members**

- Generally there is a spirit of cooperation and respect among nurses. Even one nurse says: *“Our strengths are our relationships between each-other”*. A friendly environment has also encountered new staff in the workplace (*“When I started working, I didn’t have practical work experienceat HC and all my colleagues and doctors helped me to learn the job”* - FHN Tirana). FHNs are supported and felt appreciated by the doctors they work with and the HC leaders (*“We do not feel under psychological pressure. They support us.They consider our personal needs as well”*). However, there are opposite situations (*“I complained to the director that I had to work for about a week more overloaded and had to go home too late. In addition to the fact that we had to go two times a day at work the e Sunday service for injections is added. The director said to me that If I wanted I could quit my job”* - nurse in rural areas Fier) (*“When my twin children were 1 year old I had to work two shifts in the village while ma family and I were living in the city . It was very tiring for me that period”*).
- Patients generally regard FHN as the physician’s assistant or secretary and refuse to receive care from them because they are not confident that they are capable (*“Our mentality is that we only want the doctor”. “I would accept an advice from a nurse, but I would not apply it without asking the doctor”. “The nurse has no professionalism. We rely more on doctors”* - said some patients). The nurses feel offended because they believe they are able to provide nursing care but are not allowed to express their abilities. *“When we start advising patients about the medication that the doctor has given them, the patients say that they will see the doctor and they don’t trust us”* - FHN Tirana. They think that this problem is not just a patients trust issue , as in similar situations support should become even from the doctor.
- The mentality of the community, doctor and government regarding the figure of the nurse, (according to the Nurse’s Guide Representative), are difficult barriers to overcome. The figure of the nurse is underestimated. *“In order to improve nurses’ image”, according to an NGO representative, “promoting the positive models it's needed”.* FHNs claim various experience in their field of work. There are FHNs that are allowed by the physician to perform their nursing services, but there are also times when the physician does not even accept the concept that the nurse can make manipulations such as blood pressure measurements (*“When I am in the room there is no chance for the nurse to measure the blood pressure, only in case when I am busy”* - doctor HC No. 1).
- There are cases when a specialist doctor bypasses the nursing care service. (*“The gynecologist does not appreciate the work of the mother care center. This center having no knowledge of the pregnant woman’s history makes it difficult to it to subsequently plan vaccines or address other health issues”* - FHN Tirana).
- Often FHNs change their job position according to the needs of the HC and the planning done by the nurse. Not everyone receives the temporary circulation of workplace well. (*“Managing staff absence is a difficult task for me as head nurse. Sometimes nurses have to go to the Check-up program and they don’t like or don’t accept changing their workplace. Often, I choose to go alone to avoid conflict. We only need to stay for a few hours, (mainly in the first few hours,) and then followour tasks”* - HC No. 1, Tirana).
- In the opinion of the representative of the Tirana Health Care Operator, the frequent movement of FHN from one service to another becomes an obstacle to the fulfilment of the tasks they perform in their workplace. The solution would be to create an FHN structure available to the Operator, which would enable the management of urgent HC needs. *“Model similar to substitute teachers”* - (representative of Tirana Health Care Operator).
- More autonomy is proposed for the head nurse. The skills of the head nurse regarding planning and motivation are scarce. The head nurse is often a nurse’s tutor who does not support them.
- Establishing a manager who only performs the managerial work of the health institution he leads would provide better management of HCs. (*“The workload of the doctor does not allow him/her to perform the job of the Director properly”* - Representative of Tirana Health Care Operator). Likewise, the frequent change of leadership undermines the functioning of the institution (*“In our institution that is very young, 4 leaders have been replaced so far”*).
- Establishing a specific Nursing Care Directorate in the MoHSP would properly address nursing care problems. Cooperation with the municipality and social workers is considered necessary.
- Introducing joint care plans and improving the role of PHC nurses as case managers for patients with multiple morbidity and complex health needs is suggested (*“The pursuit of Diabetes, Hypertension or Vaccination Needs Action Plan”* - FHN Fier).
- Delegation of competencies between FHNs in some HCs is common practice and subject to monitoring, even by the HC manager. (*“We replace each-other in every field of service and every 6 months the director checks the documentation for us”* - FHN Fier). However, FHNs are not always cooperative and delegate competencies to their colleagues (*“I do not delegate the duty of vaccination to other colleagues because it seems to me to be too delicate, as they do not recognize the children / patients in my area”* - FHN Fier).
- When selecting FHN from the list of winners from the portal, it is suggested that the rural and remote areas be given priority to the resident nurse, and be paid extra hours in community service. This would enable continuous and timely service delivery to the community / patients’ area, and more motivated staff than ever (Doctor, Fier).
- A part of the quarterly internship that the graduate performs at university hospital centers (to obtain a license to practice the profession from the UISH) is suggested to be conducted at the HC. This would make him/her more professionally skilled but also make the right choice of the field he/she would work in (PHC or hospital). ). However, applying the practice to HC in today's conditions does not provide the assurance that nurses will receive the right skills as the physician dominates the nurse.
- Participation in training activities is formal, not guided by the need for training in areas where nurses can develop/enhance their skills for better work performance. They claim that the criteria for selecting training are the high number of credits and the low fee for participation. On-line training is not used by the FHN at all.
- In health centers, managers do not assess training needs. The provision of continuing education by the health centers themselves is not performed properly. Health centers do not use the 5% of the budget allocated for the education of its staff within the center because they do not have facilities for their organization (HC 1) or the centers’ budgets are low and they use this part of the budget for other needs (Rural area,Fier). In some HCs (such as Patos HC), with the support of HAP, new training approaches in the workplace have begun to develop in-house training centers with peer groups. These trainings are based on the needs of the FHN staff evaluated by them, there are no participation fees or other cost, and there are credits depending on the duration and type of the trainingA facilitator factor is the willingness of the nurses to participating and professionally benefiting from these activities. The fact that there is a greater need for health care services strengthens the role of the nurse (HAP).
- The biggest contributing factor is continuing education. The supportive attitude that NCVE maintains has become a supporting, facilitating, and developing factor for training nurses in the workplace.
- In the FHN education framework, the Nursing Order contributes to the design of curricula at different levels of education as closely as possible to the real needs of family nurses. There is also a recent collaboration with the Faculty of Nursing to develop the new Professional Master Program in Family Nursing. Regarding the Role of the Order in the implementation of different training courses for continuous professional development of nurses, to date there have been no activity focused on family nurse. In the case of organizing activities for nurses they consisted of lecture format activities and with a large number of participants (NCCE representative / FHN, Tirana). There have been times when even in enormous capacities such as the Palace of Congresses, nurses could not secure entry to the hall due to the extremely large number of participants (medical emergency training).
- Another concern is permission from the directors to participate in training activities that require participation throughout the day. Staff is often required to return to the center and continue their work on the same day, although it is the institution’s duty to encourage staff participation in training and to ensure continuity of work with other staff. The lack of staff in fact made this organization difficult for the HC to manage, affecting the provision of care.
- Technological advances in information and communication are creating new opportunities for local and national healthcare delivery. This requires first, nurses to be trained to gain skills in computer and software uses (*“training especially for the oldest nursing staff”- doctor Tirana)* which will enable new future models of interviewing, diagnosing, describing and facilitating treatment and care, as well as evaluating and providing follow-up.
- The fact that the Draft Strategy places emphasis on nursing, opens the legal basis for enhancing its role. Also, basic care / contract packages are tools that support the nurse to develop and deliver more professionally. *Check-up is a resource for nurse development, but training support is also needed* (doctor,Fier / Interview, HAP).
- The autonomy that the directors of the centers have in organizing the center is a facilitating factor for the organization and provision of a more comprehensive set of PHC services. But local, regional and central authorities do not recognize this autonomy of health center directors. These authorities only do the role of controller (HAP representative).
- The role of the nurse in the community approach comes in two different ways from different actors. One approach is to strengthen the role of the family nurse (representative of MoHSP). *“To be realistic and operational, we must rely on the capacities we have at our disposal today as change needs time.. A good start would be with the HPs nurse who serves without a physician”* (HAP representative). The second approach sees Home Care in the large HCs as a separate unit (Patos): *“If patientsbecome more awarethey can get on home care services, the need to have a separate unit will become evident”*, she argues. The different distribution of patients’ load is a determining factor.
- Patient satisfaction is a performance indicator of health care quality. Patients’ satisfaction is subjective and depends on patients’ perceptions in relation to their expectations. So far few centers have ever applied patient satisfaction measurement, but do not have an evaluation report (Patos, physician). One center also expressed the need for training on how to measure employee and patient satisfaction.
- In the context of this consultancy, an interview was conducted with a group of HC no.1 patients who were asked about some elements of service satisfaction in their health centerin general and about FHN in particular. It turned out that the problem was the infrastructure of the health center, while they were very satisfied with the service provided by all staff. This assessment is considered a restrain to the improvement and development of the role of the FHN as it does not serve as a driver in the process of expanding and improving the role of the FHN and the PHC service as a whole.
- Relationships (job satisfaction): The main purpose of measuring job satisfaction is to identify the key factors of internal and external motivation, evaluating their relevance to employees, and to build the relationships that exist between satisfaction, motivation, commitment and outcomes ofthe work. The health centers have not done such a measurement so far. Except for one center which did not produce an evaluation report as their employees were not freely expressed (doctor in Roskovec), making the results unreliable. There are also centers that claim that *“we try to evaluate employee satisfaction at monthly meetings”*, but without using a standardized tool.
- Nurses do not feel motivated in their work not only forinadequate working conditions, but for the low payment they receive as well.The difference is also in the way of giving bonuses to the health centers. The bonuses are distributed differently, or equally for all nurses, or with small differences. FHNs that receive the differentiation claim that they would feel more motivated if the director would give them the reason for making the difference (FHN Fier). A negative factor is the lack of FHN’s workload measurement and its lack of documentation, which precludes an evaluation of the quality of care in the inpatient care of the FHN. Doctors are paid based on the number of the residents for whomthey provide the services, while the nurses are not.

1. **Appropriate infrastructure (Access to patient information / Physical space / systems / processes)**

- Health centers have many infrastructural problems (*“premiseswhich are small, airless, with no sufficient light”* defines a KI interviewee,). Many are old and/or adaption of abuilding. Thus, the premises of a residential building have been adapted as buildings for HC No.1 in Tirana, where thedoctor’s visiting rooms are for the most part very small in size, with no natural light but mostly with insufficient powerartificial lighting. Also, there is lack of natural ventilation or ventilation systems, some rooms do not even have windows, 24-hour water shortages and only a few services such as surgery, gynecology and injectionshave hot water. (A *nurse reports that she is suffering from bronchial asthma and allergic rhinitis due to this poor working conditions*).
- Often the building spaces are insufficient for the capacity of the population to cover and with basic deficiencies such as lack of water and toilets (the balcony of the kindergarten in DukasBreg, Fier with an approximate 3x3 m has been adapted as a HC for 1000 inhabitants of the area) (*“In Shishtavec which is an area of ​​5600 inhabitants the center had neither drinking water nor sanitation”* - NGO representative).
- *“The visiting rooms are very small, without air conditioning. Doctors and nurses work in difficult conditions”*. *“My complaint is about the infrastructure of the HC”. -*are the patients’ perceptions of the HC no. 1 in Tirana).
- There are no dressing rooms, toilets for staff, but people are reluctant to comment on working conditions (Order of Nursing representative). Occasionally there is only one dressing room for all staff which is not enough, or the staff uses the visiting room as such.
- Although many other criteria are manageable to be met, sometimes infrastructural deficiencies does not allow the HC to complete the accreditation process, (“for the accreditation of our center, we do not meet the basic requirements including lack of natural light” – HC no. 1, Tirana).
- There are deficiencies in the environment (lack of injecting rooms) or in working tools/equipment which create obstacles for the nursing staff to do their job aproprely, thus reducing the nurse’s professional image in front of the patient (emergency package or aerosol deviceis lackingin Tirana). (*“We only have the nurse’s kit provided by HAP” -* claims one of the nurses of a rural area in Fier;*“**When I go to make a visit I would need to take the sphygmomanometer with me, butas we have only such a one in our visiting room, if necessary the doctor takes the sphygmomanometer to another visiting room”*. *“I have been doing this job for 4 years and have no motivation to stay. I have no tools or scales for children and ever since this has become a source of conflict”*- nurse HC no. 1 Tirana).
- There is also a shortage of tools and consumables:*“We have no consumable materials”*;*“We have nooptions of sterilization”*.
- There is also a shortage of vehicles to enable volume and time service and the follow up at home (*“We can only do 1 or 2 visits a day because we go thereon foot. We cannot do periodic follow-up of chronic illnesses. We only go there when we are asked for”*).
- The ambulance (as a means of transport) inside does not have the necessary basic materials for emergency medical treatment. There are times when the barrel should be carried by nurses all the way, preventing proper assistance, sometimes even outside the physical reach of health personnel.
- The centers are equipped with computers (with the exception of rural HC where there is no doctor), but they are used only by the doctor. There is also Internet connection, but there are occasional breaks or blockages of the referral system making it difficult to use (most often on Saturday or Monday).
- Issues that require addressing support for guidance on clinical guidelines and protocols that are included in basic packages remain to be identified, but monitoring of them may be desirable (MoHSP representative).
- Both physicians and nurses articulate the need for nursing practice protocols especially in NCDs that are more prevalent in the population *(“Family Nurses need Emergency , HTA and Diabetes Protocols / Guidelines , CVA Post - STROK Nursing Diagnosis”* (Doctor, Fier). (*“There is e urgent need for nursing protocols for primary health care with the specific focus and priority on home care”*) - according to the representative of the Tirana Health Care Operator.
- There is also a shortage of supporting materials used for health promotion, especially for young people and adults (*“For children 0-2 years old the job is done very well as we have a lot of nutritional information materials”*, FHNFier).*“Often the materials are provided by the nursing staff printing the materials from the IPH website or usingmaterials provided by HAP. The health promotion unit in the district of Fierprovides supportingmaterials, but there are insufficient”*.
- Difficulties in following upchronic patients are encountered as the referral system does not allow the personnel to provide timely service to chronic patients who during follow-up need a rapid planning for examinations (*“The system does not allow us to provide the service properly. In the case of chronic illnesses if they have any urgency, we cannot schedule biochemical test because the system does not allow us. For example, if the patients with diabete need a glycemic control or a* glycohemoglobin, *I can’t give the recommendation, the patient has to go to the specialist”*). (*“The referral system does not allow for rapid planning for pregnant women e.g. to do urgent blood tests for anemia. The woman should wait according to the system even a month, while her healthgood. Often she cannot afford the costs of tests in the private sector”* - Nurse in Fier.
- Although institutions have a system for determining the exact time of visit, patients often wait in line for the planned visit (according to patients interviewed in Tirana). One of them suggests the following: *“I would give an opinion that when we come to informationpoint of HC, the staff there should give us a waiting number ticket and then to go to the family doctor or the specialist one”.*
- The personnel feel unable to manage an incident as they do not have regulations and procedures for such situations (*“Our center has no protocol whatsoever to act in case of any human error during manipulation and injections”* – Nurse in Fier).

1. **Proper allocation of time**

- There has so far been no evaluation of the use of working time in PHC. However, nurses claim that the time taken with documentation / statistics is longer than the patient contact time, even though the electronic prescription and on-line referral system has reduced this working time (nurse in Fier). It is also difficult to follow up chronic illnesses at home, due to the lack of timely coordination with the workload (*“There are times when we do not have time to handle the workload. Therefore, a doctor should make a family visit plan*”).Training for the organization of working time was considered necessary by the nurses.
- For the FHN in the villages additional time is the way round trip, characterised by road infrastructure difficulties, distance from the residential center, application of two-time breaks, often unaffordable by staff. There are times that this is a serious challenge (*“I live in the city and I have a young child. I work in a rural area with 198 inhabitants and do all the tasks on my own. I do 8km of commuting and twice a day as I have to perform afternoon injections. I am not reimbursed for transportation. I do this job and have no motivation to do it”*- FHN affirms that he has completed all employment procedures in Germany and is awaiting a contract).
- The number of nurses in the villages is very low. Services are increasing but the number of nurses is not increasing, whereas the workload continues to grow.

1. **External change agents who can formally influence or facilitate decisions in a desirable direction.**

Below there is a list of some external factors most frequently cited by interviewees:

- The merging of the two ministries (Health and Labor Ministry) i.e. MoHSP. This Ministry will serve for a better inter-institutional cooperation between local government actors and its subordinate institutions for the improvement of community health services with the focus on the inclusion of all health centers/health posts. It will also enable the inclusion of social workers/psychologists, etc. in primary care (there is already a small number of social workers in the hospital service) to contribute to multidisciplinary health teams and new links to local government.
- Involvement of various national or international organizations such as WHO, UNFPA, UNICEF, TAIEX etc; NGOs operating in the field of health and welfare; nursing faculties (public or private) for technical and financial support, successful exchange of experience in the field of education (program design and implementation) and nursing care delivery in PHC can influence to go toward the desirable direction.
- Associations that can help to promote the image of the nurse. They may lobby and advocate to the Albanian parliament to strengthen the figure of the nurse. Also to be able to perform employee satisfaction assessments in the workplace for FHN (not done to date).
- Local government, which should play a greater role in the management, maintenance, rehabilitation of HC premises. Improving working conditions is an important motivating element for staff.
- Government structures that need to provide the right policies and mechanisms for functioning. Nurses and young people should be motivated not to leave the country. Migration, free movement of people is a negative factor in the context of a demotivated society.
- State Police structures in dealing with aggressive patients with mental, alcohol, drugs or problems; in the case of road accidents on timely arrival at the scene as the medical emergency cannot intervene to provide assistance without police earlier presence; or for completing the death certificate.
- HAP, which has enabled the team work at the HC by supporting and motivating all the staff. HAP has done the job of facilitator in the work to build the necessary capacities of the HC.As a potential facilitator for introducing and implementation of new primary health care nurse profile. HAP since 2015 has supported in a  motivational and supportive way organizational development of the Primary Health Centers in Diber and Fier region,  including aspects such as organizational structures and processes influencing Family doctor and nurse behavior and motivation, organizational learning, knowledge management and implementation of organizational and functioning norms, rules and values, essentially through strengthening capacities of the team of Family Medicine .

## **CONCLUSIONS**

- Nurses respond to the health needs of people in all settings and throughout the lifespan. Their roles are critical in achieving global mandates such as universal health coverage and the Sustainable Development Goals.
- In many Primary Health Care (PHC) settings, having nurses as full members of the PHC team is essential to meet the complex health and social needs of the populations. PHC service delivery by nurses has been convincingly linked to improved quality of care, efficiency and decreased cost.
  - 1. **POLICY AND LEGAL FRAMEWORK OF THE NURSE PRACTICE IN ALBANIA**
- Government’s primary role in professional nursing regulation is in establishing appropriate legislation. Statutory regulation should be designed so as it promotes nursing’s ability to respond to societal needs and supports nursing’s role in health care services and in meeting national and international health-related objectives^[[69]](#footnote-69)^.
- The legal framework that regulates the nursing practice in the Republic of Albania includes statutes, laws, decrees or ordinances rules and/or regulations. This legislation ensures the formation and functioning of the role of the nurse in health care, starting with nursing education programs at the university ( undegraduated and postgraduate level), obtaining the license to practice the profession, the conditions and manner of applying for employment, the code of ethics and deontology, individual employment contract, the task and what they should be able to do, the criteria, standards and certification procedures, and the like.
- Today, new skills and responsibilities of nurses in primary health care are needed as there is a change in the health needs of the Albanian population. In a rapidly aging population and an ever-increasing number of patients with complex needs, currently, services need to be provided to the individual and the community, not only in health care institutions but also in-home care.
- The development of new nursing profiles seems to be quite feasible. The legal and regulatory framework offers no barriers to a more autonomous role for nurses, and there is some readiness by the stakeholders towards the implementation of new nursing profiles within the existing BPS framework.
- Particular attention should be paid to establishing common goals and reaching mutual understanding with the key stakeholders.
- The BPS seems to provide a flexible framework for developing the preferred nursing role(s), thus allowing for a pragmatic approach to adapting actual profiles of health professionals in PHC without a great need for potentially lengthy adaptations to the legislation and professional regulation. Such role expansion also seems to be in line with the new PHC strategy for Albania.
  - 1. **ANALYSIS OF THE CURRENT PRIMARY HEALTH CARE SECTOR (STRENGTHS, WEAKNESSES, OPPORTUNITIES AND THREATS)**

**SWOT analysis of PHC services in Albania**

| **STRENGTHS** | **WEAKNESSES** | **OPPORTUNITIES** | **THREATS** |
| --- | --- | --- | --- |
| - Good tradition in PHC sector - Availability of health workforce with basic training - Health centers and posts available all over the country - Basic equipment in place | - Outdated PHC facilities - Fluctuation in the continuity of care - Poor communication - Insufficient management training - Poor use of healthcare informatics - Lack of sufficient financial resources | - Strong commitment of the government - Upcoming PHC Strategy, Albania 2020-25 - Increased PHC funding - Newly established Health Care Operator - Shift from hospital care to PHC services - Introduction of modern technology | - Political insecurity - Rapid increase of population’s expectations - High turnover of the workforce - Migration of the workforce - Increased demand for (expensive) medical technology - Pressure for cost reductions |

- - 1. **HEALTH AND SOCIO-ECONOMIC NEEDS OF THE POPULATION THAT ARE NOT MET BY THE PRESENT PHC AND SOCIAL SERVICES**
- The number of visits per capita per year in Albania remains lower than in other European countries. PHC in Albania needs staff distributed according to the community health needs.
- Currently, there is a need to reorient the PHC services in order to address the toll of non-communicable diseases. An important part of these diseases can be managed and controlled at the PHC services in a cost-effective manner without the need for hospitalization or treatment. To be effective, their control must be comprehensive; primary prevention through healthy lifestyle support, early diagnosis and good management through counselling and treatment. Early identification and effective management of hypertension appears to be a primary challenge for PHC in Albania.
- PHC should also be adapted to the specific needs of vulnerable populations (isolated elderly, people with disabilities, Roma community, etc.). They are more exposed to health risks and are less protected from adverse effects of these risks. Currently, PHC services for vulnerable groups are separated from social services. Both services need to be more coordinated and integrated.
- With regard to palliative care, although the coverage with such services is being gradually expanded over time, the needs are not met. Challenges in these areas concern the implementation of the new law on palliative care; establishment of palliative care units in all regions of Albania, establishment of palliative care for children and expanding to other non-cancer patients; expanding of the number of qualified professionals through postgraduate palliative care courses, etc.
- Data show a discrepancy between the use of services and the prevalence of mental health problems in Albania. As a matter of fact, significantly greater barriers exist to receipt of mental health care in comparison with physical health care in the Albanian population. Public health approaches to stigma and discrimination can facilitate access to mental health care in Albania and FHNs can play a pivotal role in this regard.
- Regarding the medical care, older people could not access medical services when needed, with the main hindering factors difficulties to afford medical treatment followed by the long distance to medical facilities and the inability to attend medical services due to the presence of severe illnesses/pains. They also have difficulty purchasing medications described by the doctors. These inabilities were linearly and positively associated with the age of older people and rural living area.
- Traditionally, long-term care has been shared between two separate sectors, health and social sectors. The merging of the two formerly separate ministries (health & social affairs) may provide synergies and better coordination and a more adequate response to elderly care. However, to date, there is no formal (institutionalized) long-term care in Albania. Yet, the services provided are not adequate and there are very few physicians who have received specific training on geriatrics and gerontology.
- There are no home-based social services for older people in Albania, putting enormous pressure on close family members who should provide support for their older relatives. In this context, long-term care expenditure for health services, especially for social services, is provided by close family members; including remittances from emigrants. However, the traditionally strong family bonds are currently fading away, including the provision of remittances. Therefore, the role of public services becomes even more relevant.
- Currently, there is no link between the social insurance systems and social and healthcare provided by the active workforce to their elderly family members who need continuous assistance and daily care. There is no financial assistance provided to the families who provide care and support to their older relatives, except for the poor individuals, who receive an economic aid and electricity bursary.
- In brief, the pivotal role of both central government and local government for supporting long-term care in Albania is inadequate. Several NGOs try to fill this gap including some religious associations.

**CORE FEATURES OF FHN IN THE CONTEXT OF ALBANIA**

1. **Distribution**

- In the PHC system in Albania is a large number of nurses, but their distribution varies widely by region/prefecture, as well as urban/rural areas.
- The pressure of external and internal migration remains a threat to the future. The situation is rendered even more complex considering the internal and external migration of the workforce in particular of the nurses who may seek more attractive jobs abroad. No detailed analysis has been carried out yet for the nurses in order to better understand comprehensively the driving factors for their migration.
- Internal migration of nursing staff from rural areas to urban areas, has resulted in remote areas still having nursing assistants that play the role of nurse but with lower competence.It is recommended to improve rural retention by using a combination of fiscally sustainable financial incentives, sufficient enough to outweigh the opportunity costs associated with working in rural areas, as perceived by health workers.

1. **Education of nurses**

- The MoHSP needs to clearly define nursing competencies, review and update the job descriptions in terms of services provided by nurses at the PHC level and include also the new responsibilities in the Basic Package of PHC, in order to establish a foundation for new/updated nursing education curriculum at all levels.
- The Faculty of Medical Technical Science is identified as a very important stakeholder, which is expected to have a crucial role in developing undergraduate and postgraduate curricula and providing positive training experiences for FNs.
- It is necessary to strength the nursing education by:
  - harmonization of the teaching programmes among all nursing faculties in basic education (graduate and post-graduate), aligning the nursing curricula to the international/European standards with the focus on practical competencies, abilities and skill-mix approach (TAIEX project), introduction of more PHC subjects in the curriculum to strengthen the capacity of nurses to work autonomously and take larger responsibilities.
  - harmonization of the content of CME activities to correspond to the training needs.
- There is no disaggregated information about the job profile or sector of employment, of the trained nurses, which does not allow for an analysis of the training content or training approach meeting the needs of FHNs for continuous professional development.
- There is a gradual increase of academically qualified nurses in Albania, albeit not matching the current and especially upcoming demands of the population. Also, some new teaching programs are currently under development, the most remarkable one being the upcoming Master program in FHN.
- For a successful education and training of programs development and implementation, it is essential to encourage and support the collaboration and partnerships between FMTS and MoES; MoHSP, CHIF, Professional Associations, Order of Doctors and Order of Nurses and CME Center and different international organizations.
- There are no specific criteria, standards and certification procedures for FN.
- There is a need to continuously evaluate and improve the educational environment of the Nursing.

1. **Remuneration**

- In Albania, salaries of nurses in general are low. Of note, neither the capitation criterion, nor the geographical distributions are applied to PHC nurses. There are no incentives for the nurses operating in the PHC system. There are also incentive policies for retention of GPs at remote areas, but they are not applied to PHC nurses.
- There is a differentiation in the method of payment between family doctors and FHN, as well as among nurses themselves. The difference is in terms of payment, remuneration, bonus and reimbursement of transportation expenses. The remuneration is not based on performance appraisal. Among nurses, only those working at the check-up program can receive performance-based bonus. Hence, it is recommended to^[[70]](#footnote-70)^:
  - Revise contracts and payment schemes for PHC centres, changing input based payment to risk adjusted capitation combined with outcome based incentives.
  - Revise historical salary-based remuneration of PHC doctors and nurses to more flexible options.

1. **Participation in decision-making**

- FHN nurses in Albania are not included in the decision-making process. This is partly due to the heritage of the system which is based on a strong physician-oriented tradition, with little voice for the nursing staff. An additional hindering factor concerns the lack of proper education, qualification and training of the nurses operating at different levels of the health care system in Albania.

Hence, it is recommended:

- The Establishment of a specific Unit on Nursing Care as part of the organizational structure of the MoHSP; nurses to be part of any Boards/Councils near the MoHSP.
- The re-establishment of the Department of Nursing at the FTMS near MUT.
- The wide use of media in order to influence current policies and programs in the Albanian health care sector.
- Nursing Day (May 12) to be used as an opportunity to communicate with the public about the profession of nursing as a profession that requires great dedication.
  - 1. **FACTORS, OPPORTUNITIES AND BARRIERS THAT CAN IMPACT THE DEVELOPMENT OF NEW JOB PROFILES AND PROVISION OF A MORE COMPREHENSIVE SET OF PHC SERVICES *(ad-hoc assessments)***

1. **HR related aspects and specific requirements to exercise new roles; Specific areas where nurses feel confident and less confident in their professional role in the community**

- In primary health care there are needed new nurse skills and responsibilities as there is a change in the health needs of the Albanian population. To meet these health needs of the population today, there must be provided services to the individual and community, not only in health institutions but also at home.
- Recommendations for the new roles should be differentiated according to the local context, separately for Tirana and the big cities, separately for urban areas and for rural areas.
- Due to the increased complexity of the patient’s needs today, the nurses in PHC should work closely to the physician and maintain a cross-professional collaboration with a much broader spectrum of professionals. The need for cross-professional collaboration in the field of PHC has become even more urgent. Both the new responsibilities in patient care and the tasks shifted from physicians to nurses necessitate a redesign in PHC teams and, consequently, a restructuring of cross-professional collaboration.
- Advanced nursing education should be considered as a prerequisite in order to ensure the shaping of the new nurses role in PHC. Nurses need to be trained on new roles to provide new services and proactively engage at the community in concordance with the Definition of the Family Health Nurse (WHO 2000) as well.
- The most structured service that completely fulfills nursing’ duties is mother and child care and vaccination, following by check-up nurses.
- Specific areas where nurses fell confident in their role in the community are different in urban areas (in *fulfilling secretarial, administrative/completion of documentation and chronic illness management, in the use and evaluation of growth curves in children; in the management of emergencies of chronic patients and acute emergencies*) related to rural areas (*in nursing care since working alone the nurses take on more responsibility, have more autonomy with regard to interventions, in provision of more home service, patient health education and in building better relationships with them*)*.*
- The nurses feel less confident in performing manipulations such as paediatric intravenous, catheter placement, etc; management of patients who have consumed narcotic drugs; in the case of traumas/accidents and in properly treating injuries and wounds.
- Home service has not been practiced or practiced very little by nurses because the family member does not trust the nurse and usually requires a physician for the service.

1. **Decision-making processes that can impact the structure of the model or the scope of practice of the family nurse**

- The activity is consistent with the nursing legislation, board policy and guidelines.

The legal and regulatory framework offers no barriers to a more autonomous role for nurses, and there is some readiness by the stakeholders towards the implementation of new nursing profiles within the existing BPS framework. Such role expansion also seems to be in line with the new PHC strategy for Albania.

- The primary motivation for undertaking this activity is to meet patient needs and improve health outcomes.

In the Albanian context this activity should respond to the country’s national health agenda, the burden of disease, ageing population, most vulnerable population groups and the entire Albanian society.

- The activity is appropriately authorised by a valid order/protocol and in accordance with established policies and procedures.

The fact that the Draft Strategy places emphasis on nursing opens the legal basis for enhancing its role. Also basic care/contract packages are tools that support the nurse to develop and deliver more professionally.

- The nurse has the appropriate education and makes a judgement that they are competent to perform the activity.

The majority of nursing staff is highly educated, thus having sufficient knowledge of nursing care. Design and implementation of education and training programs based on the evaluated nurses needs will enable competent professionals to perform the activity. Thus the opening of the new Professional Master in FN, implementation of on-the-work place training, trainings provided by the HC itself in accordance with the real needs of their FHN staff, etc. provide guarantees for better service in response to the needs of the local community.

- The activity is consistent with accepted standards

Standards of nursing practice in Albania provide guidelines for nursing performance. The registered professional nurse is required by the Albanian legislation to carry out care in accordance with what other reasonably prudent nurses would do in the same or similar circumstances. Thus, provision of high quality care consistent with established standards is critical.

- The activity to be undertaken by the nurse is appropriate for the context

Nurses today work in a dynamic health care environment^[[71]](#footnote-71)^. This new profile should be expanded in the context of new services in response to the increased needs of the population, the advancement of technology and the introduction of new services offered electronically in PHC as well as developments in treatment modalities^[[72]](#footnote-72)^.

1. **Factors, opportunities and barriers that can impact the organization and provision of a more comprehensive set of PHC services**

- PHC services are considered basic health services in the Albanian Health System efforts to control disease and to protect the health of all citizens. The Basic Package defines the areas of service, the institutions, the personnel involved along with everyone’s competencies and duties. The individual contract between the employee and the HC regulates the working relationships.
- In practice, except the medical check-up program nurse, no other types of nurses have clearly defined duties according to the contracts. Lack of contracts with clearly defined tasks jeopardizes not only the quality of care, but it also imposes a secretarial role to the nurses.
- Doctors and nurses do not understand the professional identity and specific role of each other in the care process and not always their relationship is based on mutual respect and trust. These factors play an important role in their effective functioning as a team.
- In Albania, traditional hierarchies continue to exist between professions which become obstacles to effective cooperation. Doctors find it difficult to shift tasks to their nurses even though they complain of overloading. Even when they do, they actually shift tasks that are often their responsibility.This misuse of the practice nurses to reduce the doctors’ workload has been identified as major obstacle of teamwork.
- Nurses and doctors in Albania collaboration relatively well. But, it seems that the collaboration is driven by the interest of the nurses and the dependency relationship they have with the physician and the limited practice of their work. However, there are other factors that influence the perception of collaboration such as the personality or expectations of everyone from a particular relationship, etc.
- Nurses lack protocols that clearly define treatment measures and methods in their work, and when they should consult a physician or refer a patient to a physician (in the case of home care).
- There are good practices regarding the orientation of the new nurse with the job profiles of the FHN before he/she starts work in his/her place.
- Regular formalized inter-professional communications are almost absent, but there is no lack of sharing of difficult case management experiences among small social groups.
- The mentality of the community, doctor and government regarding the figure of the nurse, are difficult barriers to overcome. A lot of improvement should be made to change the position of family nurses in the society.
- The role of the nurse in the community approach comes in two different ways from different actors. One approach is to strengthen the role of the family nurse. The second approach sees Home Service in the large HCs as a separate unit.
- Undergraduate education is not sufficient for proper knowledge and clinical practice.
- The biggest contributing opportunity is continuing education. Organisation of training activities should be guided by the need for training in areas where nurses can develop/enhance their skills for better work performance. The Order of Nurses could have an important role in prioritizing educational themes for FNs throughout continuing professional development. The positive attitude that NCVE maintains has become a supporting, facilitating, and developing factor for training nurses especially in the workplace.
- There are no quality management systems for nursing services.
- In health centers there is a poor physical infrastructure (outside and inside the HC). The HC/HP also lacks equipment and consumables, and support materials necessary for health promotion. This means that the nurse cannot meet the individual and population health needs. In urban settings, HC are better equipped than in rural areas. There is a need to develop and implement a plan on improving working conditions to ensure positive environments and proper working conditions.
- Patient satisfaction is a performance indicator of health care quality which is generally not assessed in health centers. Based on two surveys on patient satisfaction (one survey carried out in two regions of Albania, and the other from a focus group interviews in the context of this consultancy), it turned out that the almost all patients were very satisfied with the service provided by all staff. This assessment is considered a restrain to the improvement and development of the role of the FHN as it does not serve as a driver in the process of expanding and improving the role of the FHN and the PHC service as a whole.
- Nurses do not feel motivated in their work for two major reasons: poor working conditions and the low payment they receive. The health centers have not conducted any measurement of job satisfaction so far.
- Establishing a specific Nursing Care Directorate in the MoHSP would properly address nursing care problems. Cooperation with the municipality and social workers is considered necessary. Establishing a manager who only performs the managerial work of the health institution he leads would provide better management of HCs. More autonomy is also proposed for the head nurse. The skills of the head nurse regarding planning and motivation are scarce.
- The autonomy that the directors of the centers have in organizing the center is a facilitating factor for the organization and provision of a more comprehensive set of PHC services.

**Proper allocation of time**

- To date, there has been no evaluation of the use of working time in PHC. However, nurses claim that the time taken with documentation/statistics is longer than the patient contact time, even though the electronic prescription and on-line referral system has reduced this working time. It is also difficult to follow up chronic illnesses at home, due to the lack of timely coordination with the workload. Training for the organization of working time was considered necessary by the nurses. It is recommended a time allocation measurement to to evaluate the working hours and productivity of PHC providers in their workplace.

1. **External change agents who can formally influence or facilitate decisions in a desirable direction**

- Among the external factors most frequently cited by interviewees were: The merging of the two ministries (Health and Labor Ministry) i.e. MoHSP; Involvement of various national or international organizations such as WHO, UNFPA, UNICEF, TAIEX etc; NGOs operating in the field of health and welfare; Nursing faculties; Associations that can help promote the image of the nurse; Local government; State Police structures and HAP.

**Concluding remarks**

- The development and implementation of new job profiles and roles for family nurses in Albania is considered very important,as like many other countries today, Albania has to face with new challenges in primarycare created by a changing spectrum of illness anddisorders and the growing necessity to find needsbasedsolutions for complex, long-term healthproblems^[[73]](#footnote-73)^.
- Family nursing is found to be partially implemented, mainly because of the weak collective action of stakeholders.Particular attention should be paid to establishing common goals and reaching mutual understanding with key stakeholders in order to have a better family medicine–orientedmodel of primary health care.
- Motivated and task-oriented staff is essential to providing the highest quality of primary health care services. PHC in Albania needs staff distributed according to the community health needs, capable of adapting to the increasingly complex and growing demand for health care services driven by rapid demographic, epidemiological and social change.
- No reform could achieve its goals without addressing the gap and needs in human resources, in particular of doctors and nurses working in the PHC sector.
- It is imported to have an effective teamwork between family doctors and nurses. Therefore their collaboration needs to be based on an understanding of each other’s professional identity and specific role in the care process as well as on mutual respect and trust.
- The participatory working model of HAP with Family Medicine teams (family doctor and nurse) and the Health Center managerial teams (Director, Head nurse, accountant) in PHCs in Diber and Fier is a successful example and good practice of the orientation of the PHC system toward a real family medicine approach. Therefore, this successful model should be scaled up at a national level in Albania

1. International Council of Nurses. Scope of Nursing and Decision making Toolkit.ICN, 2010. [↑](#footnote-ref-1)
2. <http://www.parlament.al/Files/ProjektLigje/20190513145923ligj%20nr.%2027,%20dt.%208.5.2019.pdf>. [↑](#footnote-ref-2)
3. <http://urdhriinfermierit.org/wp-content/uploads/2019/03/Ligji-per-Profesionet-e-Rregulluara-.pdf>. [↑](#footnote-ref-3)
4. <http://urdhriinfermierit.org/wp-content/uploads/2018/07/Ligji-Urdhrin-Infermierit190115.pdf>. [↑](#footnote-ref-4)
5. <http://urdhriinfermierit.org/wp-content/uploads/2018/07/Statuti-i-Urdhrit-te-Infermierit-19072018.pdf>. [↑](#footnote-ref-5)
6. <https://peqini.gov.al/wp-content/uploads/2019/03/Kodi-i-Punes-2018-2.pdf>. [↑](#footnote-ref-6)
7. <http://urdhriinfermierit.org/wp-content/uploads/2018/07/Kodi_Etik-Deontologjik_UISH-19-06-2018.pdf>. [↑](#footnote-ref-7)
8. Nurse Profiles and Master in Family Nursing Mission Mieke Deschodt & Greet Van Malderen. Report September 2019. [↑](#footnote-ref-8)
9. <http://qkcsaish.gov.al/rreth-akreditimit/institucione-te-akredituara>. [↑](#footnote-ref-9)
10. LargimiimjekëvengaShqipëria.Friedrich-Ebert-Stiftung. TFL <https://togetherforlife.org.al/wp-content/uploads/2018/12/Largimi-i-mjekeve-nga-Shqiperia.pdf>. [↑](#footnote-ref-10)
11. Set dokumentash model përakreditimin e qendraveshëndetësore HAP-MSHMS. [http://www.hap.org.al/wp-content/uploads/2019/06/set-i-dokumenteve-model-per-akreditimin.pdf](http://www.hap.org.al/wp-content/uploads/2019/06/Set-i-dokumenteve-model-per-akreditimin.pdf). [↑](#footnote-ref-11)
12. Primary health care in Albania: structures and model of care: Summary of findings and recommendations of comprehensive PHC assessment [↑](#footnote-ref-12)
13. Primary health care in Albania: structures and model of care: Summary of findings and recommendations of comprehensive PHC assessment [↑](#footnote-ref-13)
14. World Health Organization. The Family Health Nurse: context, conceptual framework and curriculum. WHO, 2000.<http://www.euro.who.int/__data/assets/pdf_file/0004/53860/E92341.pdf?ua=1>. [↑](#footnote-ref-14)
15. Strengthening people-centred health systems in the WHO European Region: framework for action on integrated health services delivery. WHO Regional office for Euope. 2016. <http://www.euro.who.int/en/about-us/governance/regional-committee-for-europe/past-sessions/66th-session/documentation/working-documents/eurrc6615-strengthening-people-centred-health-systems-in-the-who-european-region-framework-for-action-on-integrated-health-services-delivery>. [↑](#footnote-ref-15)
16. Primary health care in Albania: rapid assessment. Mission report, WHO European Centre for Primary Health Care. WHO Regional Office for Europe. 2018. [↑](#footnote-ref-16)
17. WHO. Primary health care in Albania: comprehensive PHC assessment report. WHO, 2018. [↑](#footnote-ref-17)
18. WHO. Primary health care in Albania: comprehensive PHC assessment report. WHO, 2018. [↑](#footnote-ref-18)
19. WHO. Primary health care in Albania: comprehensive PHC assessment report. WHO, 2018. [↑](#footnote-ref-19)
20. LIGJ Nr. 27/2019 PËR DISA NDRYSHIME DHE SHTESA NË LIGJIN NR. 10 107, DATË 30.3.2009, “PËR KUJDESIN SHËNDETËSOR NË REPUBLIKËN E SHQIPËRISË”, TË NDRYSHUAR. [↑](#footnote-ref-20)
21. WHO. Primary health care in Albania: comprehensive PHC assessment report. WHO, 2018. [↑](#footnote-ref-21)
22. WHO. Primary health care in Albania: comprehensive PHC assessment report. WHO, 2018. [↑](#footnote-ref-22)
23. WHO. Primary health care in Albania: comprehensive PHC assessment report. WHO, 2018. [↑](#footnote-ref-23)
24. WHO. Primary health care in Albania: comprehensive PHC assessment report. WHO, 2018. [↑](#footnote-ref-24)
25. WHO. Primary health care in Albania: comprehensive PHC assessment report. WHO, 2018. [↑](#footnote-ref-25)
26. WHO. Primary health care in Albania: comprehensive PHC assessment report. WHO, 2018. [↑](#footnote-ref-26)
27. WHO. Primary health care in Albania: comprehensive PHC assessment report. WHO, 2018. [↑](#footnote-ref-27)
28. WHO. Primary health care in Albania: comprehensive PHC assessment report. WHO, 2018. [↑](#footnote-ref-28)
29. Laschinger HS, Hall LM, Pedersen C, Almost J. A psychometric analysis of the patient satisfaction with nursing care quality questionnaire: an actionable approach to measuring patient satisfaction. J Nurs Care Qual. 2005;20(3):220-30. [↑](#footnote-ref-29)
30. Health for All Project Albania. Report on the Assessment of Quality of Care in Primary Health Care Facilities in the two Pilot Regions. Version 2, April 2019. [↑](#footnote-ref-30)
31. International Council of Nurses.Scope of Nursing and Decision making Toolkit.ICN, 2010. [↑](#footnote-ref-31)
32. Primary health care in Albania: structures and model of care: Summary of findings and recommendations of comprehensive PHC assessment [↑](#footnote-ref-32)
33. # Palliative care – Albania. Journal of Pain and Symptom Management 55(2S) · August 2017.

    [↑](#footnote-ref-33)
34. Nursing Order. [↑](#footnote-ref-34)
35. https://togetherforlife.org.al/wp-content/uploads/2018/12/Largimi-i-mjekeve-nga-Shqiperia.pdf. [↑](#footnote-ref-35)
36. Muraraneza & Mtshali, 2018. Conceptualization of competency based curricula in pre-service nursing and midwifery education: A grounded theory approach. [↑](#footnote-ref-36)
37. Muraraneza, Mtshali, & Mukasomi, 2017Issues and challenges of curriculum reform to competency‐based curricula in Africa: A meta‐synthesis. [↑](#footnote-ref-37)
38. World Health Organization (WHO), 2014. Four-year, integrated nursing and midwifery competency-based, prototype curriculum. Brazzaville: WHO Regional Office for Africa. [↑](#footnote-ref-38)
39. The context in which the curriculum will be offered and graduates will practice nursing,for a professional master’s degree programme in family nursing, in Albania. [↑](#footnote-ref-39)
40. The context in which the curriculum will be offered and graduates will practice nursing,for a professional master’s degree programme in family nursing, in Albania. [↑](#footnote-ref-40)
41. Nurse Profiles and Master in Family Nursing. Mission I MiekeDeschodt& Greet Van Malderen September 2019. [↑](#footnote-ref-41)
42. Report: The context in which the curriculum will be offered and graduates will practice nursing, for a professional master’s degree programme in family nursing, in Albania. [↑](#footnote-ref-42)
43. WHO (2018). Draft: Primary health care in Albania: structures and model of care. Summary of findings and recommendations of comprehensive PHC assessment. [↑](#footnote-ref-43)
44. EdukiminëVazhdimiProfesionistëvetëShëndetësisë 2015-2018.<http://www.qkev.gov.al/images/RAPORTI_I_EDUKIMIT_NE_VAZHDIM_2015-2018.pdf>. [↑](#footnote-ref-44)
45. Primary health care in Albania:structures and model of care:Summary of findings and recommendations of comprehensive PHC assessment. [↑](#footnote-ref-45)
46. Primary health care in Albania:structures and model of care:Summary of findings and recommendations of comprehensive PHC assessment. [↑](#footnote-ref-46)
47. Mireille Kingma. [Economic incentive in community nursing: attraction, rejection or indifference?](https://www.ncbi.nlm.nih.gov/pmc/articles/PMC166116/) Hum Resour Health. 2003; 1: 2.  doi: 10.1186/1478-4491-1-2. [↑](#footnote-ref-47)
48. Primary health care in Albania: structures and model of care: Summary of findings and recommendations of comprehensive PHC assessment. [↑](#footnote-ref-48)
49. Nurse Profiles and Master in Family Nursing Mission Mieke Deschodt & Greet Van Malderen. Report September 2019. [↑](#footnote-ref-49)
50. De Maeseneer, J., Strengthening the model of primary health care in Estonia, WHO, 2016. [↑](#footnote-ref-50)
51. Strategy on the development of primary health care services in Albania2020-2025 (Draft IV). [↑](#footnote-ref-51)
52. # Palliative care – Albania.Journal of Pain and Symptom Management 55(2S) · August 2017.

    [↑](#footnote-ref-52)
53. MiekeDeschodt& Greet Van Malderen.Mission I report. [↑](#footnote-ref-53)
54. MiekeDeschodt& Greet Van Malderen. Nurses activities, PPT. [↑](#footnote-ref-54)
55. Strategy on the development of primary health care services in Albania. [↑](#footnote-ref-55)
56. National health report. Health Status of the Albanian Population © Institute of Public Health, Tirana, Albania, 2014. <http://www.ishp.gov.al/wp-content/uploads/2015/04/Health-report-English-version.pdf>. [↑](#footnote-ref-56)
57. National Health Report. Institute of Public Health; 2014. [↑](#footnote-ref-57)
58. Parkar SR. Elderly mental health: needs. Mens Sana Monogr. 2015;13(1):91-9. [↑](#footnote-ref-58)
59. World Health Organization.Aging and life-course. Health promotion for older people: not business as usual. <https://www.who.int/ageing/features/health-promotion/en/>. [↑](#footnote-ref-59)
60. World Health Organization.Aging and life-course. Health promotion for older people: not business as usual. <https://www.who.int/ageing/features/health-promotion/en/>. [↑](#footnote-ref-60)
61. Activities of nurses and midwives in HCs and HPs. September 11^th^, 2019. [↑](#footnote-ref-61)
62. Palliative care – Albania. [Journal of Pain and Symptom Management](https://www.researchgate.net/journal/0885-3924_Journal_of_Pain_and_Symptom_Management) 55(2S) · August 2017. [↑](#footnote-ref-62)
63. Palliative care – Albania. [Journal of Pain and Symptom Management](https://www.researchgate.net/journal/0885-3924_Journal_of_Pain_and_Symptom_Management) 55(2S) · August 2017. [↑](#footnote-ref-63)
64. Scope of Nursing Practice and Decision-Making Framework TOOLKIT.pdf; 2010. <https://www.icn.ch/sites/default/files/inline-files/2010_ICN%20Scope%20of%20Nursing%20and%20Decision%20making%20Toolkit_eng.pdf>. [↑](#footnote-ref-64)
65. Amrican Nurses Association. <https://www.nursingworld.org/>, [↑](#footnote-ref-65)
66. Scope of Nursing Practice and Decision-Making Framework TOOLKIT.pdf; 2010.<https://www.icn.ch/sites/default/files/inline-files/2010_ICN%20Scope%20of%20Nursing%20and%20Decision%20making%20Toolkit_eng.pdf>. [↑](#footnote-ref-66)
67. Scope of Nursing Practice and Decision-Making Framework TOOLKIT.pdf; 2010.<https://www.icn.ch/sites/default/files/inline-files/2010_ICN%20Scope%20of%20Nursing%20and%20Decision%20making%20Toolkit_eng.pdf>. [↑](#footnote-ref-67)
68. <http://www.fsdksh.com.al/images/stories/faqe_kryesore/sherbimet/qsh/Paketa%20e%20Sherbimeve.p>. [↑](#footnote-ref-68)
69. International Council of Nurses. Scope of Nursing and Decision making Toolkit.ICN, 2010. [↑](#footnote-ref-69)
70. Primary health care in Albania: structures and model of care: Summary of findings and recommendations of comprehensive PHC assessment. [↑](#footnote-ref-70)
71. Scope of Nursing Practice and Decision-Making Framework TOOLKIT.pdf; 2010.<https://www.icn.ch/sites/default/files/inline-files/2010_ICN%20Scope%20of%20Nursing%20and%20Decision%20making%20Toolkit_eng.pdf>. [↑](#footnote-ref-71)
72. Scope of Nursing Practice and Decision-Making Framework TOOLKIT.pdf; 2010.<https://www.icn.ch/sites/default/files/inline-files/2010_ICN%20Scope%20of%20Nursing%20and%20Decision%20making%20Toolkit_eng.pdf>. [↑](#footnote-ref-72)
73. Kerstin Hämel, Carina Vössing. The collaboration of general practitioners and nurses in primary care: a comparative analysis of concepts and practices in Slovenia and Spain. [↑](#footnote-ref-73)
