# Supplementary material for: Work time allocation at primary health care level in two regions of Albania
Source: PLoS One. 2022 Oct 26;17(10):e0276184. doi: 10.1371/journal.pone.0276184 (PMC9605026; doi:10.1371/journal.pone.0276184)
Supplement: S2 Text — (DOCX) [file pone.0276184.s006.docx]

| Department Name  Unit Name (optional)  Group Name (optional)  27 September 2019 |
| --- |
| Nurse Profiles and Master in Family Nursing  Mission I |
| Report |
|  |
| Mieke Deschodt & Greet Van Malderen  [Mieke.Deschodt@unibas.ch](mailto:Mieke.Deschodt@unibas.ch)  [Greet.vanmalderen@unibas.ch](mailto:Greet.vanmalderen@unibas.ch) |
|  |

Contacts

|  |  |
| --- | --- |
| **HAP Center**  Str. Themistokli Gërmenji  Helios Building  Ap. 2/1  Tirana, Albania  [info@hap.org.al](mailto:info@hap.org.al)  [www.hap.org.al](http://www.hap.org.al) | **Swiss Tropical and Public Health Institute**  Kreuzstrasse 2  P.O. Box  4123 Allschwill  Switzerland  [www.swisstph.ch](http://www.swisstph.ch) |

Abbreviations

Disclaimer

The views and ideas expressed herein are those of the author(s) and do not necessarily imply or reflect the opinion of the Swiss Agency for Development and Cooperation, Swiss Tropical and Public Health Institute or Health for All Project (HAP).

| BPS | Basic Package of Services |
| --- | --- |
| FD | Family Doctor |
| FM | Family Medicine |
| FMT | Family Medicine Team |
| FN | Family Nurse |
| FTMS | Faculty of Technical Medical Science |
| HAP | Health for All Project |
| HC | Health Center |
| HP | Health Post |
| HRH | Human Resources for Health |
| INS | Institute of Nursing Science, University of Basel |
| MFN | Master in Family Nursing |
| MoHSP | Ministry of Health and Social Protection |
| NCCE | National Centre for Continuous Education |
| NCD | Non communicable disease |
| NCQSA | National Centre for Quality, Safety and Accreditation of Health Centres |
| PHC | Primary Health Care |
| ToR | Terms of Reference |
| WHO | World Health Organisation |

Table of Contents

[Executive Summary 1](#_Toc22218262)

[1 Background 2](#_Toc22218263)

[2 Mission objectives and approach 2](#_Toc22218264)

[3 Roles and functions of nurses: update 3](#_Toc22218265)

[3.1 Nursing role 3](#_Toc22218266)

[3.2 Findings previous missions 4](#_Toc22218267)

[3.2.1 Roles in HCs and HPs 4](#_Toc22218268)

[3.2.2 Basic Package of Services (BPS) 5](#_Toc22218269)

[3.3 Findings current mission 6](#_Toc22218270)

[3.3.1 Methodology 6](#_Toc22218271)

[3.3.2 Findings 6](#_Toc22218272)

[3.4 Conclusion current nursing roles in Albania 8](#_Toc22218273)

[4 Nurse profiles for PHC in Albania 8](#_Toc22218274)

[5 Master in Family Nursing 10](#_Toc22218275)

[6 Recommendations 12](#_Toc22218276)

[6.1 Development of new nursing roles within the Albanian PHC model 12](#_Toc22218277)

[6.1.1 Potential roadmap for the development of new nursing roles 12](#_Toc22218278)

[6.1.2 Working group members 12](#_Toc22218279)

[6.1.3 Working group operations 13](#_Toc22218280)

[6.2 Development of Master in Family Nursing Curriculum 14](#_Toc22218281)

[6.2.1 Potential roadmap for the development of FN Curriculum 14](#_Toc22218282)

[6.2.2 Working Group operations 14](#_Toc22218283)

[Appendix A: Terms of Reference 15](#_Toc22218284)

[Appendix B: Mission Programme 15](#_Toc22218285)

[Appendix C: PPT Nursing profiles 15](#_Toc22218286)

[Appendix D: PPT FN Curriculum 15](#_Toc22218287)

[Appendix E: Analysis Survey Nursing Roles 15](#_Toc22218288)

[Appendix F: FTMS – draft curriculum 15](#_Toc22218289)

[Appendix G: FTMS 15](#_Toc22218290)

Executive Summary

The nursing profession in Albania needs to be strengthened in order to address the health challenges in the country, especially the increasing burden of disease related to non-communicable diseases and the health care needs of the elderly and vulnerable population. To tailor Primary Health Care (PHC) services to the prevailing burden of disease, roles and responsibilities of PHC personnel, particularly nurses, need to be redefined and nurses need to acquire and be able to use the necessary competencies and skills to work confidently and to a high standard in an extended role. Such a role is perfectly possible within the current legal and regulatory framework in Albania, with the Basic Service Package (BPS) providing a key tool to guide the discussion on the role, skills and competencies of nurses in various settings in Primary Health Care. Such an extend role also fits within the planned PHC strategy.

The main objectives of this consultancy were to gain an overview of the current roles and functions of nurses working in PHC settings - Health Centers (HC) and Health Posts (HP)-, to introduce nurse profiles for primary care potentially of relevance to Albania, to outline a roadmap for establishing updated nurse profiles in Albania and to exchange with the Faculty of Technical Medical Science of the University of Tirana (FTMS) on the planned master in family nursing.

Overall, we can conclude that the roles of nurses in PHC in Albania are varied. Nurses have different roles and scope of practice depending on their area of practice in the HC: in the Family Doctor (FD) consultation room, in special units, at the reception desk and in nurse-led HPs. Overall, nurses reported spending much time on administrative tasks or assisting the FD and less on direct clinical activities. They have a limited health promotion and education role, they have in general no autonomous role and few have a role training other nurses. Nurses working in the HPs reported a greater scope of direct clinical practice with less administrative tasks and a more clinical patient contact role.

Nurses in HC only occasionally perform a limited number of the clinical services outlined in the BPS. Nurses in nurse-led HP may have a broader scope of practice, but given the level of equipment and infrastructure and the local understanding of concepts (“clinical”, “physical assessment”, “palliative care”, etc.) these clinical nursing services are likely to be very basic. Overall, there is great potential to increase performance of nurses with regard to the following services: health education and health promotion services; preventive, self-management and chronic disease management services; and basic nursing care in the home.

In order to establish appropriate nurse profiles for PHC in Albania, we suggest a working group of key stakeholders performs a thorough context analysis, including stakeholder preferences, to define the responsibilities of all members of the primary care team in line with the PHC policy document elaborated in July 2019. In a next step, the key structure and processes of an Albanian PHC model can be based on the evidence, presented during our mission and the context analysis of the working group. This should be followed by a selection of implementation strategies and development of implementation materials to support the care processes. We suggest the working group consists of representatives of key stakeholders, including the Ministry of Health and Social Protection, the Order of Physicians, the Order of Nurses, the Health Insurance Fund, PHC providers (doctors, nurses, and managers), the FTMS, the patient population, the local/municipal authorities, the social services and the Health Service operator. We suggest working with a dedicated Chair and a core operational group preparing deliverables, will increase likeliness to achieve milestones and deadlines.

Given the relevance of a Family Nurse Professional Masters programme for PHC in Albania, the commitment of the key local stakeholders, the sustained request for support and the importance to establish good quality education for the implementation of the BPS, we recommend HAP to support FMTS extensively in establishing this professional master. A potential roadmap for the development of a relevant curriculum is included in the recommendations. Implementation of the new Curriculum by 2021-2022 ideally requires the nurse roles in PHC to be developed by late spring 2020.

# Background

Phase 2 of the Health for All project (HAP) supports the initiative of the Ministry of Health and Social Protection (MoHSP) and other health sector stakeholders to strengthen the nursing profession in order to address the future health challenges in Albania, especially the increasing burden of disease related to non-communicable diseases (NCDs) and the health care needs of the elderly and vulnerable population.

To tailor Primary Health Care (PHC) services to the prevailing burden of disease, roles and responsibilities of PHC personnel, particularly nurses, will be redefined and a professional master in Family Nursing (FN) will be established. HAP supports local stakeholders in both endeavours.

# Mission objectives and approach

The main objectives of this consultancy were:

- To revisit and update the overview of the roles and functions of nurses at Health Centers (HC) (including health posts HP) in Albania produced in 2016 and 2017
- To introduce nurse profiles potentially of relevance to Albania with specific consideration of PHC services and which are in line with the Basic Package of Services (BPS) at PHC level and the new PHC strategy of MoHSP which is currently under elaboration/finalisation
- To outline a roadmap for establishing updated nurse profiles in Albania with specific consideration of PHC services
- To discuss with the Faculty of Technical Medical Science of the University of Tirana (FTMS) key characteristics of the planned master in family nursing
- To summarize the findings and conclusions of the mission through a mission report.

In line with the approach described in the Terms of Reference (ToR), our report is based upon information collected through a number of activities, including:

1. ***Document review*** (available information on the HAP, the Albanian context, PHC and nursing in Albania, as well as relevant technical literature)
2. ***Preparatory Skype and onsite face-to-face meetings*** with the HAP implementation team in Albania
3. Development of a ***questionnaire and analysis of data*** regarding the role and activities of nurses in PHC in view of the nursing role included in the BPS
4. ***Individual meetings with representatives of key stakeholders***, including the MoHSP, the Order of Nurses, the Order of Physicians, and members of the Faculty of Technical Medical Science (FTMS) at the Tirana University
5. ***Workshops/Working Group Meetings*** including presentations of evidence and models and a discussion with representatives of the relevant stakeholder groups:
   1. *Workshop to validate quantitative survey data and collect additional qualitative data on the current roles of nurses* with 15 representatives of HCs and HPs from both the nursing (n=12) and medical profession (n=3)
   2. *Workshop on the development of the Curriculum for the planned Masters in Family Nursing* with 15 representatives of the Tirana University
   3. *Workshop on models of care for PHC and the overall process towards the development and implementation of new nurse profiles* with 10 representatives of PHC key stakeholders, including the MoHSP, the Health Insurance Funds, the Order of Nurses, the Order of Physicians, the FTMS at the Tirana University, PHC providers, and the Health Service Operator.

The mission in Albania was restricted to Tirana and lasted from Tuesday 10 September to Friday 13 September 2019.

At the end of the mission, first observations were shared in an in-person meeting with the Swiss Agency for Development and Cooperation at the Swiss Embassy in Tirana.

# Roles and functions of nurses: update

## Nursing role

There are different types of nurses and multiple ways to categorize the various nursing roles. Categorization of nurses and nursing types can be done using various factors:

- **Degrees and certifications:** One way to categorize nursing roles is by the level of education required for the position, including the degree (graduate, bachelor, master, doctoral) and type of certification needed. Higher degrees are related to more skills and competencies.
- **Population type:**  nurses can specialize in a particular segment of the population, e.g. paediatrics, geriatrics, psychiatry, women's health.
- **Medical specialty:** categorization of nurses can be based on their specialization in a particular field of practice, such as surgery, obstetrics, public health, ICU, or emergency medicine.
- **Location:** categorization of nursing roles can also be based upon practice location, e.g. school nurse, flight nurse, hospice nurse, hospital-based nurse, or home care nurse.

However, discussions on the type of nurses often draw from a mix of the different methods of categorization as they are not mutually exclusive. For example, a hospice nurse is expected to provide medical care and support to people in the end of life phase. The primary role is to ensure the patient is as comfortable as possible while attending to the emotional needs of the family and loved ones. Hospice nurses are often responsible for coordinating care between the different team members, including doctors, therapists, and dietitians. In addition to having extensive knowledge about terminal diseases, a hospice nurse must possess unflagging empathy for those dealing with end-of-life situations.

Regardless of the nursing types, the roles of the nurses can also be described based on the kind of activities that are being performed within a specific position.

- Some models refer to ***areas of nursing responsibility.*** Two such examples are the nursing role descriptions given by the Australian Primary Health Care Nurses Association (APNA) and the International Council of Nurses (ICN). The APNA describes the role of the PHC nurse with reference to: health promotion, illness prevention, midwifery, antenatal and postnatal care, treatment and care of sick people, rehabilitation and palliation, community development, population and public health, education and research, policy development and advocacy. According to ICN, “Nursing includes the promotion of health, prevention of illness, and care for the ill, disabled and dying people. Advocacy, promotion of a safe environment, research, participation in shaping health policy and in patient and health systems management, and education are also key nursing roles.” *(Bartz CC. International Journal of Integrated Care 2010)*
- Role descriptions sometimes include more ***generic functions.*** The CanMEDS framework which was originally developed for physicians, but which has been adapted for several other health care professions including nursing, is one such example. The framework identifies and describes the abilities health care professionals require to effectively meet the health care needs of the people they serve. These abilities are grouped thematically under seven roles, i.e. medical expert (the integrating role), communicator, collaborator, leader, health advocate, scholar and professional.
- More recent, increased attention is given by, among other, the World Health Organisation, and the Registered Nurses’ Association of Ontario, to the role of the ***nurse as part of a collaborative practice.*** This is understood as an interprofessional process for communication and decision making that enables the separate and shared knowledge and skills of care providers to synergistically influence the client/patient care provided. Nurses are an indispensable part of collaborative practices and multidisciplinary teams. These teams can have just as little as two different professions, i.e. a physician and a nurse, or can have a large variety of represented professionals, such as physical therapists, occupational therapists, speech therapists, psychologists or midwives.

## Findings previous missions

### Roles in HCs and HPs

During previous missions in 2016 and 2017, we obtained the following information on roles and functions of nurses through individual and focus group interviews of FNs, FDs and HC management and through limited direct observations:

- Few of the above suggested nursing roles are delivered by PHC nurses in Albania (who are educated to a minimum of BSc level) and nurses are not performing to their full potential.
- Nurses have different roles and scope of practice depending on their area of practice in the HC:
  - in the **FD consultation room** (mainly found in the HC), nurses:
    - spend 60% – 80% of their time on **administrative tasks** (reception of patients, finding patient files, writing patient details on prescription forms, completing (5-9) consultation registers)
    - spend the remaining part of their time mainly **assisting the FD** (handing over equipment and positioning patients)
    - have ***no or a very limited clinical role***
    - have ***no or very limited health promotion and education role***
    - have ***in general no autonomous role***
    - have ***no role training other nurses***
  - in **special units** which are often nurse-led and offer limited specific health services to a target patient population of the whole HC catchment (such as Children’s Health and Vaccination Unit (well children); Adult Screening Unit (adults 40-65 years old); Injection Unit; Emergency Unit; Women’s Health Unit)
    - Nurses appear to perform a ***limited range of specific predefined tasks*** rather than providing holistic patient care. The scope of these tasks is narrowly interpreted with limited application of recognised best practice standards. Thus, nurses working in the Health Screening Unit (Check-up Unit) read out the screening questionnaire word for word making no attempt to assess or assist patients’ understanding, record but do not react to answers indicating unhealthy or at risk behaviours, offer no advice or education and do not give patients health promotion leaflets. Nurses working in the Children’s Health and Vaccination Unit stated that on home visits they assess child development and motor skills by observation during a ten minute visit without the use of any recognised screening tools or methods (e.g. WHO growth charts).
    - During our site visits no health service users attended any of the special units supporting literature which reports limited patient numbers and the ***challenge of sufficient case load and regular clinical practice*** to ensure maintenance of clinical expertise in Albanian PHC.
  - In **nurse-led HPs**, where a nurse provides health care services to a population of 500-800, with a FD visiting occasionally
    - Nurses reported a ***greater scope of practice*** which was confirmed during interviews with other healthcare providers. However we did not observe this and could make no assessment of the actual scope or quality of care provided.
    - Overall, nurses reported less administrative tasks and a more clinical patient contact role. For the geographical population of the HP these nurses appear to undertake the specific tasks which in the HC are the responsibility of the special units e.g. child growth assessment and vaccinations, detection of pregnant women. They also mentioned performing duties (BP and blood sugar measurement, medication adherence, home visits, basic patient health status assessment, prevention and health promotion) which in the HCs are performed by the FD. They undertake these tasks autonomously, or with feedback and telephone support from FDs.
    - Nurses in HPs however do not provide basic nursing care in the home.
- Nurses working in nurse-led environments (particularly in nurse-led HPs) reported a greater level of autonomy and a wider and extended scope of practice than those working under direct physician supervision in the FD consultation rooms.

### Basic Package of Services (BPS)

The BPS includes Primary Health Care services funded by the Health Insurance Fund. The 7 service areas include: emergency care, child care, adult care, women’s health care and reproductive health care, elderly health care, mental health care, and health promotion and education. The *services* in the BPS are described at the level of the PHC facility (HC), and are not broken down along job profiles (FD and FN) or according to the context of service provision (e.g. nurse-led health posts). The BPS does outline the *required skills* for FD and FN.

In 2016-2017 the majority of the nurses and some of the FDs were unaware of the BPS and therefore had difficulty offering an opinion as to potential gaps without further assistance of the interviewers. When specifically asked about a number of generic nursing services, outlined in the different service areas of the BPS, the following information, which related to only 4 of the 7 service areas in the BPS, was given:

| Reported services | FN in HC | FN in HP |
| --- | --- | --- |
| Nursing Care in the home | No | No / occasional |
| Palliative Care | No | Occasional pain relief as per FD order |
| Patient health status assessment | No | Yes |
| Health Promotion Individuals  Health Promotion Community | No (or occasionally in absence of FD)  Sometimes (Diabetes Day, HIV in secondary School) | Yes  No |

## Findings current mission

### Methodology

Secondary data, obtained from a literature review and document analysis, was supplemented and compared with primary data collected in HCs in the Fier and Diber region prior to as well as during the mission.

Primary data was collected as below:

- ***Quantitative data*** was obtained using a self-reporting questionnaire proposed by the international experts (Deschodt & Van Malderen) in the Diber (municipalities of Diber, Mat, Bulqize and Klos) and Fier region (municipalities of Mallakaster, Patos, Lushnje and Fier). A representative sample of HCs and HPs was selected based on the size of the area covered (large and small areas), composition of the teams (mono and multidisciplinary) and location (rural and urban centers in both the north and south region). Data on the delivery of key services described in the BPS were obtained from 157 health professionals of which 132 were nurses, 4 were midwives and 21 were nurse-midwives.
- ***Qualitative data*** was obtained during a ***2 hour focus group*** discussion with a convenience sample of 12 PHC nurses from HCs and HPs who completed the survey questionnaire as well as 3 FDs. Goal of the focus group discussion was to validate and understand the quantitative survey data on the delivery of services as per the BSP.
- Further qualitative data was obtained from ***individual interviews*** with representatives of the MoHSP and the Order of Physicians.
- No data was obtained during this mission from observations of service delivery processes and infrastructure of the HCs and HPs.

This methodology was chosen in order to balance the limited duration of the mission with the need to triangulate and accommodate the natural biases and perspectives of individual informants.

We recognize the limitations of this methodology and the resulting findings, particularly:

- The limited opportunity for in-depth discussion with PHC nurses to assess the local understanding of key concepts (e.g. what constitutes a “patient health status assessment”; what constitutes “palliative care”; what constitutes “health promotion”, etc.).
- The lack of direct observations of nurses working clinically, delivering direct nursing care services, meaning that deductions about quality of nursing care, knowledge, understanding and interpretation of the meaning and requirements of clinical nursing services can only be deduced indirectly from stakeholder discussions.
- The mixed composition of the focus group. Although the presence of both practice nurses, head nurses and FDs enriched the discussion, it is possible that nurses might not have felt comfortable to share their opinion or express their needs in the presence of FDs.

### Findings

Annex E presents an overview of the information obtained through the survey.

Overall, in all service areas of the BPS the accurate completion of documents and records was reported as the most commonly conducted activity. Other services that were performed at least weekly by half of the nurses were use of the HC tools and equipment to provide necessary first aid in emergency situations, give advice to parents to ensure children’s well-being, measure and evaluate vital parameters of the patients, and administer treatment and medications to elderly patients.

Services that were the least often performed were CPR, administering appropriate medication in emergency paediatric cases, use of the peak-flow meter and microsurgery equipment, performing activities outside the HC for ensuring antenatal and postnatal care, performing rehabilitative post CVA services at the patient’s home, organizing periodic meetings with groups of elderly for health education and follow-up of chronic diseases, and organizing programs to raise community awareness on mental health problems.

The activities that were most often performed were in line with the activities the nurses felt most competent to conduct, i.e. administration and reporting, performing simple wound care, measuring vital parameters, giving vaccination and administering medications. The agreement among nurses on activities that were performed most often was high.

There was much more variety in activities nurses reported feeling less competent in, although the activities nurses report feeling less competent in mostly involve more complex nursing activities and skills, requiring clinical reasoning and health promotion and prevention activities in general.

Four of the six activities that are expected to be performed by nurses as part of the national check-up were conducted at least daily by half of the respondents. These included 1) giving support to the family doctor and contribute to informing citizens to go through check-up, 2) accurately complete the check-up forms, 3) counsel people according to the algorithms of the respective modules, and 4) ensure confidentiality and follow the ethical medical norms of the check-up examinations. A number of focus group participants disagreed with the fact that nurses acted upon the problems detected during the check-up. They felt that this was perceived as an administrative task and that most nurses do not use the information from the check-up to initiate interventions or make the appropriate referrals, indeed lack the knowledge and skills to understand and to act upon the information collected. This seemed to be a substantial problem also in the area of mental health.

In view of health education and promotion, the topics that were reported as being selected most often for activities or programs were related to breast and colon cancer, hypertension and smoking. In the focus group, participants indicated that the topics were selected either by the director or in consensus based on the health issues they felt were mostly encountered in the previous year. They indicated that these health education activities were not organized in a systematic way and that they lack background information and educational materials to perform these activities.

Half of the focus group participants reported that their role description was not clear and over half said to not feel competent to conduct all services described in the BPS. It was also mentioned that not all FDs give permission to the nurses to move outside the current supporting, more administrative role to perform all BPS services. This indicates a need for clearer role descriptions, interactions with FDs as well as continuous education initiatives for nurses employed in HCs and HPs.

An important difference was observed between nurses working in HCs and nurses working in HPs. HCs are mostly organized around multidisciplinary teams and a FD is present during working hours. Hence almost all nursing activities are performed under direct supervision. Outreach activities in patients’ homes are rarely conducted. HPs are mostly monodisciplinary organized and nurses working there estimated that they perform around 90% of their activities alone. In the remaining 10% they collaborate with a FD, either in a direct collaboration or by contact over phone. They indicated to spend around half of their time in the HP and about half in patients’ homes. Hence, nurses in HP still have a more autonomous role and a wider variety of frequently performed activities, within the HP as well as in the patients’ home.

It should be mentioned that the findings resulting from the current mission are more comprehensive in terms of the different service areas mentioned in the BPS and that the quantitative data are more generalizable given the large sample in comparison with the previous mission. However, both quantitative and qualitative data are based on self-reporting, so the data might not entirely reflect daily clinical practice. Site visits allowing for direct observations are to be planned in a next mission for further interpretation of the above-mentioned findings.

## Conclusion current nursing roles in Albania

Based upon the observations of the previous and current missions, we conclude that:

- The roles of nurses in PHC in Albania remain varied, with a clear distinction between the role in HCs and HPs.
- Nurses in HCs perform a more limited range of services outlined in the BPS and conduct the large majority in the presence of an FD in the HC. Home assessments or interventions are rarely done.
- Nurses in nurse-led HPs may have a broader scope of practice, but given the level of equipment and infrastructure, the local understanding of concepts (“clinical”, “physical assessment”, “palliative care”, etc.) these clinical nursing services are likely to be very basic. HP nurses spend as much time in the HP as in patients’ home.
- Role descriptions are not always clear to the nurses themselves.
- Overall, there is a clear underperformance and under-use of nurses with regard to the following services
  - Health education and health promotion services
  - Preventative and chronic disease management services
  - Basic nursing care in the home

# Nurse profiles for PHC in Albania

We conducted a workshop/focus group meeting on models of care in PHC and nurse profiles potentially of relevance for Albania. Participants included representatives of the main health care provision stakeholders, such as the MoHSP, the Order of Physicians, the Order of Nurses, the Health Insurance Fund, HC providers, the FTMS of the Tirana University and the Health Service operator.

The objectives of the workshop were (see Annex C: PPT Nursing Profiles in HCs and HPs in Albania):

- to refresh the core dimensions of the primary care process
- to present examples of nursing profiles in different PHC models
- to discuss the relevance and feasibility of evidence-based core components of PHC in the local context
- to discuss the potential roadmap to move the development and implementation of the PHC model of care forward

While presenting potential models and nursing profiles (the Family Medicine Model of the Republika Srpska, Bosnia and Hercegovina, a UK chronic care model for diabetes and the Buurtzorg model from the Netherlands) we emphasized that a model of care or nursing profiles cannot just be copy-pasted, and that adaptations of evidence-based core components of care models to the local context are needed.

A potential roadmap to develop and implement a care model for PHC in Albania was presented and discussed. Overall, we believe that the suggested roadmap was acceptable to the represented stakeholders, even though some participants were keen to move forward more quickly by skipping some preliminary phases. We therefore base our recommendations regarding the adaptation of nurse profiles in PHC in Albania (section 6) on the roadmap suggested during the workshop.

We had hoped to discuss with the workshop participants the evidence-based core components of care which are worth reviewing for the context of PHC in Albania. During the workshop, we touched upon a number of such components, including:

- Team and role changes towards effective, multi-disciplinary teamwork, with a shared understanding of common goals and clear competencies, roles and responsibilities of different team members and expansion of roles of personnel other than the primary care doctor
- Adequate resources (infrastructure and time)
- Case management: coordination of patient care by an individual other than the primary care doctor (e.g., arranging referrals, follow-up of test results, patient education, patient reminders)
- Promotion of self-management: Providing equipment (e.g., home glucometers for patients with diabetes) or access to resources (e.g., electronic systems for transferring data) and establishing joint goals to empower patients to manage their disease on their own
- Decision support: regular feedback (from registry data) to clinical teams on guideline compliance
- Patient navigator: Guide people through the health care maze, connecting them with the right doctors and helping them gain access to available therapies
- Outreach activities: Assessment, education or follow-up conducted outside the clinic or hospital, in or near the patient’s home.
- Patient education: Educating patients about their disease, including prevention and treatment strategies
- Patient reminder systems: Reminding patients about upcoming appointments or important aspects of self-care
- Clinician education: Educating clinicians about a particular condition or illness that their patients might face, including strategies for prevention and treatment (e.g., based on clinical practice guidelines); may be conducted through conferences, workshops, distribution of educational materials and one-on-one educational outreach meetings
- Clinician reminders: Reminding clinicians to look up patients’ clinical information or to conduct specific tasks.
- Audit and feedback: Generating summaries of clinic’s or individual clinician’s performance, which are transmitted back to the clinician
- Continuous quality improvement: Using specific processes to identify quality problems, developing solutions, and implementing and evaluating changes; may include interventions, such as total quality management or plan–do–study–act

Unfortunately, the time schedule did not allow for such an in-depth discussion on the relevance and feasibility of these evidence-based core components of PHC for the local context. This discussion will definitely need to be revisited as part of the roadmap activities.

Overall, the development of new nursing profiles seems to be quite feasible. From conversations with the MoHSP and the Order of Nurses, we understand that the legal and regulatory framework offers no barriers to a more autonomous role for nurses, and we observed an openness of the invited stakeholders towards the implementation of new nursing profiles within the existing BPS framework. As discussed in the previous section, there is a clear underperformance and under-use of nurses with regard to many of the services outlined in the BPS, including:

- Clinical services
- Health education and Health Promotion activities
- Preventative and Chronic disease management services
- Basic nursing care in the home

While new nursing profiles may include a substantial de facto role expansion for most nurses (and accordingly require substantial capacity building and change management), the BPS seems to provide a flexible framework for developing the preferred nursing role(s), thus allowing for a pragmatic approach to adapting actual profiles of health professionals in PHC without a great need for potentially lengthy adaptations to the legislation and professional regulation. Such role expansion also seems to be in line with the new PHC strategy for Albania, of which we got a preliminary version after our mission.

# Master in Family Nursing

The development and strengthening of Health Promotion and PHC services has become a cornerstone of the health system reform in Albania. Nurses form the largest group of health professionals and have a key role to play in PHC. Creating a Professional Master in Family Nursing is therefore highly relevant and an important means to support the effective implementation of the country’s PHC strategy in a sustainable way.

The initiative of establishing this new educational programme was taken by the FMTS of the University of Tirana with the Dean being a driving force. An inter-professional Working Group (with a majority of nurses – itself an innovative concept in Albania), has set itself an ambitious timeline and has produced first outputs.

From the previous and the current mission, we understand some work has been done so far (annex F and G), including:

- Identification of some gaps in local nurse knowledge and competencies („emergency“^^[[1]](#footnote-1)^^ care, communication, ethics, nursing processes and nursing care protocols)
- General objectives of the full-time 1 year MFN (60 ECTS) (2 x 15 = 30 weeks, of which 40% clinical practice)
- The title, aims, planned duration, brief outline of the syllabus and assessment methods of the 7 modules: (1) The Family Health Nurse: Concepts, Theory and Practice; 2) Provision of Care: Working with Families; 3) Decision-making; 4) Information Management and research; 5) Provision of Care: Working with Communities; 6) Managing Resources; 7) Leadership and Multidisciplinary working.

*It should be noted that this curriculum document is a one to one copy of the WHO (2000) Family Health Nurse Context, Conceptual Framework and Curriculum, with even the basic choices (e.g. assessment weight) left open and no adaptation to local needs and context*

- Overview of the number of ECTS obtained in various disciplinary fields
- Available faculty for the standard minimum of 20 students required for a Master Programme were said to be the current faculty of the FMFN plus clinical mentors.

During our mission, we conducted a focus group meeting/workshop with regard to the planned Master in Family Nursing. Although the Dean was not able to attend, the Vice-Dean, 8 nurse professors, 1 representative of MoHSP, 2 members of Order of Nurses, 2 HC nurses and 1 FD were present.

During the workshop, we presented and discussed a potential Curriculum Development process at FMTS (Annex D), with the following steps:

1. Programme development action plan
2. Evidence and standards
3. Stakeholder feedback
4. External and internal context analysis
5. Developing a blueprint
6. Developing the actual curriculum
7. Curriculum implementation plan

We particularly discussed the need to align the Curriculum content with the local context and the (to be) agreed model of care and nursing profiles for PHC in Albania.

From the discussion, we believe the group members support applying the suggested process for curriculum development and envision a timeline which would allow submitting the Curriculum to the appropriate University and governmental institutions by the submission deadline of November 2020. This would include an earliest implementation of the Curriculum from 2021-2022 onwards. This would allow for a number of accompanying measures, including faculty capacity building, organisation and preparation of clinical sites, tutors and placements as well as any other implementation supporting activities to be developed and implemented. At the same time, the November 2020 timeline allows for the extensive support we feel will be needed throughout the whole development process. The group members also clearly voiced the need to be supported in this process.

Based upon interviews executed through the local HAP implementation team, our discussions with the group members and the types of questions/nature of the expectations we were confronted with during the workshop, we feel support would need to include:

- Facilitation of the overall curriculum development and implementation process
- Methodological support to conduct the internal and external context analysis
- Technical support in specific areas (e.g. access to relevant evidence and standards; e.g. support with an organizing framework for key curriculum concepts, learning goals, meaningful assessment of clinical competencies)
- Capacity building to bridge competency gaps (nurse faculty as well as clinical mentors of future clinical placement sites)

Given the relevance of a Family Nurse Professional Masters programme for PHC in Albania, the vision and commitment of the key local stakeholders, the clear request for support and the importance to establish good quality education for the implementation of the BPS, we recommend HAP to support FMTS extensively in establishing a professional master on "Family Medicine nursing care".

In section 6 Recommendations, we formulate some concrete recommendations for possible future engagement of HAP.

# Recommendations

## Development of new nursing roles within the Albanian PHC model

### Potential roadmap for the development of new nursing roles

The existing BPS provides the necessary framework to develop the new nursing roles, as the foreseen nursing activities are already described there but are not yet (fully or partially depending on the type of activity) integrated in daily clinical practice.

Defining new nursing roles and profiles requires the existence and operationalisation of the new Albanian PHC model which is part of the overall PHC strategy elaborated in July 2019. Ideally, the development of the new nursing roles would therefore be integrated in the overall operationalisation of the new PHC model. In that case, the roadmap for developing a PHC model for Albania, including the development of new nursing roles, could look as follows::

1. Establishing a working group
2. Performing a context analysis
3. ***Defining responsibilities and roles of all members of the primary care team, including these of the nurses in HC and HP***
4. Defining the structure and processes of the Albanian PHC model based on the presented evidence, the context analysis and stakeholder preferences.
5. Selecting implementation strategies and development of implementation materials to support the care processes
6. Piloting the new PHC model in a limited number of sites focusing on acceptability and feasibility of the model
7. Scaling up and evaluation of the effectiveness of the model

In order for the Professional Master in Family Nursing to be developed and submitted by November 2020, we suggest that step 3 is finalised at the latest by May 2020.

If the above-mentioned approach might not be considered feasible, we alternatively suggest to narrow the scope of future missions and focus specifically on the development of new nursing roles related to 1) chronic disease management, 2) stimulating self-management activities in chronic disease patients, and 3) community-based health promotion activities. Although this would still need streamlining within the overall implementation of the PHC strategy, the success of the implementation will depend less on the other steps suggested in the roadmap above. Additional foci besides the three mentioned here can be integrated in the new Professional Master in Family Nursing so the new generation of the nursing workforce can further broader their scope of practice in line with local needs and preferences.

We suggest that the discussion around (future) home care services is in any case integrated in the discussions and decisions on the PHC model and profiles of nurses.

### Working group members

We suggest the working group consists of representatives of the stakeholders participating in the workshop: the MoHSP, the Order of Physicians, the Order of Nurses, the Health Insurance Fund, HC providers, the FMST of the Tirana University and the Health Service operator.

However, we suggest this group is enlarged by a representative of the patient population, a representative of the local government, as well as by a HP nurse, HC nurse and FD. Selecting the latter representatives from the future pilot sites may be beneficial in view of the later implementation phase.

To the extent that home care services would be integrated in the development of the PHC model, we suggest that the working group is extended with representatives of the relevant stakeholders in this area such as current social services and local and municipal authorities.

### Working group operations

We suggest the working group selects one committed and dedicated group member as the Chair / lead person who oversees and monitors the progress of the working group and can function as a first contact point for the local HAP team and any national or international external consultants. The presence of an internal facilitator, next to an external facilitator, has proven to be favourable to make sure milestones are achieved and the overall timeline can be respected, but also in view of successful implementation afterwards.

We suggest that one or more subgroup(s) are formed to prepare draft documents which can then be presented, discussed and agreed in the complete working group. Preparation of documents through a smaller core operational group lead by a committed chair of the Working Group will increase likeliness to achieve milestones and deadlines. If required, technical expertise can be provided in the form of document development workshops, as written feedback on documents, or as oral input during working group meetings.

A potential working group scheme in case of the first scenario suggested in 6.1.1 could be the following:

| Milestone | Timing | Local technical expertise | International technical expertise (on-site = OS; long-distance support = LDS) |
| --- | --- | --- | --- |
| Working group established | October 2019 | HAP |  |
| Context analysis performed | December 2019 | HAP | - Provide key questions to be answered (LDS) |
| Responsibilities of all members of the primary care team (HC and HP) defined | February 2020 | HAP | - 1 day workshop with responsible subgroup (OS - February)  - moderation 1/d day whole working group meeting (OS - February) |
| Structure and processes of the care model defined | June 2020 | HAP | - 2 day workshop with responsible subgroup (OS – May/June)  - moderation 1 day whole group meeting (OS – May/June)  - review report (LDS) |

A potential scheme in case of the second scenario suggested in 6.1.1 could be the following:

| Milestone | Timing | Local technical expertise | International technical expertise |
| --- | --- | --- | --- |
| HC / setting identified | October 2019 | HAP |  |
| Profiles and responsibilities of nurses of the primary care team (HC and HP) defined | December 2019 | HAP | - 2 day workshop with nurses (+FD) |
| Structure and processes supporting agreed nursing profile defined | Feb 2020 | HAP | - 2 day workshop with nurses (+FD) |
| Training workshop | June/July 2020 | HAP | - 2 day workshop with nurses |

## Development of Master in Family Nursing Curriculum

### Potential roadmap for the development of FN Curriculum

We suggest a roadmap for developing the FN Curriculum includes the following steps:

1. Programme development action plan
2. Review relevant evidence and available standards
3. Stakeholder input and feedback
4. External (e.g. community, policy) and internal (e.g. university, faculty) context analysis
5. Blueprint of the programme’s rationale, vision and mission
6. Curriculum content
7. Curriculum implementation plan

All of these steps are further explained in the appendix D.

### Working Group operations

We suggest that the working group is supported throughout the whole process.

Also here, we strongly suggest to appoint one dedicated group member as the main lead who oversees and monitors the progress of the working group and can service as a first contact point for the local HAP team.

A potential working group scheme could be the following:

| Milestone | Deadline | Local technical expertise | International technical expertise (on-site = OS; long-distance support = LDS) |
| --- | --- | --- | --- |
| Programme development action plan | October 2019 | HAP |  |
| Evidence and standards | November 2019 | HAP | Provide international literature (LDS – by November)  Provide key questions to be answered in context analysis (LDS – by November) |
| Stakeholder feedback | December 2019 | HAP | 1 day workshop to discuss context analysis and stakeholder feedback and WG report |
| First draft blueprint document | February 2020 | HAP | 1 day workshop |
| Final blueprint document and outline curriculum | June/July 2020 | HAP | 1 day workshop with responsible subgroup  1 day moderation whole working group  to agree blueprint, tasks and action plan curriculum |
| Curriculum | October 2020 | HAP | 3 day workshop with faculty (OS - August ) |
| Curriculum implementation plan | October 2020 | HAP | 1 day workshop implementation plan development – combined with curriculum workshop (OS - August) |

1. Terms of Reference
2. Mission Programme
3. PPT Nursing profiles
4. PPT FN Curriculum
5. Analysis Survey Nursing Roles
6. FTMS – draft curriculum
7. FTMS

1. Emergency care is a term often used by Albanian health professionals. It includes acute and non-acute medical emergencies, i.e. outside opening hours. They appear to form one of the key challenges for family nurses, given the limited/fixed opening hours of Primary Health Care Centres (8.00 – 14.40) and the number of nurse-only Health Posts in some of the less accessible regions. [↑](#footnote-ref-1)
