# Supplementary material for: Work time allocation at primary health care level in two regions of Albania
Source: PLoS One. 2022 Oct 26;17(10):e0276184. doi: 10.1371/journal.pone.0276184 (PMC9605026; doi:10.1371/journal.pone.0276184)
Supplement: Attachment — Submitted filename: Response to reviewers.docx [file pone.0276184.s007.docx]

**Response to reviewer, PLOS ONE**

The authors would like to sincerely thank the reviewer and the editor for their time and insightful comments and suggestions, which enable us to improve the presentation of our study and the quality of our manuscript. Please find below our point-by-point responses to the concerns raised by the reviewer and the editor and how we addressed these concerns.

| Reviewer 1 comments: | Response |
| --- | --- |
| The objective of this study is to assess the work-time allocation patterns of PHC workers in two regions of Albania and to compare the patterns between doctors and nurses and between urban and rural facilities. This study offers valuable insight on work-time patterns of doctors and nurses in urban and rural settings, in the two regions of Albania where the research took place | We thank the reviewer for recognizing the scientific value of the manuscript. |
| 1. For some activities the differences between rural and urban facilities are rather small, but the authors report that one group spends more time on the activity than the other group. For example:  line 230: “meetings (3.4% versus 3.3%)”. It is recommended that the size of the differences is also taken into account. | We acknowledge this comment and agree. Consequently reworded the respective section. We indicate now that for a number of categories differences between urban and rural being small, but also indicate those categories where differences are being observed (lines 234-242). |
| 2. Related to the previous comment, the observations are based on a sample. Is it not possible to calculate confidence intervals around the point estimates? | Thank you for bringing this important point to our attention. We now have calculated the confidence intervals for each of the categories of activities for doctors and nurses, in urban and rural settings, respectively. So not to overload the tables and to keep them well understandable, the confidence intervals are presented into three new tables as supporting information (reflected in the lines 508-513) under the titles: S4 Table, S5 Table, S6 Table. In text citation referring to the confidence intervals in supporting information are added (lines 212-213, 233, 241-242). |
| 3. The authors mention several limitations. It would be interesting to discuss more the implications of the limitations for the results. To what extent can the limitations influence the result? | We agree with the reviewer. Consequently we updated the limitation section accordingly (lines 326-327, 332-333, 337, 341-342) and hope it meets the reviewer’s requests. |
| 4. In each of the two study regions, nine health centers were included and at the health center level, one doctor, one nurse and one nurse at the affiliated health post-ambulatory. This should result in 54 HWs (2*9*3=54). However only 48 HWs participated in the study. Could be clarified which category of HWs are missing and why? I suggest to split in table 1 the category nurses in 'nurses at the health center' and 'nurses located at the affiliated health post-ambulatories'. | Thank you for this valid point and for the suggestion. We updated in the revised version of the manuscript table 1 and the narrative of the sampling so to better clarify why there is only 48 HWs and not 54. Indeed, there are only two urban health post ambulatories sampled per region, thus there are four nurses less from these facilities (changes in the lines 115-116). Further, we split the category nurses in four sub-categories in Table 1 i.e.: ‘nurses at urban health center’, ‘nurses at rural health center’, ‘nurses at urban health post-ambulatories’ and ‘nurses at rural health post-ambulatories’ (line 179). |
| 5. The added value of figure 2 escapes me since this information can also be found in table 2. Furthermore, the legend of figure 2 stated a category that cannot be found in the figure. | We would like to acknowledge reviewer’s observation about the similarities that figure 2 and table 2 present. However, we believe that figure 2 adds to the clarity and visibility of our manuscript, making it easy for the reader to interpret the data. We hope it being acceptable to keep both tables included.  Further, apologies for the typo in the legend. The category of activity from the legend of figure 2 the reviewer refers to in the comment, has been replaced accordingly (from ‘Direct patient care’ to ‘Service provision to users’). |
| 6. Table 2 is very important, however, the presentation of the categories is not clear. For example, the categories ‘other’, ‘meetings’, ‘administration’ are mentioned several times. | We thank the reviewer for this observation. We believe that this information can be found under the ‘Data collection section’ and we tried to further clarify it by adding an explanatory sentence (see lines 148-150). Further, we placed a footnote under Table 2 (lines 207-209) and we hope it meets the reviewer’s requests. |
| **7.** **Journal requirements:**  Please review your reference list to ensure that it is complete and correct. If you have cited papers that have been retracted, please include the rationale for doing so in the manuscript text, or remove these references and replace them with relevant current references. Any changes to the reference list should be mentioned in the rebuttal letter that accompanies your revised manuscript. If you need to cite a retracted article, indicate the article’s retracted status in the References list and also include a citation and full reference for the retraction notice. | Requirement acknowledged. We reviewed our reference list and there is no article that has been retracted so far. However, we did noticed that in the first submission we had missed to number one of the references (reference number 41, line 483). Subsequently, all the references after this one in the reference list, increased their number by one. Only one change was made in the text due to this (line 311). |
| 8. **Journal requirements:**  Please ensure that your manuscript meets PLOS ONE's style requirements, including those for file naming. | We thank the editor for bringing this point to our attention. We added a header to table 4 (line 248) in order to make the reading of the table more understandable. |
